# Constructing marine expert management knowledge graph based on Trellisnet-CRF

**DOI:** 10.7717/peerj-cs.1083

**Published:** 2022-09-05

**Authors:** Jiajing Wu, Zhiqiang Wei, Dongning Jia, Xin Dou, Huo Tang, Nannan Li

**Affiliations:** 1 School of Information Science and Engineering, Ocean University of China, Qingdao, China; 2 China Research Institute of Radiowave Propagation, Qingdao, China; 3 Chifeng University, Chifeng, China

**Keywords:** Marine expert, Knowledge graph, Entity recognition, Trellisnet-CRF

## Abstract

Creating and maintaining a domain-specific database of research institutions, academic experts and scholarly literature is essential to expanding national marine science and technology. Knowledge graphs (KGs) have now been widely used in both industry and academia to address real-world problems. Despite the abundance of generic KGs, there is a vital need to build domain-specific knowledge graphs in the marine sciences domain. In addition, there is still not an effective method for named entity recognition when constructing a knowledge graph, especially when including data from both scientific and social media sources. This article presents a novel marine science domain-based knowledge graph framework. This framework involves capturing marine domain data into KG representations. The proposed approach utilizes various entity information based on marine domain experts to enrich the semantic content of the knowledge graph. To enhance named entity recognition accuracy, we propose a novel TrellisNet-CRF model. Our experiment results demonstrate that the TrellisNet-CRF model reached a 96.99% accuracy rate for marine domain named entity recognition, which outperforms the current state-of-the-art baseline. The effectiveness of the TrellisNet-CRF module was then further demonstrated and confirmed on entity recognition and visualization tasks.

## Introduction

Expert talent is the main strategic resource of marine research think tanks, but effectively integrating these expert resources with the continued increase in marine research is a challenge. Knowledge graphs (KGs) have been widely used both in industry and academia to address real-world problems. There are two types of knowledge graphs: general and vertical-domain (Pujara et al., 2013). Vertical-domain knowledge graphs have a wide range of applications, such as in financial quantitative trading (Fan et al., 2017), patient information searches (Wu, 2021), intelligence education (Wan et al., 2018), historical research (Kim, 2017), and biomedicine (Lishuang, Honglei & Shanshan, 2016). General knowledge graphs are not domain-specific, so they cannot be used to create a marine domain expert knowledge graph.

There is a large amount of heterogeneous expert information data in marine science, and harvesting meaningful information from heterogeneous data sources is not a simple task (He & Dong, 2019). The existing database of expert material is full of both diverse and fragmented information, making accurate searches difficult (Wu et al., 2020; Liu & Yang, 2020; Li & Zhang, 2020; Ma et al., 2020). Knowledge graphs help address these obstacles by extracting the semantic information of the text data collected from all sources including institutional statements, scientific documents, and expert notes. A knowledge graph can provide a comprehensive conceptual description and presentation of expert topics in the marine domain.

Although the comprehensive knowledge graph architecture can achieve a preliminary construction of a marine field expert knowledge graph, it is still difficult to accurately identify and extract the many entities connected to experts in the field of marine science. With the rapid development of artificial intelligence, the significance of natural language processing (NLP) technology has been widely recognized in the industry (Csomai & Mihalcea, 2007). As the long short-term memory network (LSTM) method has excellent sequence modeling ability and automatically detects word-level features, it has been widely used for name entity recognition (NER) tasks, and most of the new methods of name entity recognition are based on LSTM models (Li et al., 2022; Chen et al., 2022; Rajput et al., 2022; Karim et al., 2022). However, as the inherent network architecture of the recurrent neural network, LSTM and recurrent neural network (RNN) variant models ignore the text structure and the semantic connection of the context, the word vector represented by the ordinary method is only the static word vector before the word-level information and does not contain the semantic information of the context (Ravenscroft, Goetze & Hain, 2022; Cui, Li & Xu, 2022; Li et al., 2021; Lang et al., 2021).

Unlike RNN-based networks, temporal convolutional network (TCN) architecture is informed by recent research and combines the best practices of modern convolutional architectures to convincingly outperform baseline recurrent architectures across a broad range of prediction tasks. In this study, we used a TCN variant called TrellisNet, which has all the advantages of a TCN, but increases the context recognition accuracy by combining input injection with nonlinear transformation (Bai, Kolter & Koltun, 2019). The conditional random fields (CRF) method is also commonly used for named entity recognition tasks (Guo, Qin & Li, 2013). Its advantage is that it can apply the information of the previously marked neighboring positions in the process of marking a position, and obtain the best sequence through decoding. We used CRF in this study to decode the output of the TrellisNet layer to obtain the best annotation sequence and improve the named entity recognition accuracy of the marine expert system.

Our study employs the merits of the CNN-based TrellisNet model to successfully construct a marine domain expert knowledge graph. The contributions of this study are as follows:

(1) We constructed a domain-specific knowledge graph based on an ocean expert domain ontology using dissimilar ontologies and semantic relations. (2) We proposed using the TrellisNet-CRF to improve the efficiency and precision of marine domain expert entity recognition (3) We conducted extensive experiments with both the marine domain expert dataset and the People’s Daily *corpus* dataset to test our proposal. The results of our experiments clearly demonstrated that the proposed TrellisNet-CRF model outperforms the other state-of-the-art methods. Moreover, the F1 value of the TrellisNet-CRF model reached 96.99% on the marine domain expert dataset.

## Background and related work

### Domain-specific knowledge graph

Since Google proposed the concept of a knowledge graph in 2012, a large number of enterprises and research institutions have explored knowledge graph research including YAGO (Hoffart et al., 2013), DBpedia (Lehmann et al., 2015), and XLore (Wang et al., 2013). These large-scale generic knowledge graphs collect a large amount of information, but there are still numerous areas not covered. With the maturity of related technologies such big data processing, researchers are increasingly turning to domain-specific knowledge graphs to meet industry needs.

The Allen Institute for Artificial Intelligence (AI2) has released a large-scale academic knowledge graph in three fields: computer science, neuroscience, and biomedicine to promote more research in these spaces (Ammar et al., 2018). Shanghai Jiao Tong University applied the AceKG large-scale academic knowledge graph (Wang et al., 2018) to the AceMap academic graph (Tan et al., 2016). The resulting graph covers multiple disciplines and includes 60 million scientific documents in 50,000 fields of research. This document dataset is open to the public to help researchers in the field of academic data mining.

The Open Academic Society has integrated Microsoft’s MAG (Sinha et al., 2015) and Tsinghua University’s Aminer (Tang et al., 2008) academic knowledge graph to build the Open Academic Graph (OAG), the largest open academic knowledge graph which includes 100 million scientific documents with a scale of 100 million. Abu-Salih (2021) and Abu-Salih et al. (2020) applied the learning and analysis of Socio-Political relationships using knowledge graph embedding technology. The academic data in these graphs are characterized by heterogeneous distribution, resulting in three main research difficulties: entity heterogeneity, entity ambiguity and large-scale matching (Cheatham, Krisnadhi & Amini, 2018; He, Sun & Wang, 2021).

### Application of deep learning in named entity recognition

With the development of computer and statistical applied mathematics, statistical machine learning (ML) methods became the mainstream method for handling named entity recognition problems. Traditional statistical ML algorithms, including the cascaded HMM network for Chinese entity recognition, the support vector machine (SVM) for sequence labeling (Shankar et al., 2020), and the maximum entropy model (MEM) can integrate entity recognition (Evans & Prokopenko, 2021) and constraint information into the NER to improve the accuracy of NER experiments. CRF (Tang et al., 2020) combines the structural and distance dependencies of words, optimizes the label output, and achieves good performance on named entity recognition tasks in various fields.

Traditional ML methods require a large amount of manually annotated *corpora* for feature extraction, which is time-consuming and labor-intensive. Because knowledge graphs need to integrate knowledge quickly, the performance of traditional ML methods for knowledge graphs is limited. In the process of building knowledge graphs, new research methods need to be introduced to improve the efficiency of knowledge integration. The emergence of deep learning frameworks has accelerated the creation of many new named entity recognition and entity relation extraction methods. As long short-term memory network (LSTM) has excellent sequence modeling ability and automatically detects word-level features, LSTM is widely used in NER tasks, and most of the new NER methods are based on the LSTM method.

Ma & Hovy (2016) later introduced a novel neural network architecture by combining BiLSTM, CNN and CRF, which can automatically improve model performance from word-level and character-level representations. These end-to-end neural network structures could automatically learn from textual information to improve entity recognition efficiency in NER tasks (Gao, 2017). While LSTM used with recurrent neural network (RNN) variant models can ignore the text structure and context, the word vector represented by the ordinary NLP method is only a static word vector without any word-level information or context. Compared with a traditional recurrent neural network, Bai, Kolter & Koltun (2018) proposed a parallelized temporal convolutional network (TCN) to solve the problem of training long sequence text. Notably, the TrellisNet network, a variant of the TCN network, not only has all the advantages of convolutional neural networks but also takes advantage of nonlinear relationships of text through input injection and weight sharing mechanisms (Bai, Kolter & Koltun, 2019).

## Marine expert management knowledge graph architecture

### Ocean expert knowledge graph architecture

**Figure 1:**
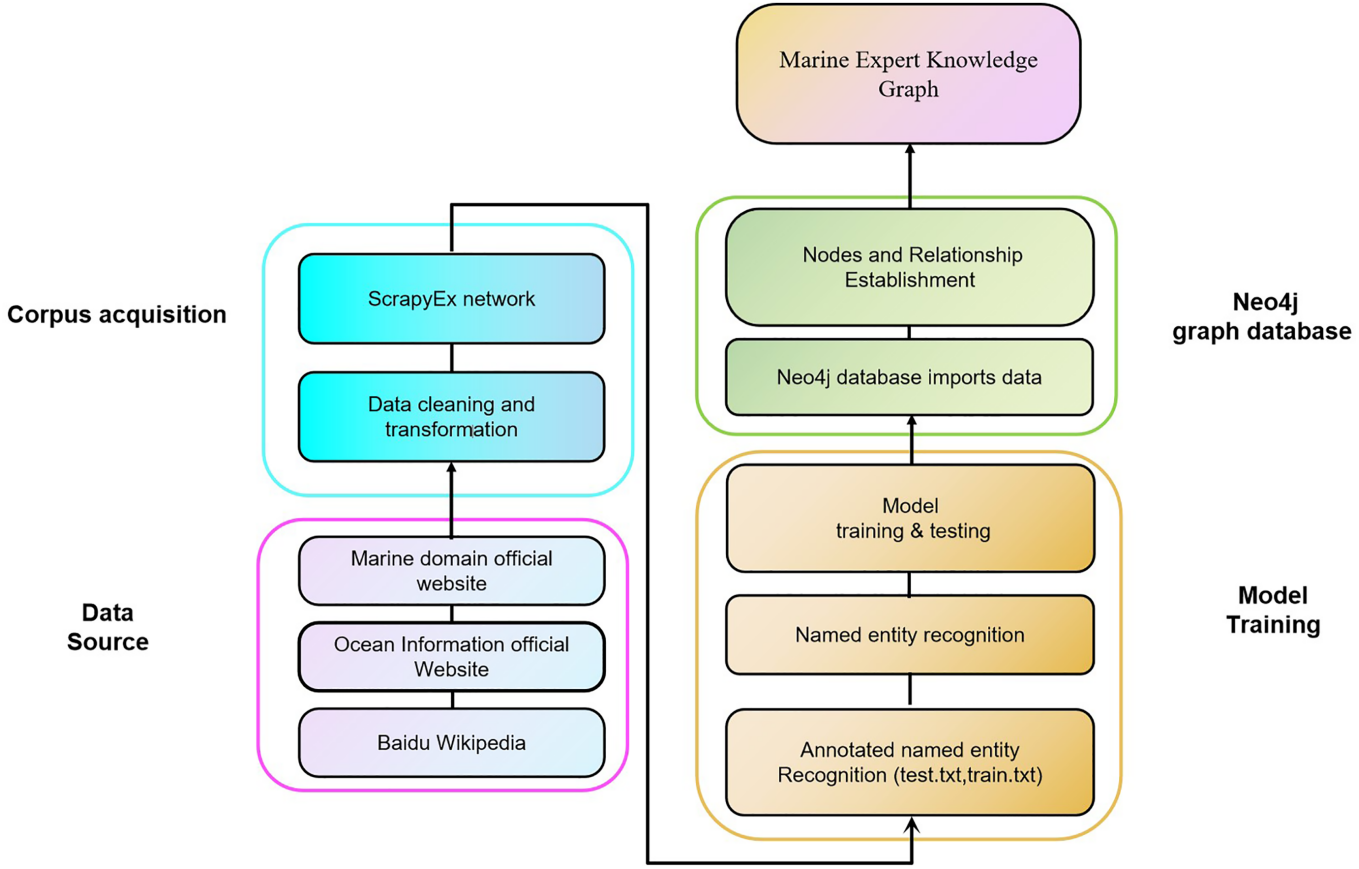
Expert knowledge graph flow chart.

Figure 1 illustrates a flow chart for establishing a knowledge graph for marine experts in China. The first step was to collect both unstructured and structured data from publicly accessible sources, such as official marine information websites, the Baidu Encyclopedia, public reports, social media in the marine field, and scholarly marine science literature. Next, we carefully sorted and thoroughly cleaned the raw *corpus* data using ScrapyEx to convert dynamic web content into local content to ensure the accurate retrieval of information. For large amounts of text, we analyzed and cleaned the content, retaining relevant information and discarding information not relevant to marine science. Next, we stored the clean data in the SQL database. The third step was to label the well-organized marine domain expert entity *corpus*, and divide the data set into a training set and a test set to train and test the model. The proposed TrellisNet-CRF network was applied to train the classification model, and the model training results were adjusted for model parameters. The optimal prediction model was reserved, and new entity pairs were automatically extracted and identified (Xu, 2018). After the entity recognition step, the marine expert knowledge graph automatically acquired new entities from the source data. Finally, we used the Neo4j graph database, which enables users to quickly obtain and understand knowledge structure information, to store and present the entity and relationship information of marine field experts. As a typical representative of a graphic NoSQL database, Neo4j can conveniently and stably store and manage hundreds of millions of nodes and relationships using the APOC tool and CSV file loading.

### Marine expert knowledge graph semantic model architecture

**Figure 2:**
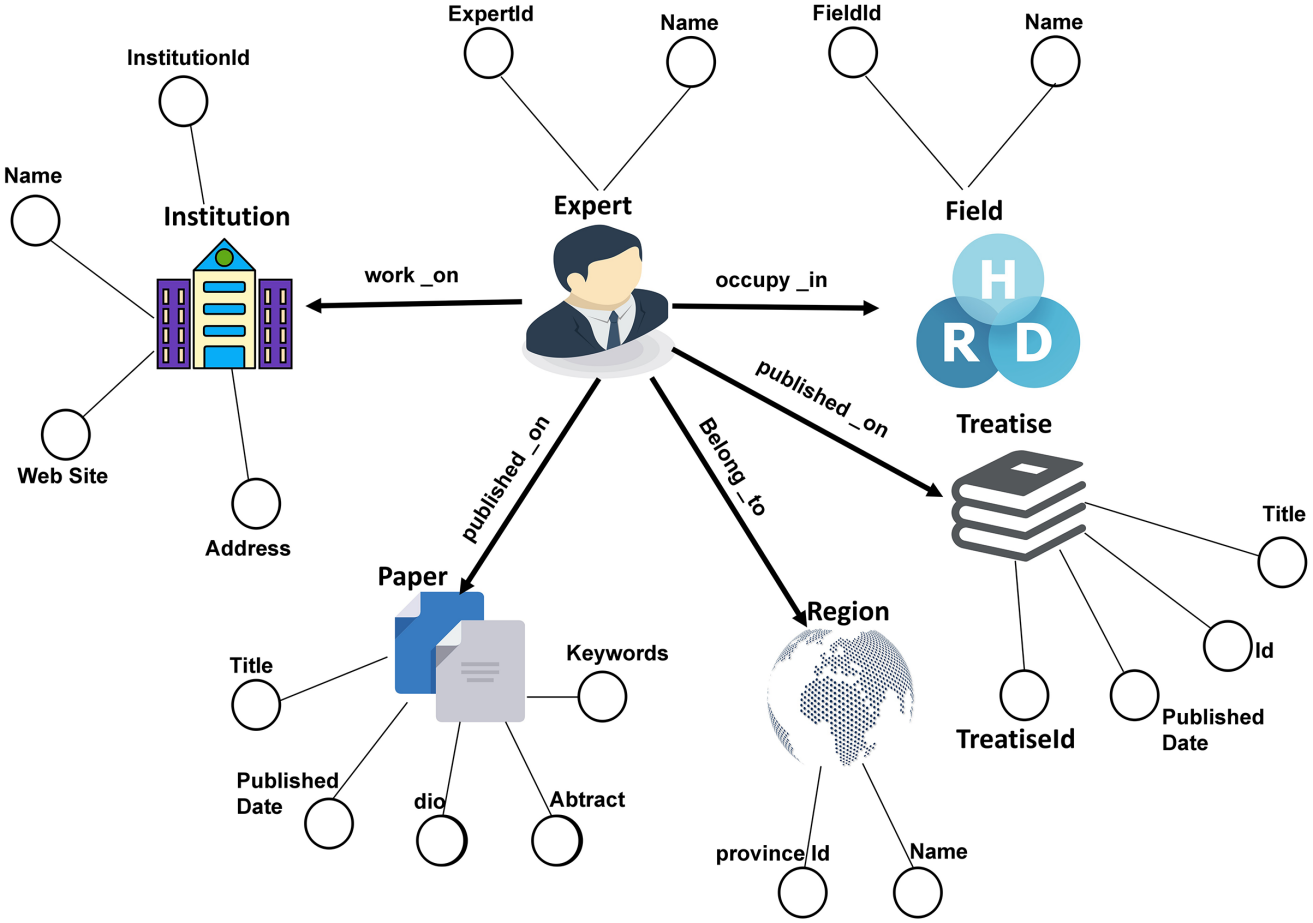
Expert knowledge graph semantic model structure.

**Figure 3:**
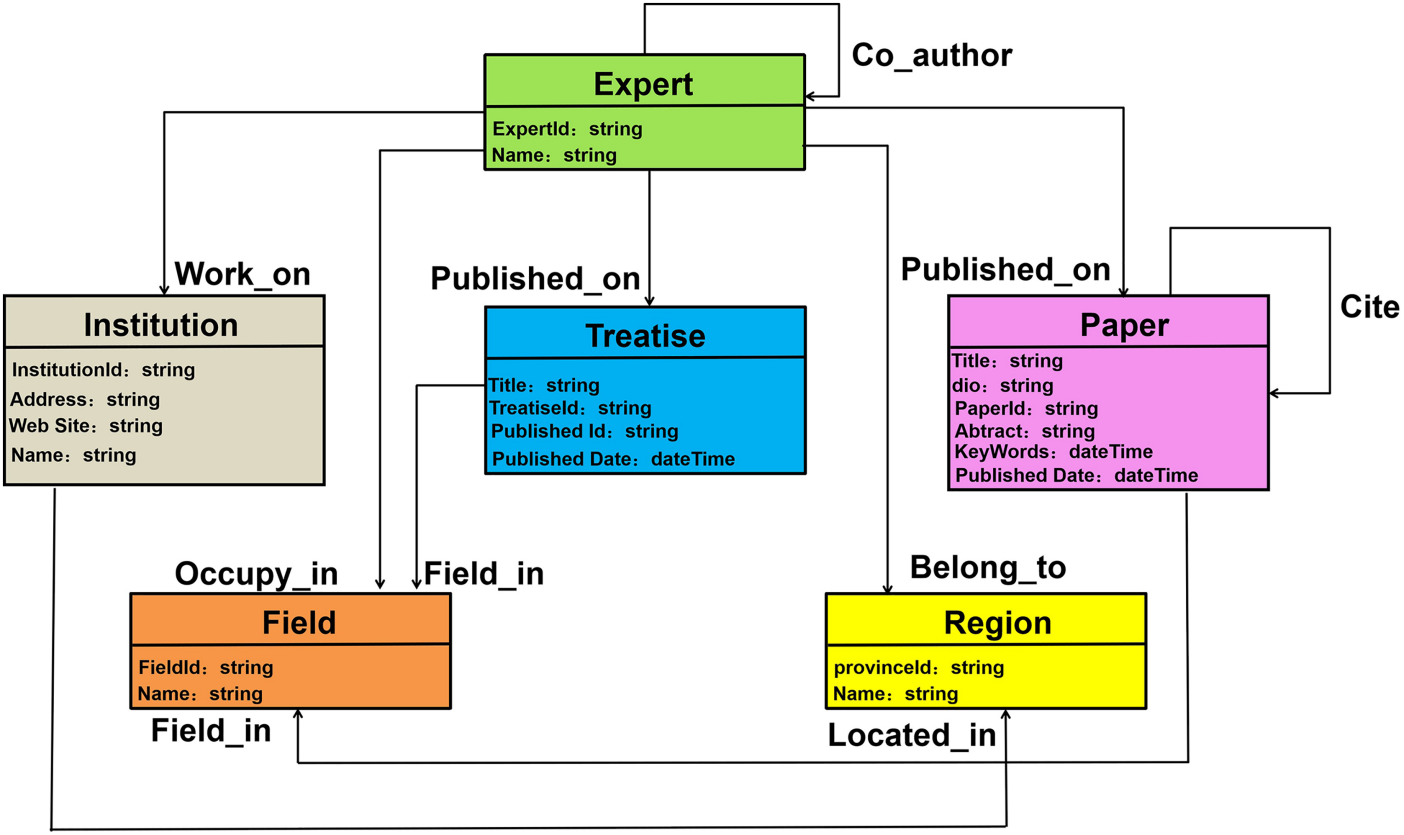
Layer design of expert knowledge graph pattern.

The academic knowledge graph of marine experts we created is a heterogeneous network, with nodes and edges representing multiple types of academic entities and relationships. As demonstrated in Fig. 2, the model layer defines the six types of academic entities: Expert, Paper, Institution, Region, Treatise, and Field. Since UML is a unified and standardized modeling language, it can organize and analyze the entities and related relationships of the overall marine domain expert knowledge graph. For this reason, we used the UML modeling language to model entities and relationships in this study. The design of the UML mode layer is shown in Fig. 3. There is a hierarchical structure between academic entity classes, which defines six academic entity categories (Expert, Institution, Treatise, Paper, Field, Region) and 10 academic relationship types including: cooperation, work on, occupy in, field in, belong to, and located in.

As shown in Fig. 3, we defined six entities, and eight relationships. Among them, “Expert” represents an expert entity node. The attributes of each expert include: expert id, expert name, publishing relationship, cooperative relationship, and the institution and province where the expert works. The “Paper” entity node represents scientific papers written by the expert. The paper entity node includes the paper ID, title, abstract, keywords, and publication date. Paper relates to the marine domain Expert research field. There is a citation relationship between Paper or Treatise, and the citation relationship has multiple pairs of mapping properties. “Institution” represents the institution where the expert works. Institution includes the name and id of the institution, the address where the institution is located, and the website published by the institution. Each expert may work in one institution or belong to two institutions. “Treatise” means a treatise or book written by an expert. The attributes of each Treatise include: name, treatise id, publication id, and publication time. “Field” is the research direction that the expert has been engaged in for their career. The attributes of the Field include the field id and the name of the field. The paper and treatise of the expert can infer the specific research field of the expert. “Region” represents the geographic information of the province and city where the expert is located. The information of each Region contains the province and city id and province name.

## Trellisnet-crf network for named entity recognition

In this section, we provide an overall description of the named entity recognition deep learning framework proposed in this article. Then, we describe the construction process and method of the TrellisNet network in detail. Finally, we introduce the construction method of the CRF network.

### Overall TrellisNet-CRF network

**Figure 4:**
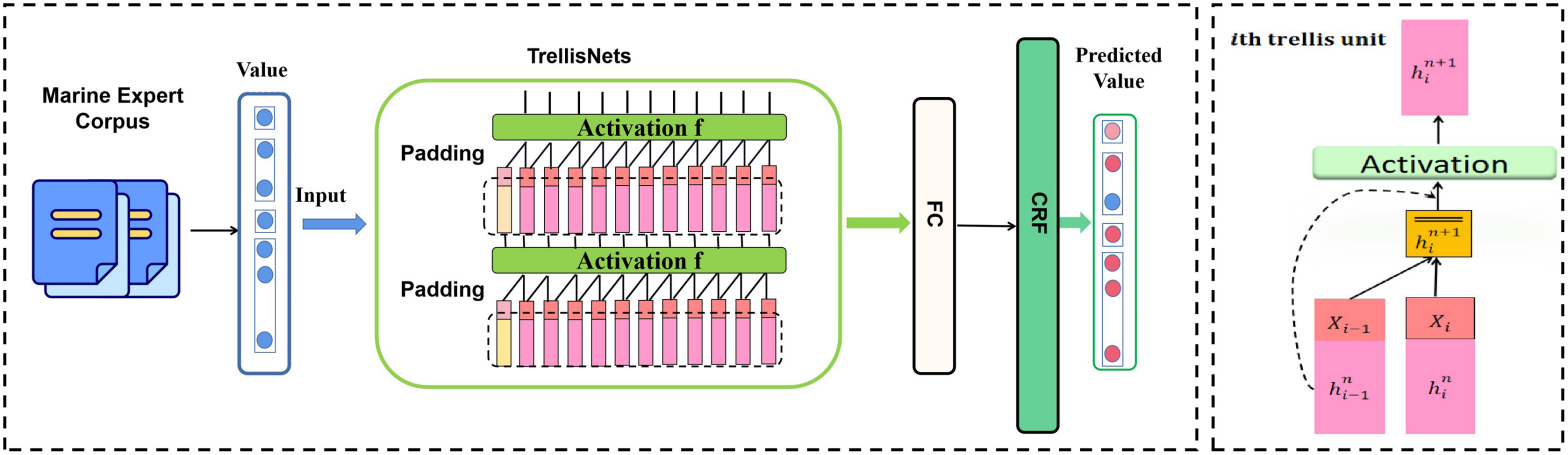
The overall architecture of Trellinet-CRF.

The overall TrellisNet-CRF framework is demonstrated in Fig. 4. The proposed model was mainly composed of two modules: TrellisNet and the CRF module. First, we input the cleaned ocean domain expert entity *corpus* vector into the TrellisNet network for named entity recognition training. Then, we used the CRF network to optimize the results of the TrellisNet model and to obtain a predicted label sequence. Each entity was then classified to complete the task of named entity recognition for marine experts. The detailed process consisted of the following steps:

(1) Input layer: Input the labeled marine-domain expert *corpus* into the TrellisNet-CRF network.

(2) Encoding prediction layer: Obtain the probability of the entity label corresponding to each entity through TrellisNet processing.

(3) Label decoding layer: Further optimize the results obtained by TrellisNet for label decoding by CRF.

(4) Iterative training, saving the optimal model, and verifying the performance of the model and the accuracy of entity recognition through the test dataset.

### TrellisNet network

In this study, we propose a novel temporal convolutional network characterized by weight sharing and input injection, also applying a nonlinear transformation called TrellisNet to capture the correlation of the word space vector in the process of named entity recognition. Convolution architecture with a weight sharing mechanism creates a stable gradient, has a fast training speed and an exponentially large receptive field size, which can handle long history word space vectors better than cyclic architecture. Input injection combines the depth features with the original input sequence, and then performs a nonlinear transformation, which improves the performance of the temporal convolutional architecture to capture nonlinear associations.

For the process of named entity recognition in the entire ocean domain, at the interval a of the sequence, we applied an m-dimensional vector ${X_a} = \left( {f_{1,a}^{out},f_{2,a}^{out}, \ldots ,f_{m,a}^{out}} \right) \in {R^m}\;$to represent the m-dimensional vector of the word space.

To predict the output of the expert’s entity at interval *a+1*, we used the word vector in the past *A* interval as input. As a result, we used an output data sequence of length *T* as the output of the TrellisNet network, represented by:

(1) $${X_{1:A}} = \left( {{X_{a - ( {A - 1} )}},{X_{a - ( {A - 2} )}}, \ldots ,{X_a}} \right).$$

As shown in Fig. 4, a complete TrellisNet network is composed of a basic unit that spans the superposition of steps and layers. As shown on the right side of Fig. 4, the $i$th unit of the layer *m* of the grid is composed of the hidden outputs $h_{i - 1}^n \in {R^m}$ and $h_i^n \in {R^m}$ from the previous layer $n + 1$ and the vectors ${X_{i - 1}}$ and ${X_i}$ injected from the input sequence. The composition, or the transformation in the $\; i$th unit is defined as follows (2):

(2) $$\left\{ {\matrix{{{\mathop h\limits^ =}_i^{n + 1} = {W_1}\left[{X_{i - 1}}|\left|\right.h_{i - 1}^n\right] + {W_2}\left[{X_i}|\left|\right.h_i^n\right]}\cr {h_i^{n + 1} = f\left( {{\mathop h\limits^ =} _i^{n + 1},h_{i - 1}^n} \right)}}} \right..$$

In the vector of the input sequence words, || presents series operation, $\; {W_1}$ and ${W_2}$ are kernel weights, $\; h_i^{n + 1} \in {R^m}$ is the output of the $i$th unit in layer $n$, and $f$ is a nonlinear activation between $h_i^{n + 1}$ and ${\mathop h\limits^{=}} _i^{n + 1}$.

We used the same weight matrix to apply the above transformation process to all time steps and iterations of all layers, as shown in Fig. 4. Given the input spatial vector word data sequence, the calculation of each layer of TrellisNet can be summarized as:

(3) $${\rm \; \; \; \; \; \; \; \; \; \; \; \; \; \; \; \; \; \; \; \; \; \; \; \; \; \; \; \; \; \; \; \; \; \; \; \; \; \; \; }h_{1:A}^{n + 1} = f\left( {\left( {h_{1:A}^n||{X_{1:A}}} \right)*W,h_{1:A - 1}^n} \right).$$

where * represents a one-dimensional causal convolution operation, applying zero padding convolution to convolve the output of the previous layer with the data of the past interval, and *W* represents the kernel weight matrix parameter shared by all layers.

**Figure 5:**
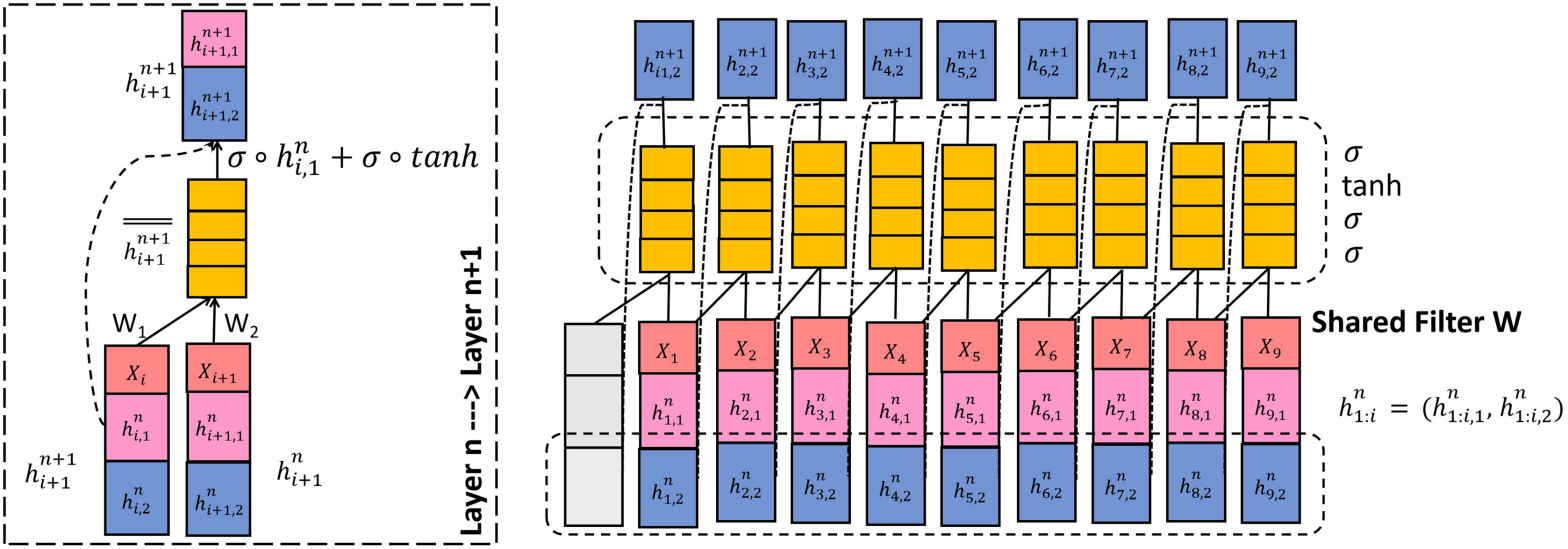
A TrellisNet with an LSTM nonlinearity gate activation.

To ensure the TrellisNet network prevents overfitting and gradient explosion during training, in this study, we applied a gated activation mechanism based on LSTM. In the gate activation mechanism unit of LSTM, three information control gates are calculated in sequence $i$. In addition, there is a cell state that does not participate in the hidden-to-hidden transition but is updated with the result of the gated activation at each step. We integrated the LSTM cell into TrellisNet, as shown in Fig. 5:

$f_{\rm i}^n = \delta \left( {{W_f}K_i^{n - 1} + {U_f}k_{i - 1}^n} \right);{\rm \; }$

${\rm \; }i_i^n = \delta \left( {{W_i}K_i^{n - 1} + {U_i}k_{i - 1}^n} \right);{\rm \; \; }$

${\rm \; }g_i^n = tanh\left( {{W_g}K_i^{n - 1} + {U_g}k_{i - 1}^n} \right);$

${\rm \; }o_{\rm i}^n = \delta \left( {{W_o}K_i^{n - 1} + {U_o}k_{i - 1}^n} \right);{\rm \; \; }$

${\rm \; }c_{\rm i}^n = {\rm \; }f_i^n \circ c_{i - 1}^n + i_i^n \circ g_i^n;{\rm \; \; \; \; \; }$

(4) $${\rm \; \; \; \; \; \; \; \; \; \; \; \; \; \; \; \; \; \; \; \; \; \; \; \; \; \; \; \; \; \; \; \; \; \; \; \; \; \; \; \; \; \; \; \; \; \; \; \; \; \; \; \; }k_i^n = o_{\rm i}^n \circ \tanh\ \left( {c_i^n} \right){\rm \; \; \; \; \; \; \; \; \; \; \; \; \; \; \; \; \; \; \; \; \; \; \; \; \; \; \; \; \; \; \; \; \; \; \; \; \; \; \; \; \; \; \; \; \; \; \; \; \; \; \; \; \; \; }$$

where ${\rm \; }k_i^0$ = ${x_i}$, and ${f_i}$, ${i_i}$, ${o_i}{\rm \; }$are called the forget, input, and output gates.

Figure 5 shows a cell, and a sequence view of TrellisNet with the LSTM gate activation. There are two parts in hidden cell $h_{1:i}^n$: $h_{1:i,1}^n$ gets updated directly based on the gated activations, and $h_{1:i,2}^n$ gets updated based on LSTM hidden states.

(5) $${\mathop h\limits^{=}} _{1:{\rm i}}^{n + 1} = Conv1D\left( {h_{1:i}^n;W} \right) + {\tilde x_{1:i}}{\rm \; \; } = {[{\tilde h_{1:i,1}}{\rm \; \; \; }{\tilde h_{1:i,2}}{\rm \; \; \; }{\tilde h_{1:i,3}}{\rm \; \; \; \; \; }{\tilde h_{1:i,4}}]^T}{\rm \; \; \; \; \; \; \; \; }$$

(6) $${\rm \; \; \; \; \; \; \; }h_{1:{\rm i},1}^{n + 1} = \delta \left( {{{\tilde h}_{1:i,1}}} \right){\rm \; } \circ {\rm \; }z_{0:i - 1,1}^n + \delta \left( {{{\tilde h}_{1:i,2}}} \right) \circ \tanh \left( {{{\tilde h}_{1:i,3}}} \right)$$

(7) $${\rm \; \; \; \; \; \; \; \; \; \; \; \; \; \; \; \; \; \; \; \; \; \; \; \; \; \; \; \; \; \; \; \; \; \; }h_{1:{\rm i},2}^{n + 1} = \delta \left( {{{\tilde h}_{1:i,4}}} \right){\rm \; } \circ \tanh \left( {{\rm \; }h_{1:{\rm i},1}^{n + 1}} \right).$$

Therefore, the linear transformation in each layer of the TrellisNet produces a pre-activation feature with four feature channels, which are then processed by elementwise transformations to yield the final output $h_{1:{\rm i}}^{n + 1} = {\rm \; }h_{1:{\rm i},1}^{n + 1} + h_{1:{\rm i},2}^{n + 1}$ of the layer.

### CRF network

The CRF network serves as a probabilistic undirected graph model used to annotate or analyze sequence information. The CRF model can express long-distance dependent features the CRF model can express long-distance dependent features in order to compute the optimal joint probability in long sequences. This hybrid model uses the CRF layer to decode the output of the last fully connected (FC) layer to obtain the optimal annotated sequence.

If $z = \left\{ {{z_1},{\rm \; }{z_2}, \ldots ,\; {z_n}} \right\}$ represents the input sequence of the sentence, and ${z_i}$ is the word vector of the ${\rm \; }i{\rm \; }$word in that sentence; $y = \left\{ {{y_1},{\rm \; }y, \ldots ,\; {y_n}} \right\}$ represents the label sequence of the sentence z, and ${y_{\left( z \right)}}$ represents the set of all possible label sequences of the sentence $z$. The specific form of the CRF probabilistic model is:

(8) $$p\left( {y{\rm |}z;W,b} \right) = \displaystyle{{\mathop \prod \nolimits_{i = 1}^n {\varphi_i}\left( {{y_{i - 1}},{y_i},z} \right)} \over {\mathop \sum \nolimits_{{y}^{\prime} \in y\left( z \right)} \mathop \prod \nolimits_{i = 1}^n {\varphi_i}\left( {y_{i - 1}^{\rm '},y_i^{\rm '},z} \right)}}.$$

where ${\varphi_i}\left( {{y}^{\prime},y,z} \right) = \exp \left( {W_{{y}^{\prime},y}^T\ {z_i} + {b_{{y}^{\prime},y}}} \right)$, and $W_{{y}^{\prime},y}^T$ and ${b_{{y}^{\prime},y}}$ represent weight vector and bias transferred from label ${y}^{\prime}$ to label $y$, respectively.

During training, the log-likelihood function is as follows:

(9) $$L\left( {W,b} \right) = \mathop \sum \nolimits_i logp\left( {y{\rm |}z;W,b} \right)$$

According to the maximum-likelihood estimation principle, maximizing the log-likelihood function is to maximize the CRF conditional probability model. The optimization objective function is:

(10) $${y^{\rm *}} = argmaxp(y|z;W,b).$$

The CRF layer receives the hidden state sequence of the TrellisNet layer as input and improves the accuracy of entity recognition by learning the relationship between adjacent labels.

## Experiment results and analysis

### Dataset

Table 1 shows the verification of the effectiveness and generality of the named entity recognition method. This study used the People’s Daily *corpus* and the marine expert dataset as the experimental datasets. Both the People’s Daily *corpus* and the marine expert dataset are datasets marked with BIOS and have the same structure.

**Table 1:** Experiment dateset.

*Corpus* dateset	Training dateset	Test dateset
People’s Daily c*orpus*	17,573	1,718
Marine expert c*orpus*	26,093	2,609

The People’s Daily *corpus* is the largest public evaluation dataset in China. It is based on the 1998 *corpus*, and after extensive processing work, is now a large-scale, high-quality *corpus*. The People’s Daily *corpus* includes: person name (PER), place name (LOC) and organization (ORG).

Since marine domain experts do not have publicly available *corpus* data to train models, the basic data of the experiments used in this study came from the constructed marine domain expert *corpus*. We extracted and collected unstructured and structured data from official marine information websites, the Baidu Encyclopedia, public reports, social media in the marine field, and relevant academic marine science literature. We used a wrapper to obtain the semi-structured data from HTML pages and convert it into structured data. We also used NLPIR natural language processing tools to perform word segmentation on unstructured data to extract domain-specific information. High-quality annotated *corpus* resources are lacking in the field of marine science. Therefore, this experiment crawled related terms from Baidu Baike (baike.baidu.com) and marine science websites (https://www.ckcest.cn/home/center/oce; https://book.oceaninfohub.org/index.html) for manual text annotation. The *corpus* acquisition experiment in this article adopted the distributed and GPU-accelerated crawler framework Scrapy, which has stable performance, supports breakpoint saving, and supports multi-threaded parallel crawling. The PDF files of academic marine science literature were transferred to the MySQL database for preservation. For large amounts of text, we analyzed the content of the text, retaining information pertinent to the marine science domain, and discarding information that was not relevant.

### Named entity recognition analysis

To evaluate the results of the TrellisNet-CRF model, we collected annotated datasets of marine experts. For a fair comparison of our proposed method with state of-the-art models, we employed three named entity methods: expert name (PER), organization name (ORG), and location (LOC). The computer system we used was a Windows Server 2016 Standard with Intel(R) Xeon(R) CPU E5-2650 V4@2.20 GHz and two TESLA GPUs. The two GPUs were used to train the system. This study divided these three types of named entity data sets into two parts: a training set (90%) and a test set (10%).

a. Labeling mode

When utilizing machine learning for natural language processing tasks, for the machine to “understand” the structure of the sentence, the sentence must be labeled. In this study, we used the BIOES mode to mark the text. For instance, this model uses E-label to represent the end of the entity, and S-label to represent the single entity. To mark the sentence “Expert Jack and Expert Zhang San are in Shandong,” the BIO mode marks the sentence as: “B-PERO B-PER I-PER OO B-LOC” and the BIOES marks the sentence as: “S- PER O B- PER E- PER OO S- LOC.”

b. Pre-trained word vector

As Chinese text is a kind of hieroglyphics compared with English and other pinyin texts, the NER task for Chinese faces a major obstacle: there are no spaces in Chinese texts, so spaces cannot be used as natural separators of words. Because of this, it is impossible to directly input Chinese words into the model as in English as the labeling mode does not work to label Chinese words. It is therefore necessary to segment the text and input the text into the model after generating the word vector. We used Facebook’s open-source text classification tool, fastText, to preprocess the data text and represent the words as word vectors in a 200-dimensional space.

c. Model parameters

We input the pre-trained word vector into the model for training. The models and hyperparameters used in this study are shown in Table 2. The parameters in the TrellisNet-CRF model are trained to maximize the log probability of the NER-labeled observation sequence in the annotated *corpus*. In the forward propagation process, we obtained the context representation from the expanded convolutional layer, and then used Viterbi decoding to find the optimal tag sequence. We then calculated the accuracy rate P, the recall rate R and the F1 value to evaluate the NER quality of the model corresponding to the three named entities. The specific formula we used is as follows:

**Table 2:** Trellisnet model parameters.

Parameters	Value
Optimizer	Adam
Initial learning rate	0.001
Hidden size	500
Embedding size	200
Embedding dropout	0.0
Hidden (VD-based) dropout	0.3
Output dropout	0.1
Weight dropout	0.25
Auxiliary loss λ	125
Auxiliary frequency	0.3
Weight normalization	True
Gradient clip	0.2
Weight decay	0.000002

(11) $$P = \displaystyle{{TP} \over {TP + FP}}$$

(12) $$R = \displaystyle{{TP} \over {TP + FN}}$$

(13) $${F_\rho } = \displaystyle{{\left( {{\rho ^2} + 1.0} \right) \times P \times R} \over {{\rho ^2} \times P + R}}$$

where TP is a real case, FP is a false positive case, FN is a false negative case, and the value of ρ is set to 1, which means that the accuracy rate and the recall rate are of the same importance.

d. Named entity recognition result analysis

**Figure 6:**
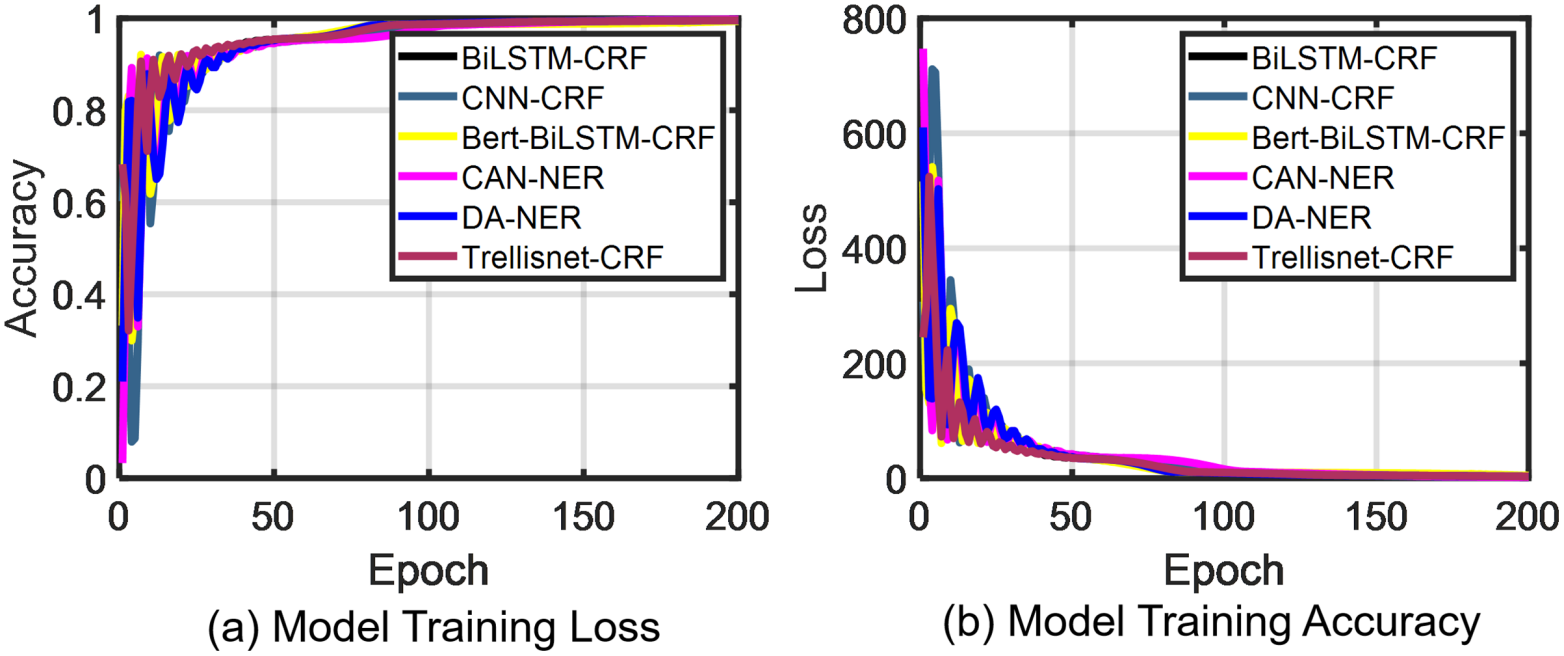
People’s Daily *corpus* test results.

As shown in Fig. 6, we verified the stability and convergence of the model during the training process. TrellisNet-CRF achieves convergence at the 10th iteration step, then the DA-NER and CAN-NER models converge at the 30th epoch, the Bert-BiLSTM-CRF and CNN-CRF models converge at the 40th epoch, and the BiLSTM-CRF model converges at the 50th epoch. We conclude from these results that the TrellisNet-CRF model achieves more stable training and a faster convergence.

**Figure 7:**
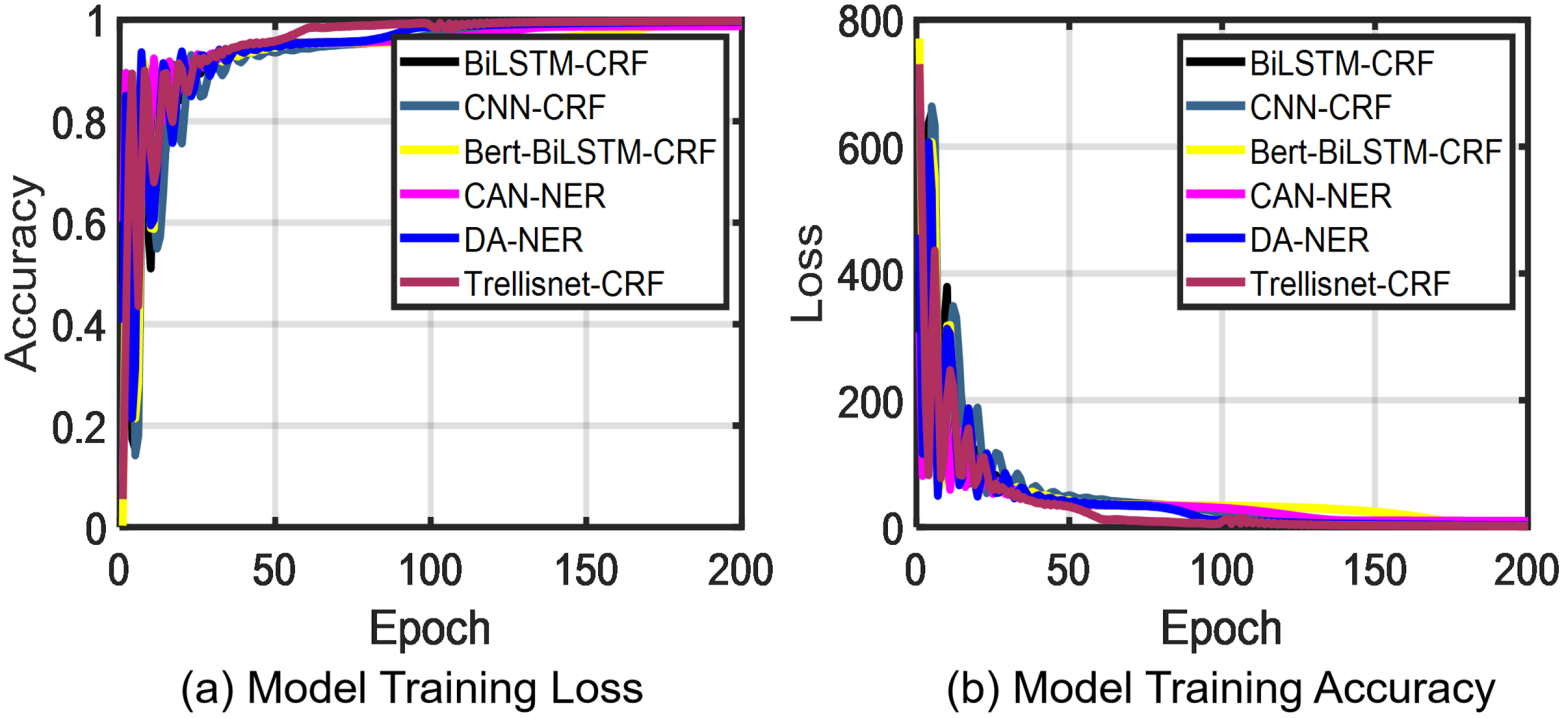
Marine expert c*orpus* test results.

**Figure 8:**
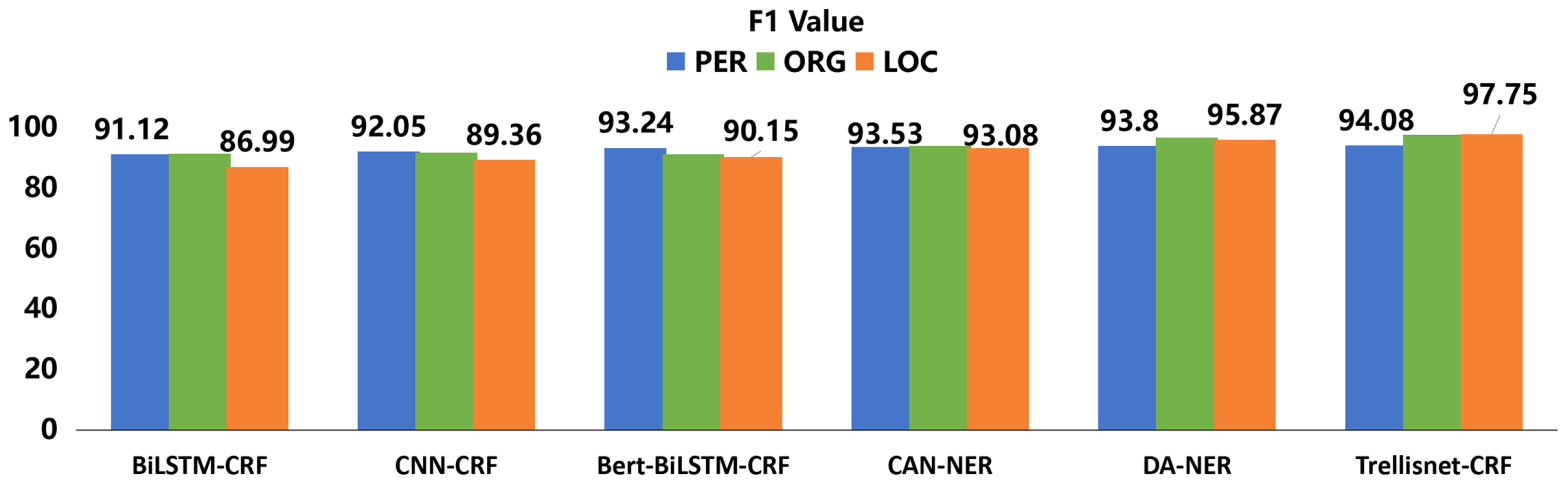
The accuracy of variation LSTM+CRF, variation CNN+CRF, Trellisnet+CRF model.

To verify the effectiveness of the model in recognizing expert entities among the labeled marine field experts, we compared it with five models: BiLSTM-CRF, CNN-CRF, Bert-BiLSTM-CRF, CAN-NER, and DA-NER. As shown in Figs. 7 and 8, the results demonstrated that for the recognition of three entities, our proposed TrellisNet-CRF model has the highest convergence speed and F1 value. The entity recognition network obtained through TrellisNet-CRF network training achieved the best results in both the People’s Daily *corpus* and the marine domain expert *corpus*.

Comparing the results of each method in Table 3, TrellisNet-CRF had the highest accuracy on both the marine domain expert dataset and the People’s Daily dataset, outperforming both the data augmentation algorithm DA-NER based on sequence generative adversarial network (Zhu, Wang & Karlsson, 2019) and the convolutional attention neural network CAN-NER with attention mechanism (Wang, Li & Li, 2020). The results in Table 3 show that the TrellisNet-CRF model is superior to the other five models in the extraction of three named entities: name of person, organization name and location name. The F1 value of the TrellisNet-CRF model reached 96.99% in our study. The TrellisNet-CRF model is also suitable for Chinese named entity recognition tasks indicating that RNN and CNN structures or causal dilated convolutional networks are not the only methods that can be used to extract the dependency between tags in text. In this model, the input sharing mechanism and the extended convolutional network mode were used to optimize the network model to prevent gradient explosion in the training process and to better learn the content meaningful to the target dataset, which is the key to enhancing NER task performance.

**Table 3:** Experiment results.

Model	PER (P%)		ORG (P%)		LOC (P%)	
	Marine	Public	Marine	Public	Marine	Public
BiLSTM-CRF	91.48	90.43	91.21	90.14	86.02	85.97
CNN-CRF	92.72	91.67	92.08	91.01	90.07	89.02
Bert-BiLSTM-CRF	94.01	93.25	92.49	90.42	90.85	89.02
CAN-NER	94.23	94.17	93.39	91.32	91.28	90.23
DA-NER	95.31	94.25	93.99	91.92	92.98	90.93
Trellisnet-CRF	96.99	95.94	94.63	91.56	93.17	91.28

**Figure 9:**
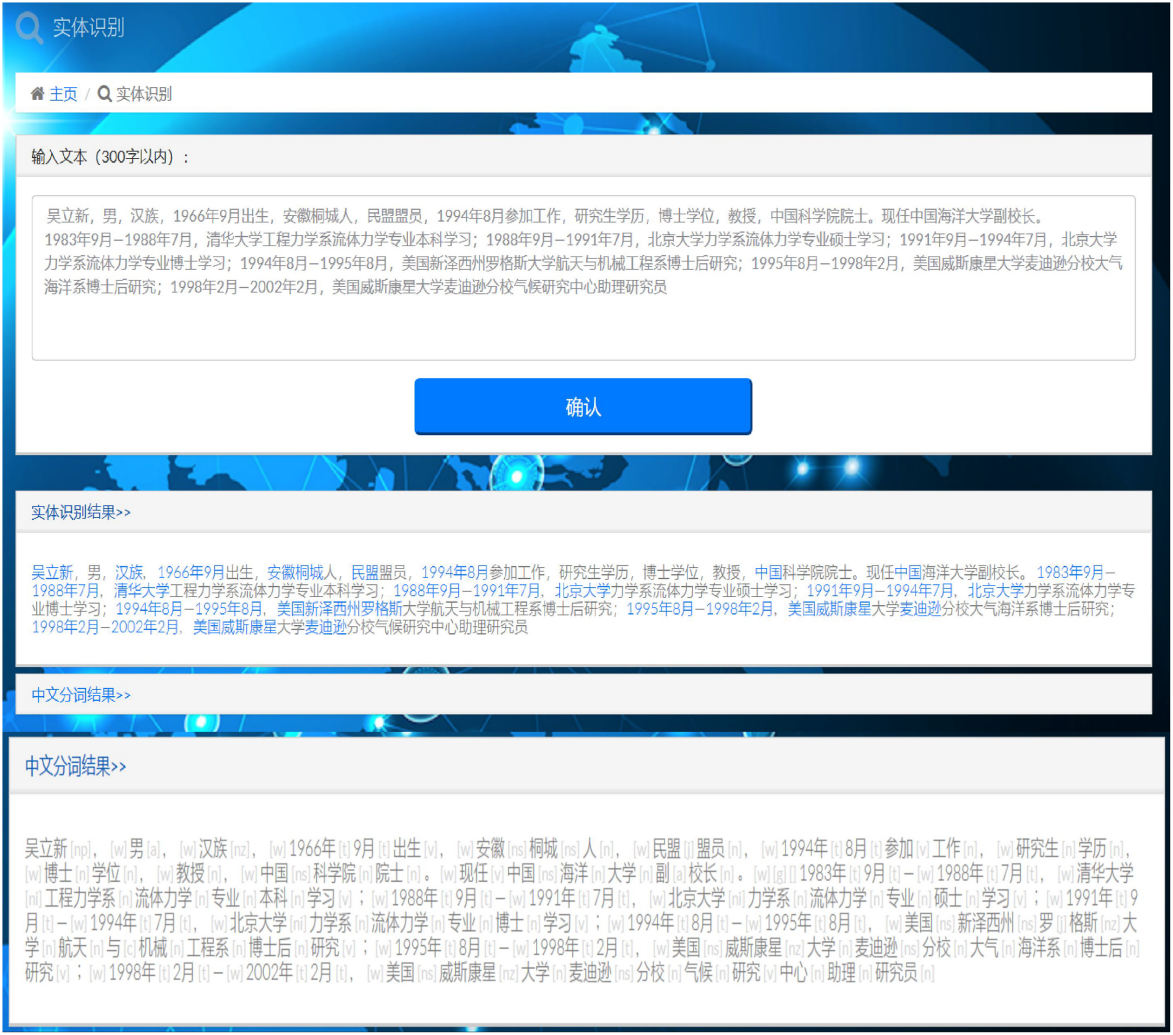
Expert entity identification result.

As illustrated in Fig. 9, we embedded the TrellisNet-CRF model into the entity recognition page of a marine expert management knowledge graph, which was then used to quickly and accurately identify information such as the name and institution of marine experts. The model was accurate and efficient with Chinese named entity recognition.

### Marine field expert knowledge graph visualization

**Figure 10:**
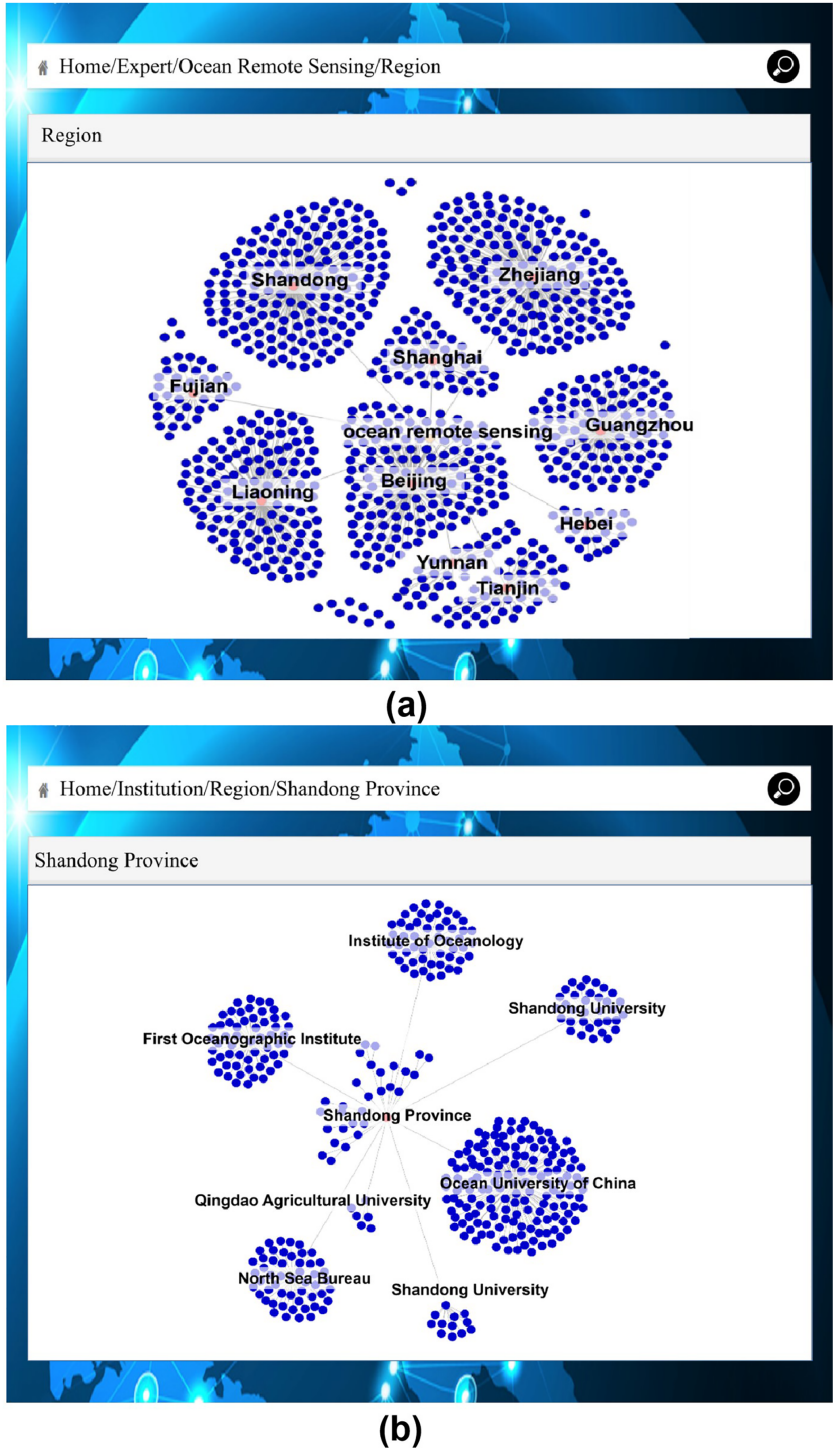
Expert’s research field and institutional relations. (A) Home/Expert/Ocean Remote Sensing/Region. (B) Home/Institution/Region/Shandong Province.

As shown in Fig. 9, if you click the “Expert Identity” button, users can identify Chinese names, place names, organization names and expert identity. If users click on the name of the recognized expert, the publications of the expert, both as a single author or a co-author, are shown. As shown in Fig. 10A, if users want to know more detailed information about the expert’s research field (“ocean remote sensing” in this case), and the provinces and cities where experts in that field are located, these can be found by searching for “ocean remote sensing.” As shown in Fig. 10B, users can quickly find out which institutions in a province or city are engaged in ocean remote sensing, and the names of specific experts at those institutions can be found in the detailed institutional information.

**Figure 11:**
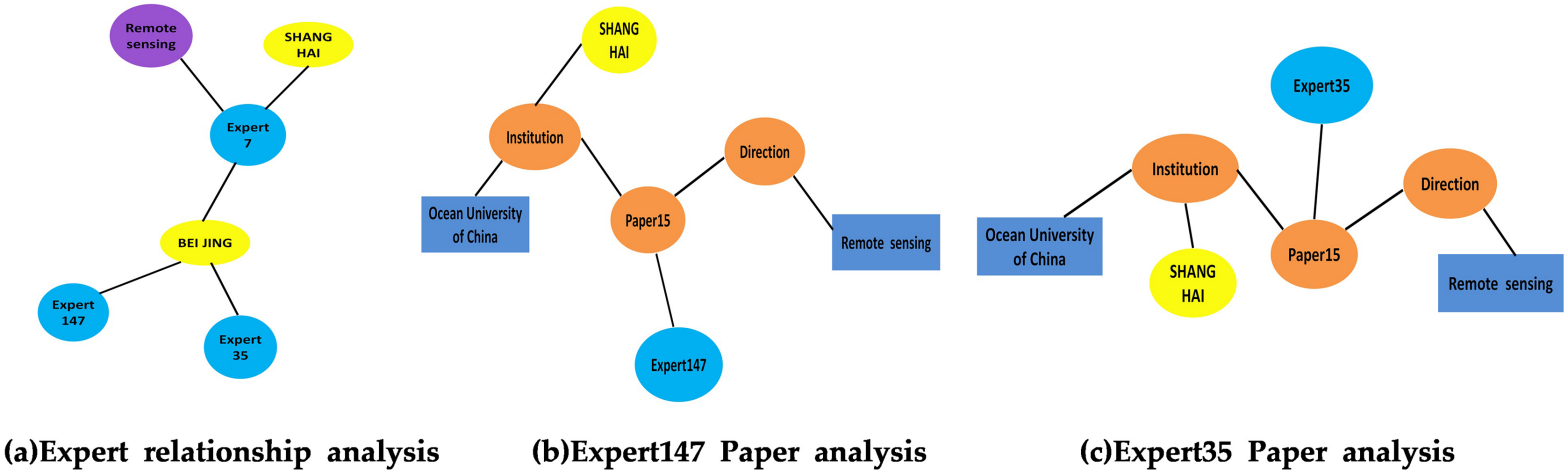
Expert’s paper graph analysis diagram. (A) Expert relationship analysis. (B) Expert147 paper analysis. (C) Expert35 paper analysis.

**Figure 12:**
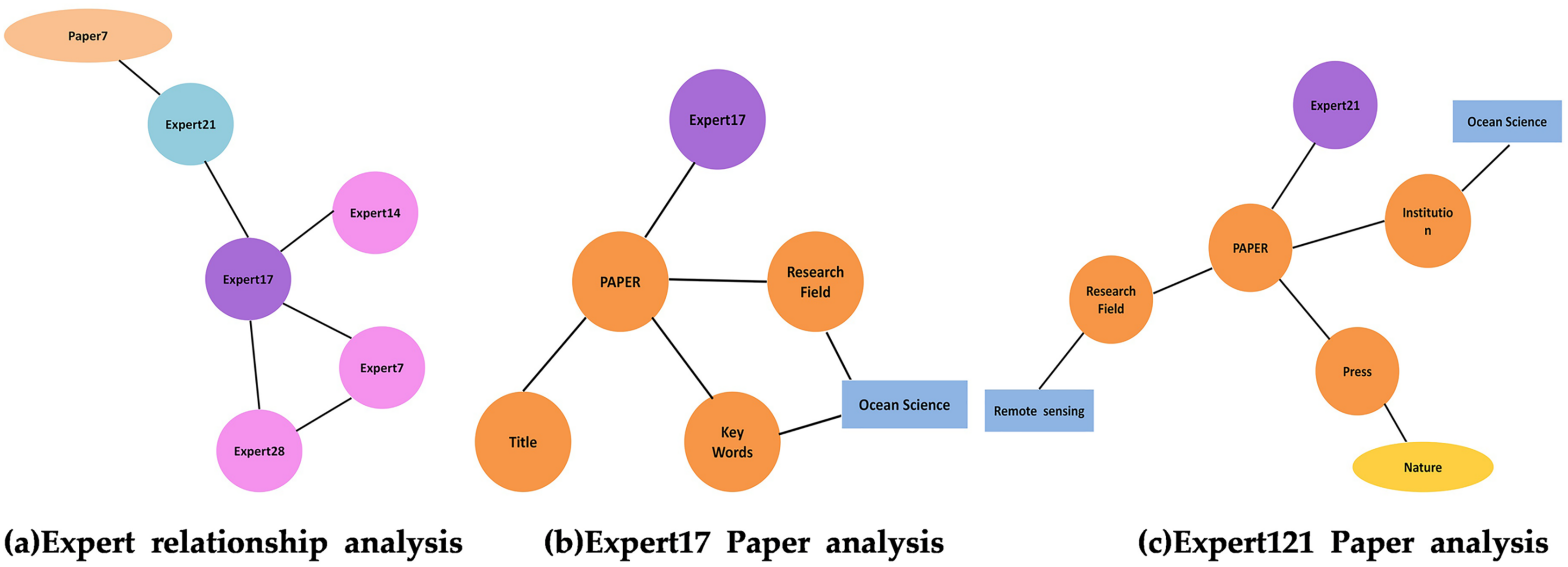
Expert’s key node analysis. (A) Expert relationship analysis. (B) Expert17 paper analysis. (C) Expert121 paper analysis.

**Figure 13:**
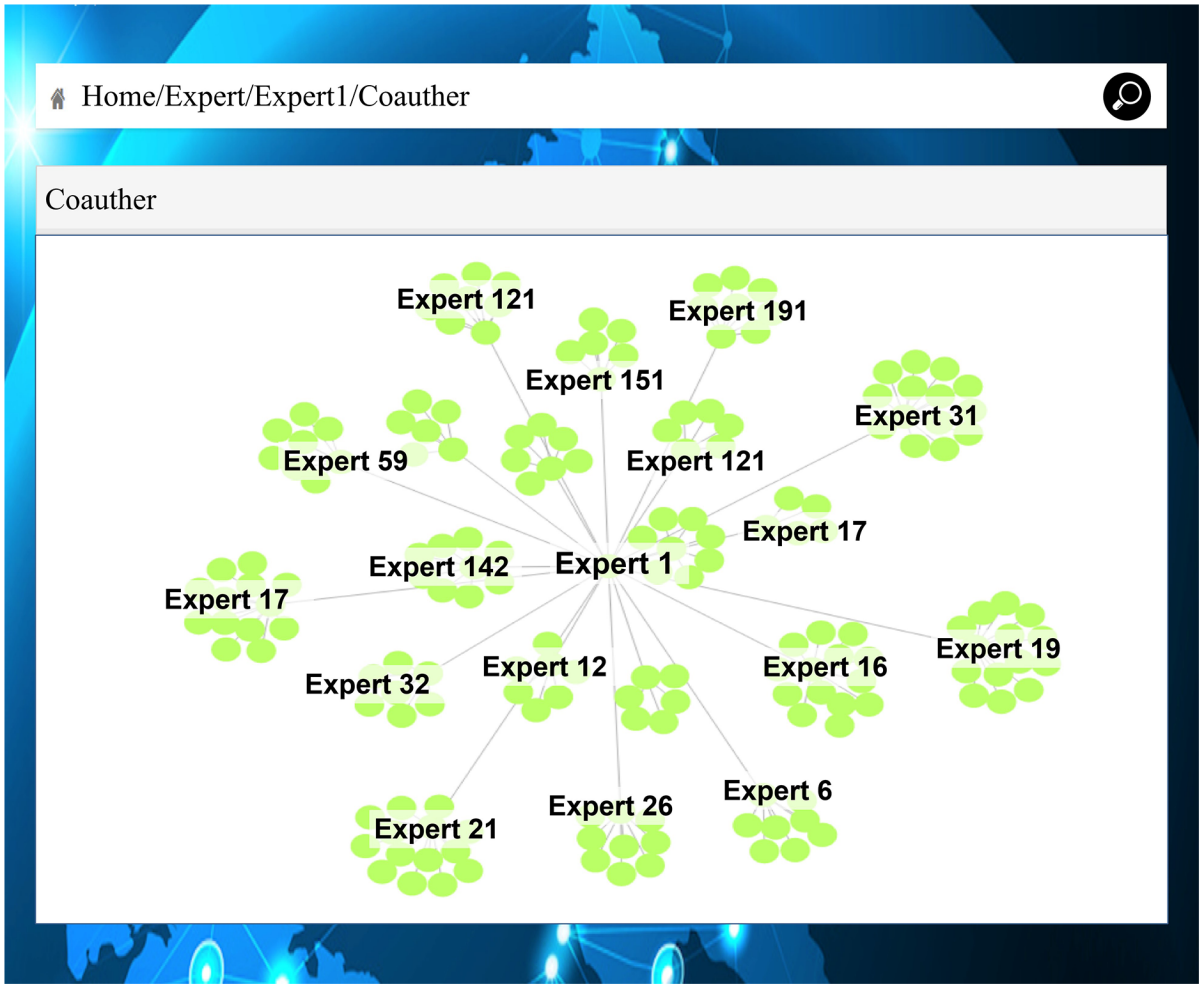
Marine field expert’s cooperation.

As shown in Fig. 11A, both Expert 147 and Expert 35 are in Beijing. Analyzing the literature of Expert 147 and Expert 35, respectively, in Figs. 11B and 11C, shows that they have co-authored a paper and thus share the same research direction. A user can infer that Expert 147 and Expert 35 may be colleagues and have a cooperative scientific relationship. Fig. 12A shows a complete academic circle, in which the keywords and research directions of the papers published by Expert 17 are correlated (Fig. 12B). The research field of the paper published by Expert 21 (Fig. 12C) is remote sensing, and the research institute’s main research direction happens to be marine science. As shown in Fig. 13, the expert cooperation relationship can retrieve the information of authors cooperating with other experts.

The Django framework uses the Python language to easily build most of the content required for a website and further develop a full-featured system (Wu, 2021). Django adopts the MVC mode to facilitate program extension and optimization to facilitate the future reuse of the program. Django is favored by developers for its simplicity, convenient deployment, and low maintenance cost. This study used Django to build a marine field expert knowledge graph. We used the development mode of MVC, which improves the scalability of the system, and the Neo4j database to provide the basis for the distributed storage of knowledge graphs.

## Conclusions and outlook

It is a challenge to take advantage of the tremendous amount of information available on the internet as it is presented in different formats and includes a variety of resources on various topics. A knowledge graph combines big data with knowledge engineering. Knowledge graphs have been applied to a large number of industries, and can also be applied in the field of marine science to organize expert knowledge. The knowledge graph of experts in the marine science domain can integrate fragmented knowledge, reduce the cost of knowledge integration, and realize knowledge integration.

Extracting hidden entities and entity relationships from a large amount of unstructured text is a major technical challenge. To solve this problem, we proposed a method of constructing a knowledge graph of marine domain experts based on the TrellisNet-CRF model. We then evaluated this proposed method by conducting extensive experiments with both the marine expert dataset and the People’s Daily *corpus* dataset. The results of our experiments clearly demonstrated the effectiveness of our proposed solution in extracting hidden entities. Finally, we chose Neo4j, the best-performing graph database, to store the marine expert data. We also used the Django framework to build a marine expert knowledge graph platform for knowledge graph visualization.

A knowledge graph of marine experts was the research object of this study. To improve the accuracy of Chinese named entity recognition, a Trillisnet-CRF model based on dilated convolution was proposed. Using the Trillisnet-CRF model, combined with the semantic model, we constructed a marine science domain expert knowledge graph. In parallel, to improve the accuracy of Chinese named entity recognition, the advanced TrellisNet network model was combined with input injection and a weight sharing mechanism. This solved the problem of the gradient disappearing in the input gate used in the RNN network. The TrellisNet network effectively improved the extraction efficiency of context representation in text through its efficient parallel computing capability. The results of our experiments show that the TrellisNet-CRF network model proposed in this article was quick and accurate in Chinese named entity recognition tasks.

The results show the power of our proposed marine expert management knowledge graph based on TrellisNet-CRF. However, it is worthwhile to point out its limitations. First, the knowledge graph needs to be updated iteratively, and the existing update technology relies too much on manual intervention. Second, knowledge extraction during knowledge graph construction introduces erroneous data that needs to be checked and corrected manually. Future work will focus on how to effectively and automatically update the knowledge graph and improve its construction quality.

## Supplemental Information

10.7717/peerj-cs.1083/supp-1 Supplemental Information 1 Marine expert *Corpus* raw data.

10.7717/peerj-cs.1083/supp-2 Supplemental Information 2 People’s Daily *Corpus* raw data.

10.7717/peerj-cs.1083/supp-3 Supplemental Information 3 Trellisnet-CRF Code.

## Additional Information and Declarations

### Data Availability

The following information was supplied regarding data availability: The source code is available at GitHub: https://github.com/ssupergir/Trellisnet-CRF/tree/master/Code/kgocean. The data is available at GitHub: https://github.com/ssupergir/Trellisnet-CRF/tree/master/data. The Marine expert *Corpus* data and People’s Daily *Corpus* data are available in the Supplemental Files and at Zenodo: Jiajing Wu. (2022). TrellisNet-CRF. Zenodo. DOI 10.5281/zenodo.6526883.
