# Supplementary material for: Constructing marine expert management knowledge graph based on Trellisnet-CRF
Source: PeerJ Comput Sci. 2022 Sep 5;8:e1083. doi: 10.7717/peerj-cs.1083 (PMC9455288; doi:10.7717/peerj-cs.1083)
Supplement: Supplemental Information 2 [file peerj-cs-08-1083-s002.zip › Peopleí»s Daily Corpus-raw data/corpus_Guideline/Guideline_appendix_English.doc]

**Appendix 1 An alphabetical set of markers arranged by the code**

Code names help with the interpretation of the memory

Ag form morpheme adjective sex morpheme.The adjective code is a and the morpheme code g is precby A.

The a adjective takes the first letter of the English adjective adjective.

The A d adverph is the direct adjective of the adverbial.The adjective code a and the adverb code d are all together.

An adjective whose an nominal form has a noun function.The adjective code a and the noun code n are all together.

Bg

The b distinguishing word is the voice mother of the Chinese character "goodbye".

The c-conjunction takes the first letter of the English conjunction, conjunction.

Dg adverb sex morpheme.The adverb code is d and the morpheme code g is preceded by a D.

The d adverb takes the second letter of adverb because its first letter has been used for the adjective.

The e lament is the first letter of the English lament exclamation.

The f-bearing word takes the voice mother of the Chinese character "square".

The vast majority of g morphemes can be used as the "root" of synthisms, taking the initials of the Chinese character "root".A g was never used for its subclass.

Before h, take the first letter of English head.

The i idiom takes the first letter of the English idiom idiom.

The short is the voice of the Chinese character "Jane".

k After the addition of components

The idiom has not yet become an idiom, a little "temporary", take "near" voice mother.

Mg

The m word takes the third letter of English numeral, n, u has his use.

Ng name morphemes nominal morpheme.The noun code is n and the morpheme code g is precby N.

The n noun takes the first letter of the English noun noun.

The nr noun code, n, and the vocal mother of the person (ren), are all together.

The ns place name noun code n and the place word code s are all together.

The nt organization group "group" is t, noun code n and t together.

nx

The first letter of the other special name "special" is z, and the noun code n and z are all together.

The o onometic takes the first letter of the English onometic onomatopoeia.

The p preposition takes the first letter of the English preposition prepositional.

Qg

The q-quantifier takes the first letter of the English quantity.

Rg

The r pronoun takes the second letter of the English pronoun pronoun, since p has been used for prepositions.

The s place word takes the 1st letter of the English space.

Lingeme Time word sex morpheme at Tg.The time word code is t, set with T before the code g of the morpheme.

The t time word takes the 1st letter of the English time.

Ug

The u aid takes the second letter of the English aid auxiliary, as a has been used for adjectives.

Vg dynamic morpheme verb sex morpheme.The verb code is the code v.Place a V ahead of the code g of the morpheme.

22

The v-verb takes the first letter of the English verb, the verb.

The vd adverdirect adverbial verb.The code of verbs and adverbs are together.

A vn name verb refers to a verb with a noun function.The verb and noun code are together.

w punctuation

Non-morpheme word x The non-morpheme word is just a symbol, and the letter x is usually used to represent unknown numbers, symbols.

Yg

The y-tone word takes the voice mother of the Chinese character "language".

The z-state word takes the first letter of the voice mother of the Chinese character "shape".

Note: In the first six months of the People's Daily, the following markers also appear: Bg (distinguishing morpheme), Mg (number morpheme), Qg (quantitative morpheme), Rg (generation morpheme), Ug (auxiliary morpheme), and Yg (tone morpheme).Whether the emergence of these morphemes is reasonable needs to be considered.Since they appear very rarely, they do not affect the use of the corpus.The Institute of Computational Linguistics of Peking University will study it as soon as possible and give a reasonable conclusion.These markers are not excluded in Appendix 2.**Appendix 2 A set of markers arranged in Chinese pinyin order by name**

23

Name and code are written in Pinyin

Punbration w biao1dian3fu2hao4

Idiom: i cheng2yu3

Place word s chu4suo3ci2

The pronouns, r dai4ci2

Place name, ns di4ming2

The Verb, v dong4ci2

Momorpheme Vg dong4yu3su4

imuword f fang1wei4ci2

Non-morpheme word x fei1yu3su4zi4

Ab d fu4ci2

The Adverb vd fu4dong4ci2

Acronym ad fu4xing2ci2

The adverxeme, Dg fu4yu3su4

Resequent component k hou4jie1cheng2fen4

Institutional group nt ji1gou4tuan2ti3

Short for short j jian3cheng1lve4yu3

The Preposition, p jie4ci2

Ligue c lian2ci2

The Quantifier q liang4ci2

Noun, n ming2ci2

Name-verb vn ming2dong4ci2

Name word an ming2xing2ci2

The first name morpheme, Ng ming2yu3su4

Sononometic o ni2sheng1ci2

Other special name: nz qi2ta1zhuan1ming2

Front bonding component h qian2jie1cheng2fen4

Differential word b qu1bie2ci2

Person name nr ren2ming2

Time word t shi2jian1ci2

Time-morpheme Tg shi2yu3su4

Count word m shu4ci2

Lament words e tan4ci2

idiom l xi1yong4yu3

The adjective, a xing2rong2ci2

Gmorpheme Ag xing2yu3su4

Tone word y yu3qi4ci2

Pheme g yu3su4

Helps: u zhu4ci2

State word z zhuang4tai4ci2

24

**Appendix 3 references**

1 Chinese national standard GB13715, Modern Chinese Classification Specification for Information Processing, see Modern Information Processing by Liu Yuan et al

Chinese Classification Specification and automatic Classification Methods, Beijing: Tsinghua University Press, 1st edition, 1994.

1. Lu Zhiwei et al., construction of Chinese, Science Press, 1964.
2. Yu Shiwen, Zhu Xuefeng, Wang Hui, The New Progress in the Modern Chinese Grammar Information Dictionary, Journal of Chinese Information, 2001,

Volume 15, Issue 1,59-65 (Seal 3)

1. Duan Huiming, Matai Kusui, Xu Guowei, Hu Guoxin, Yu Shiwen, the production and use of large-scale Chinese annotated corpus,

Language Application, Issue 2,2000,72-77

1. Yu Shiwen, Zhu Xuefeng, Duan Huiming, the processing specifications for the large-scale modern Chinese annotated corpus, The Chinese Journal of Informatics,

Issue 6,2000,58-64

1. Yu Shiwen, Zhu Xuefeng, Wang Hui, Zhang Yunyun, The Modern Chinese Grammar Information Dictionary, Beijing: Tsinghua University Press

The 1st Edition, 1998.

7 Zhu Xuefeng, Yu Shiwen, Wang Hui, The Practice of classifying 50,000 Words in Modern Chinese, The Application of Language, No.4,1997,

88—94

8 Zhou Qiang, Yu Shiwen, a multi-level processing method of Chinese corpus that combines word cutting and word annotation,

See: Chen Li, Computer Research and Application, Beijing Institute of Language and Languages Press, 126- -131.1993

9 Zhou Qiang, Duan Huiming, word cutting and word annotation processing in modern Chinese corpus processing, China Computer News,

On May 31,1994, the 85th Edition.

10 Zhou Qiang, Zhang Wei, Yu Shiwen, Construction of Shuku, Chinese Informatics Journal, No.4,1997,42- -51

Zhu Dexi, "Modern Chinese Grammar Research", Beijing: The Commercial Press, 1980

25
