# Supplementary material for: Constructing marine expert management knowledge graph based on Trellisnet-CRF
Source: PeerJ Comput Sci. 2022 Sep 5;8:e1083. doi: 10.7717/peerj-cs.1083 (PMC9455288; doi:10.7717/peerj-cs.1083)
Supplement: Supplemental Information 2 [file peerj-cs-08-1083-s002.zip › Peopleí»s Daily Corpus-raw data/corpus_Guideline/Guideline_contents_English.doc]

catalogue

Modern Chinese corpus processing specifications, —— word segmentation and word marking

⒈ Preface …………………………………………………………1

⒉ tangent specification ……………………………………………………3

.12 Basic concept of …………………………………………………3

2.2 Supplement and adjustment of the word segmentation specification………………………………… 4

Canonical ………………………………… combining ⒊ tangection and annotation 12

.13 Overlap………………………………………………………12

3.2 Additional………………………………………………………14

3.3, compound words……………………………………………………15

The ⒋ dimension specification is the …………………………………………………… 16

4.1 Relationship between word annotation and grammar information dictionary……………………………16

4.2 Type form choice of common multi-class words……………………………………16

4.3 About tagging vn, vd, an, ad…………………………………18

⒌ Postscript ………………………………………………………… 21

appendix:

⒈ tag set ………………………… arranged in alphabetical order by code22

⒉ A marker set …………………… arranged in the Chinese pinyin order of the name24

⒊ reference ……………………………………………………25
