# Supplementary material for: Constructing marine expert management knowledge graph based on Trellisnet-CRF
Source: PeerJ Comput Sci. 2022 Sep 5;8:e1083. doi: 10.7717/peerj-cs.1083 (PMC9455288; doi:10.7717/peerj-cs.1083)
Supplement: Supplemental Information 2 [file peerj-cs-08-1083-s002.zip › Peopleí»s Daily Corpus-raw data/corpus_Guideline/Guideline_English.doc]

Modern Chinese language corpus processing specifications

—— word segmentation and word sex annotation

**⒈ preface**

The Institute of Computational Linguistics of Peking University has been conducting multi-level processing research of Chinese corpus since 1992.The first step is the segmentation and verbal annotation of the original corpus.In 1994, the Code for Modern Chinese Text Classification and Word Sex Classification V 1.0 was formulated.In the past few years, we have completed the segmentation and annotation of about 600,000 words of corpora, and explored in the direction of automatic phrase identification and tree library construction.After accumulating long-term practical experience, I recently conducted the People's Daily corpus processing experiment.In order to ensure the smooth progress of the important language project of large-scale corpus processing, the Institute of Computational Linguistics of Peking University formulated the Code V 2.0 for Modern Chinese Text Classification and Classification of Words (draft) in October 1998.Because the task of this processing is beyond the scope of word segmentation and nature of speech annotation, but the center of gravity remains unchanged, in order to facilitate the future development (formulate the series of specifications), the new version of the specification was renamed "Modern Chinese Cora Processing Specification —— word segmentation and word annotation (March 1999 edition)".Now, the Institute of Computational Linguistics of Peking University has completed the processing task of the corpus for the first 8 months of the People's Daily in 1998, and delivered the Fujitsu for acceptance and use.Through more than two years of practice, the "standard" has some new understanding.Some markers were also added.The omissions in the Code will now be revised and supplemented to form the July 2001 edition.In the processed corpus (hereinafter referred to as "annotated corpus") in 6 months before August 2001, modern Chinese Corpus Processing Specification —— Word segmentation and Word annotation (July 2001 edition) is an important part of the data.

The basic ideas for formulating the Processing Specification of Modern Chinese corpora are as follows:

1. The segmentation specification of words is as consistent as possible with the Chinese national standard GB13715 "Modern Chinese segmentation specification for Information Processing" (hereinafter referred to as "segmentation specification").Because now word segmentation and word annotation is combined, and has a modern Chinese grammar information dictionary (sometimes referred to as "grammar information dictionary" or "grammar dictionary") can be used as the basic reference of word segmentation and word annotation, it is necessary to "segmentation specification" for the necessary adjustment and supplement.
2. Small tag set.In addition to the 26 words in the modern Chinese grammar information dictionary (noun n, time t, place s, orientation f, number, m, quantifier q, distinguishing word b, pronoun r, verb, verb v, adjective a, state z, adverb d, preposition p, conjunction c, conjunction u, tone y, lament e, harmonic o, idiom i, idiom l, j, h, substance k, morpheme g, non-element x , punctuation w), The following 3 categories of markers were added: classification markers for ① proper nouns, By the human name nr, place name ns, Name of organization unit nt, Other proper nouns nz, Letters or strings in other foreign languages like English nx.The subclass tag of ② morpheme, i. e. Ng, morpheme Vg, description morpheme Ag, adverxeme Dg, since the actual use of subclass tags, the morpheme tag g is not present in the annotation corpus.In the first six months, there were very few morphemes in the annotated corpus of other subcategories, which are named in the same way. The quantitative morpheme is to change the quantifier code q to capital Q, and then add a g, namely Qg.The subclass markers of ③ verbs and adjectives, namely name verb vn (verb with noun characteristics), name form an (adjective with noun characteristics), adververb vd (verb with adverb characteristics), and adververb ad (adjective with adverb characteristics).About 40 total.Combined with the Modern Chinese Grammar Information Dictionary, this marker set is easily expanded.
3. The versity of norms.Both to meet the needs of language information processing and corpus linguistics research, and to provide sufficient material for traditional language research, suitable for both automatic computer processing and manual proofreading.
4. The guiding role of phrase (phrase) standard Chinese grammar system.There is no simple one-to-one correspondence between Chinese word classes and syntactic elements.The same syntactic component can be filled by words with different parts of words; the same word with deterministic nature can act as different syntactic elements without any different marks.Modern Chinese Grammar Information Dictionary is developed under the guidance of phrase standard grammar system. Its most basic result is that the distribution of tens of thousands of words according to their grammar functions in actual corpora, especially the dominant function distribution determines their nature of words (i. e., the word category to which they belong).When marking the nature of words, we should make full use of the Modern Chinese Grammar Information Dictionary to undertake this basic result, and avoid determining the nature of words only according to the sentence composition in the sentence.At the same time, considering the different opinions in the division of Chinese words, the nominal verb vn, nominal verb an, adververb vd, adververb ad are added in the marker set.Of course, adding these markers is not just a buffer against disagreement, mainly to provide a measurement basis for the concurrent study of words, and to prepare for the probabilistic grammatical properties of Chinese words.

⑸ in order to study a large number of proper nouns (place names, group names, etc.) (from naming rules to automatic recognition), on the basis of segmentation and annotation of phrase proper nouns with brackets and type markers (mainly nt, nz, and a small number of ns).

The ⑹ does not use this specification in isolation.When using this specification, it will be closely coordinated with the research results of the Modern Chinese Grammar Information Dictionary.When the current research is connected with the future applications (Chinese information retrieval, Chinese information extraction, Chinese and foreign machine translation, etc.), we can easily use the rich word grammatical attribute information in the dictionary.The annotation corpus can verify and enrich the attributes of words in the dictionary, and then establish the probabilistic grammatical attribute description of words.The label corpus is combined with the Modern Chinese Grammar Information Dictionary to form a three-dimensional language knowledge base.The further deep processing of the corpus (tree library, phonetic annotation, semantic annotation, etc.) also has a reliable basis.

⑺ HPC works.Based on this specification, continue to expand the function of automatic machining software and improve its accuracy.Manual proofreading is necessary.It is necessary to pay attention to the discussion between proofreading personnel, and to unify the different opinions on academic issues into the formulation and implementation of norms.Develop machine-assisted editing and corpus management software to ensure the consistency of the corpus processing.

This specification is divided into three parts:

① tangent specification

The specification mainly stipulates the principle of word segmentation in modern Chinese, that is, what kind of Chinese character combination can be a segmentation unit.Chapter 2 introduces the supplement and adjustment of the "dividing word specification" in detail.

Specification for combining ② bisection and annotation

In Chinese, words like "monosyllabic verbs + monosyllabic nouns" usually constitute new nouns, for which they should be treated as a partitioning unit, even if they are not logged in the dictionary.Therefore, in this specification, some word construction rules based on the nature of words description are given, stipulating what combinations can be treated as a tangential unit, and giving the part-word markers of the new combination of words.This section is mainly concentrated in Chapter 3.

1. Mark the specification

③ -1 General nature of the word annotation

The annotation specification is used to determine the labeling of the ent units.Using the 70,000 words list provided by the Grammar Dictionary Group in July 1999 as the basic reference, its meanings include:

1. The marker set is based on 26 word class markers, and name verbs, adververbs, nominal words, adverbs and proper nouns are added by a lower case letter after the verb code v, adjective code a, noun code n, and the morpheme mark is a capital letter in front of the morpheme code g.
2. If a word already belongs to one or several words in a grammar dictionary, it does not easily increase the nature of words when marking.For example, "training" and "emphasis" are only verbs in the grammar dictionary, do not change them to nouns or adverbs only based on their function in the current sentence, which can be marked as name verb vn or adververb vd.
3. When the grammar dictionary gives a word to determine the wrong or incomplete nature of words, of course, it should also be revised or supplemented.The dictionary team should then be notified.
4. Even if the abbreviation in the grammar dictionary actually refers to the group, organization, organization name, or place name, it is still marked with j, but not changed to nt or ns.
5. Although the historical dynasty names of "Tang Dynasty" and "Song Dynasty" are also special names, the grammar dictionary has been used as time words, which is still marked with t, not changed to nz.

Chapter 4 focuses on the specification of annotation.

③ -2 proper noun notation

Here, the meaning of a "proper noun" is expanded.Phrase-type place names, group names, and other proprietary names are enclosed in ASCII code square brackets on the basis of word segmentation, and marked by the corresponding ns, nt, nz, and the square brackets are not nested.Both chapters 2 and 3 cover this part.

**⒉ section specification**

**2.1 Basic concepts**

⑴ is divided into units

"Classification unit" is a basic concept in the "classification standard".It refers to the basic unit used in information processing and has certain semantic and grammar functions in information processing.In order to connect with the "word specification", the concept of "word unit" is still used here, but the term is changed to "word unit", because the term "word unit" has been used in English grammar for a long time and is familiar, and to express multiple concepts of the same or adjacent discipline with the same term is easy to cause confusion.

According to the definition and interpretation of the "dividing unit" in the "segmentation specification", the "dividing unit" in this segmentation specification is mainly the word, which also includes a part of the closely combined and used stable phrases.Isolated morpheme or nonmorpheme words in some special cases may also appear in tangential sequences, such as in the clutch form of the verb

Out / v / u one / m times / q difference / Ng./w

In, "difference / Ng" is a morpheme; as in

Partridge / n / u par/ x / v what / r meaning / n / y?/w

In, "par/ x" is non-morph.

**Considering the number of words, the combination of two words is widely regarded as a syncopation unit, three words are more strict, more than four words if not idioms, idioms are generally not regarded as a syncopation unit.**

⑵ dictionary entry

"Dictionary entry" (or "entry") refers to those words included in the Modern Chinese Grammar Information Dictionary.The total number of entries provided to the project in July 1999 is about 73,000, which have been classified with word marks.

⑶ split the relationship between units and entries

In Chinese, the boundary between words and phrases, into words and no words is ambiguous.This specification stipulates that all entries (including words, phrases, idioms, idioms, abbreviation and even punctuation marks, etc.) are generally segmented units.Because there are up to 73,000 entries in the grammar dictionary, the coverage of the real text is very high, so that the vast majority of tangential units and the grammar dictionary entries are consistent, but there are still differences between the two.For example, idioms and idioms with more than five words are divided into units, but they are not included in the current grammar dictionary.Digital words and time words like "one hundred-twenty-eight", "three-fifths", "nine percent", "1998" and "October 30" are actually infinite, and the grammar dictionary can not receive all, only a small amount of components.In turn, words like "fraction" and "percent" are earned as auxiliary words, but they are not tangential units.The anterior components, posterior components, morphemes, nonmorpheme words contained in the grammar dictionary are not tangent units, although they also appear in isolation when they cannot be combined with the anterior and posterior components.

When dealing with large scale real text, unlogged words.Chapter 3 gives some rules of the construction of synthisms.Partient units, automatically generated under these rules or confirmed by proofreaders, may be added in the grammar dictionary.

**2.2 Supplement and adjustment of the word segmentation specification**

For prominence, the following are marked with the symbol "*" and "".

⑴ person name: nr

① The family names and names of non-Han people in the same way as Han people were divided separately and marked as nr.

Zhang / nr Renwei / nr, Ouyang / nr Xiu / nr, Nguyen / nr Zhixiong / nr, Park / nr Zhenai / nr

* In addition to the single surname and multiple surname, Han people also have two surnames, that is, after some women get married, they add zhang to their original surname

Husband's surname.Such as: Chen Fang Ansheng.This situation is divided and marked as: Chen / nr Fang / nr Ansheng / nr;

Tang Jiang, cut into, marked as: Tang / nr Jiang / nr.

② title, title or address should be separated.

Jiang / nr Chairman / n, Xiaoping / nr Comrade / n, Jiang / nr General Secretary / n,

Professor Zhang / nr / n, Minister Wang / nr / n, Manager Chen / nr / n,

Aunt Li / nr / n, Aunt Liu / nr / n, Aunt Long / nr / n

③ if the abbreviation, respectful name and so on of people for two words, it is combined into a partitioning unit, and marked with nr.

Lao Zhang / nr, Da Li / nr, Xiao Hao / nr, Guo Lao / nr, and Chen Zong / nr

④ obviously with the ranking of the relative appellation should be cut apart, if it is not clearly divided is not cut open.

Three / m brother / n, Aunt / n, Big / a daughter / n, Big brother / n, Little brother / n, Dad / n

* ⑤ The pseudonyms of the or poorly distinguished surnames and given names of some famous authors usually act as a syncopation unit.

Lu Xun / nr, MAO Dun / nr, Ba Jin / nr, Sanmao / nr, Qiong Yao / nr, White Birch / nr

⑥ translations of foreigners or ethnic minorities (including those of Japanese people) shall not be divided and marked as nr.

Clinton / nr, Yeltsin / nr, Cholma / nr, Kobayashi / nr, Beijing Studies / nr,

Washington / nr, and Albert Einstein / nr

Some Westerners have small dots in their names and are not separate.

Karl Marx / nr

⑵ Place name: ns

Anhui / ns, Shenzhen / ns, Hangzhou / ns, Lhasa / ns, Harbin / ns, Hohhot / ns,

Urumqi / ns, Yangtze River / ns, Yellow Sea / ns, Pacific / ns, Mount Tai / ns, Mount Hua / ns,

Asia / ns, Hainan Island / ns, Taihu Lake / ns, Baiyangdian / ns, Russia / ns, Kazakhstan / ns,

Petersburg / ns, Volgograd / ns

The ① country name, regardless of the length, serves as a division unit.

China / ns, People's Republic of China / ns, Japan / ns, United States of America / ns, United States / ns

△ ② place names have "province", "city", "county", "district", "township", "town", "village", "flag", "state", "capital", "government" government "," road " and other characters, not separated, as a division unit.

Sichuan Province / ns, Tianjin / ns, Jingdezhen City / ns, Shashi City / ns, Mudanjiang City / ns, Zhengding County / ns, Haidian District / ns, Tongzhou District / ns, Dongsheng Township / ns, Shuangqiao Town / ns Nanhua Village / ns, Washington State / ns, Ohio / ns, Tokyo / ns, Osaka Prefecture / ns, Hokkaido / ns, Nagano / ns, Kaifeng / ns, Xuancheng County / ns

The administrative division after the △ ③ place name has more than two Chinese characters, so the place name should be divided with the name of the administrative division, but the place name with the name of the administrative division should be enclosed in square brackets and marked with ns.

[Wuhu / ns Zone / n] ns, [Xuancheng / ns Region / n] ns, [Inner Mongolia / ns Autonomous Region / n] ns,

[Ningxia / ns Hui / nz Autonomous Region / n] ns, [Shenzhen / ns Special Economic Zone / n] ns,

[Xiamen / ns economy / n Special Zone / n] ns, [Hong Kong / ns Special / a Administrative Region / n] ns,

[Hong Kong / ns SAR / n] ns, [Washington / ns DC / n] ns,

[Guangxi / ns Huanjiang / ns Maonan / nz Autonomous County / n] ns,

[Qinghai / ns Guoluo / ns Tibetan / nz Autonomous Prefecture / n] ns

④ place names after a word of landform, such as "river, river, mountain, ocean, ocean, sea, island, peak, lake", etc., will not be divided.

Yalu River / ns, Amazon / ns, Himalayas / ns, Everest / ns, Mediterranean / ns, Atlantic / ns, Dongting Lake / ns, Sephorus Island / ns

△⑤ common noun after the landform of more than two Chinese characters, should be cut.Cover place names with the common noun and mark ns.

[Taiwan / ns Strait / n] ns, [North China / ns Plain / n] ns, [Pamir / ns Plateau / n] ns, [Nansha / ns Islands / n] ns, [Jingdong / ns Big / a Canyon / n] ns [Hengduan / b Mountains / n] ns

After the place name ⑥ there is a common noun indicating a word of natural division, such as "street, road, road, lane, li, town, town, village, village, lane, fort", etc., will not be divided.

Zhongguancun / ns, Chang'an Avenue / ns, Xueyuan Road / ns, Jingdezhen / ns, Wujiapu / ns,

Panggezhuang / ns, Sanyuanli / ns, Petersburg / ns, North Vegetable Market Lane / ns,

△⑦ after the place name of the natural division if there are more than two Chinese characters, should be cut.Place names should also be enclosed with natural zoning nouns in square brackets and marked with ns.

[Rice / ns Street / n] ns, [Jiang / nz Hutong / n] ns, [Ting / ns Park / n] ns

⑧ size place names are marked as follows:

Beijing / ns Haidian District / ns Haidian Town / ns [South / f Street / n] ns [Jiangjia / nz Hutong / n] ns 24 / m / q

△ (3) Exclusive name of the group, organization and organization: nt

The proprietary name of the ① group, organization or organization is logged in the grammar dictionary as nt.

United Nations / nt, CPC Central Committee / nt, State Council / nt, Peking University / nt

② The proprietary names of most groups, organizations and organizations are generally phrase, long, and contain place names or personal names, which will not be logged in in the grammar dictionary, this specification stipulates that they are divided first, and then combined, and square brackets are marked as nt.

[China / ns Computer / n Society / n] nt, [Hong Kong / ns Watch Industry / n Association / n] nt,

[Yantai / ns University / n] nt, [Hefei / ns Normal / n College / n] nt,

[Beijing / ns Library / n] nt, [Fujitsu / nz Corporation / n] nt,

[Fragrant Hill / ns Botanical Garden / n] nt, [Anna / nz Beauty Salon / n] nt,

[Shanghai / ns Watch / n Factory / n] nt, [Yonghe / nz bakery / n] nt,

[Beijing / ns Guoan / nz team / n] nt, Beijing team / nt, Lei Feng class / nt

Note: "Beijing team", "Lei Feng class" as "cutting units", "factory" and "watch" cut, and "shop" with "pancake" and together.These treatments comply with the general combination of monosyllabic nouns with subsequent monosyllabic nouns or morphemes.See Chapter 4 for more details.

The specific reference of ③ group, organization, organization name is necessary, isolated "university, college, library, botanical garden," etc. are only labeled n, not nt.At the beginning of an article, the specific meaning of the name of the organization, organization is clear, and the latter often uses abbreviation.When the special name is omitted and only common nouns remain, nt.If the interview with the secretary of Zhejiang Provincial Party Committee, the reporter will definitely write "Zhejiang Provincial Party Committee", then processed into:

[Zhejiang / ns Provincial Party Committee / n] nt

When the secretary of the Provincial Party Committee is quoted later, although "Provincial Party Committee" refers to "Zhejiang Provincial Party Committee", it is only marked as:

provincial Party committee /n

That is to words, this processing only considers the local context, not remote related semantic analysis.Similarly, the "President's Office of Peking University" should be processed as:

[Peking University / nt President / n Office / n] nt

If there is only the "president's office" in the sentence and no "Peking University" in front, it is only processed into:

Principal / n Office / n

Although in a given larger context, the "principal's office" is specifically meant.

Despite the ③ regulations, the name of the only group, organization, organization known in international or China, even if there is no special name, is nt.

UN / nt, [World / n Trade / n Organization / n] nt,

State Department / nt, Ministry of Foreign Affairs / nt, Ministry of Finance / nt, Ministry of Education / nt, Ministry of Defense / nt,

[National / n Education / vn Council / n] nt, [Information / n Industry / n Ministry / n] nt,

[National / n Information / n Technology / n Standardization / vn Committee / n] nt,

[National / n General / b Union / n] nt, [National / n People / n representative / n Congress / n] nt

The "State Department" of the United States, the "Ministry of Foreign Affairs, the Treasury Department, and the Ministry of Education" of other countries, must appear after the name of its host country before being jointly labeled as nt.

[US / ns State Department / n] nt, [France / ns Foreign Office / n] nt, [US / j Congress / n] nt

Some government agencies in Japan have special names, either "Japan" or nt.

[Japan / ns Ministry of Foreign Affairs / nt] nt, [Japan / j Ministry of Trade / nt] nt, [Japan / ns Ministry of Justice / nt] nt,

General Province, / nt

The name of ⑤ is handled as follows:

[United Nations / nt UNESCO / j Organization / n] nt

[China / ns Bank / n Beijing / ns Branch / n] nt

[Hebei Province / ns Zhengding County / ns Xi Pingle Township / ns Nanhua Village / ns Party branch / n] nt

[Peking University / nt Changping / ns / n] nt

[Anhui / ns National People's Congress / j Standing Committee / j Office / n] nt

[Peking University / nt Computing / vn Linguistics / n Institute / n] nt

If the current name contains special names (such as "Beijing / ns Branch / n", "Nanhua Village / ns Party branch / n" and "Changping / ns branch / n"), it can also be marked separately marked as nt from the previous upper name.

[China / ns Bank / n] nt [Beijing / ns Branch / n] nt

Hebei Province / ns Zhengding County / ns Xiping Le Township / ns [Nanhua Village / ns Party branch / n] nt

Peking University / nt [Changping / ns Branch / n] nt

If the lower name does not contain a specific name, it must be bundled with the upper name.

Example of treatment when adding parentheses in ⑥ group, organization, organization names.

[Baoshan / ns Steel / n (/ w Baosteel / j) / w General / b Company / n] nt

[Baoshan / ns Steel / n General / b Company / n] nt (/ w Baosteel / j) / w

△ (4) Other than person name, country name, place name, group, organization, organization are marked with nz, specified as follows.

The morphemes of monosyllables after the ① proprietary name, such as "family" for nation, "language" for language, and "text" for text, are not divided and marked as nz.

Manchu / nz, Russian / nz, Kazak / nz, Serbian / nz, Alpine / nz,

Uyghur / nz, Mongolian / nz, Chinese / nz, Romani / nz, Czech / nz

Chinese / nz, English / nz, Spanish / nz, Mongolian / nz, Russian / nz

Monosyllabic nouns, such as the human "person" or the award "award", are usually not divided as nz; also allowed to be marked separately.

Manman / nz, Kazakh / nz, Nobel / nz, MAO Dun / nz,

Kazakh / nz / n, Gaoshan / nz / n, Anhui / ns / n

③ contains a proprietary name (or abbreviation), marked nz, with square brackets.

Jinpu Road / nz, Shitai Line / nz, [Beijing / j 9 / j Railway / n] nz,

[Beijing / j-jin / j expressway / b highway / n] nz,

[Beijing / ns- / w Seattle / ns route / n] nz

Specialized names such as important events and movements in the history of ④ are generally phrases, which are processed according to the proprietary names of the phrase type and marked as nz.

[Lugou Bridge / ns Incident / n] nz, [Xi'an / ns Incident / n] nz, [May 4th / t Movement / n] nz

[Meiji / nz Restoration / n] nz, [Sino-Japanese / t War / n] / nz

⑤ proper names after the polysyllabic nouns, such as "language", "literature", "culture", "way", "spirit", etc., should be divided.

Europe / ns languages / n, French / ns literature / n, Western / ns culture / n,

Beethoven / nr Symphony / n, Lei Feng / nr Spirit / n,

American / ns way / n, Japanese / ns cuisine / n, Song dynasty / t antique / n

Some people also think that "doctrine" is a posterior component, and often followed by another posterior component "person", so the "doctrine" with its proprietary name as a partitioning unit (see: 3.2 (2) d of ③).

Marxism / n, Marxism-Leninism / n, Truman doctrine / n,

Marxist / n, Leninist / n, Socialist / n

The ⑥ trademark (including the special name and the subsequent "card", "type", etc.) is specifically refers to, marked as nz, but then the goods are still marked with the common noun n.

Master Kong / nr instant noodles / n, Zhonghua brand / nz cigarettes / n, Peony Type III / nz TV / n

Lenovo / nz computer / n, crocodile / nz shirt / n, Nike / nz shoes / n

⑦ names named after a serial number are generally not considered proprietary names.

National Road 2 / m / q / n, 11th / m / q third Plenum / j

If you have a special name in front, it can be combined as a phrase type special name.

[China / ns 101 / m National Road / n] nz, [j / 11th / m / Third Plenary Session of q / j] nz

The names of ⑧ books, newspapers, magazines, documents, reports, agreements, contracts, etc., are usually marked by title numbers and are not used as proper nouns.Since these names tend to be longer, the names themselves are routinely handled.

"/ w Ningbo / ns Daily / n" / w, "/ w Lu Xun / nr Complete Works / n" / w,

Zhonghua / nz reading / vn newspaper / n, Du Fu / nr poetry selection / n,

/ w Volkswagen / n Medicine / n / w, Deng / nr Xiaoping / nr Wen Xuan / n

The titles of a few income dictionaries, newspaper names and other proprietary names, are not divided.

A Dream of Red Mansions / nz, People's Daily / nz, The Scholars / nz

⑨ When some special names do not tell whether they are body names, nz.

[Paris / ns Belch / nz Gymnasium / n] nt,

Among them, "Belshi" had to be temporarily labeled as nz.

⑩ general naming activity is often expressed in quotation marks, and is not regarded as a proprietary name.

Welcome / v Hong Kong / ns return / v Beijing 9 / j plant green green protection / l activity / v n

Third / m times / q Yokota / ns base / n noise / n litigation / vn

The name of the dish on the recipe is usually a phrase type, if opened, the meaning is far different, then do not cut, otherwise cut.Even if not divided, it is not regarded as a proper noun.

Kung Pao diced / n, sweet clover / n, squirrel mandarin fish / n, braised pork in soy sauce / n,

Eggs / n soup / n, sesame / n cake / n, shredded chicken / n noodles / n

△ (5) Digital and quantitative phrases

The ① base, ordinal, decimal, score, and percentage shall not be divided, as a dividing unit, marked as m.

1.23 / m, 1.2 million / m, 123.54/m, one / m,

First / m, 35 / m, 20% / m, two-thirds / m, 30-thousandth / m

"Several" and "zero" belong to the basic coefficient words (or digit words), so the "several" and "zero" cardinals, ordinal, decimal, scores, percentage of "zero" are not divided.

Dozens of people / m people / n, hundreds of thousands / m yuan / q, 101 / m / q

② approximate number, before plus adverbs, adjectives or after adding "come, more, left and right" and other auxiliary words should be divided.

About / d hundred / m over 10 / m / m, only / d hundred / m / q, 40 / m to / m / q,

More than 20 / m / m only / q, ten / m / q, about 30 / m / m,

Dozens of people / m people / n, hundreds of thousands / m yuan / q, nearly / a 20 / m years / q to / f

Two digital words connected and "hundreds", "thousands" and so on will not be divided.

Five or six / m years / q, seven or eight / m days / q, seventeen / m years old / q, one hundred / m students / n,

Thousands / m people / n, thousands of / i / u masses / n

If the punctuation marks are inserted between the two connected numbers, they should be divided.in compliance with:

Five and six years ——> five / m, / w six / m years / q,

——> 9 / m, / w 10 o'clock / t

③ quantitative phrases should be divided into words and quantifiers.

Three / m / q, 10 / m kg / q, one / m box / q dim sum / n

* But a few quantifiers that are already the login units of the dictionary are no longer split.

One / m, some / m ("some" also used as a partitioning unit in "word specification")

The "number + name" structure of the ④ table order relationship should be divided into sections.

Such as: two / m company / n, three / m department / n

△ (6) Time words

① year, month, day, time and seconds, by year, month, day, time, minutes, seconds, marked as t.

1997 / t March / t 19 / t, 98 / t October / t 8 / t,

March / t 10th / t PM / t 2 pm / t 18 pm / t

Attention should be paid to the distinction between time words and quantitative words, for example: "78 years" means "1978" 1978 "should be marked as" 78 years / t ", when the number of" 78 years "should be marked as" 78 / m years / q ".Another example is two / m / q month / n, three / m day / q time / n.Similarly, when "8 days" is the eighth day of a month, it is not divided as "8 days / t"; if 8 days, separate as "8 / m day / q".

If there is no representation of the time after the number of "year, month, day, hours, minutes, minutes, seconds" and so on marked as the number word m.

Chinese / n Computer / n International / n Conference / n'/ w 96 / m

1998 / m Chinese / n Information / n Processing / vn International / n Conference / n

The name of *② historical dynasty is marked as t.

Western Zhou / t, Qin / t, Eastern Han / t, Southern and Northern Dynasties / t, Qing / t

The "Year of the Ox and the Year of the Tiger" shall not be divided and marked as:

Year of the Bull / t, Year of the Tiger / t

"First Sino-Japanese Year, Gengzi, Wuxu" is not divided, marked as:

Sino-Japanese / t, Sino-Japanese / t war / n, boxer / t indemnity / n, 1898 / t reform / n

△ (7) The monosyllabic pronoun "Ben", "each", "each", "zhu", and the subsequent monosyllabic noun shall be divided.

This newspaper / r, per person / r, our / r, this / r area / n, each / r department / n

△ (8) distinguishing words

① is generally a tangential unit and labeled with sex of words b.

Female / b driver / n, gold / b bracelet / n, chronic / b gastritis / n, ancient / b coin / n

Associate / b Director / n, General / b company / n

② monosyllabic distinguishing word and monosyllabic noun or name morpheme combination, as a partiated unit, and marked with noun sex n.

Rooster / n, female / n, demon / n, ancient coins / n

*③ a few "monosyllabic distinguishing words + two-syllabic words" structure as a word earned into the dictionary, is no longer divided.

general secretary /n

△⑼ verb plus verb or verb plus adjectives composition structure

The-syllable structure of ununpaid dictionary, if apart is one word, usually as two partitioning units.

Go / v to / v, bump / v on / v, tune / v good / a, sit / v steady / a

If disassembled, and at least one of them is a morpheme, it is usually not divided as a dividing unit.

Form / v, agitation / v, description / v, vibration / v

The insertion of "get" or "no" in the middle of the double-syllabic complement structure should generally be divided into sections,

Go / v to / u to / v, go / v not / d to / v, Ann / v to / u on / v, Ann / v no / d on / v

However, if the "get" or "no" is removed, the front and after the two words do not constitute a word, then as a participle unit.

Too late / v, too late / v, worthy of / v, sorry / v, can say / l, can't say / l

Some are a portmanteau after removing "get" or "no", but at least one of them is a morpheme, which is difficult to understand, still as a segmentation unit.

Form is / v, form is not / v

*⑽ phrases of more than four words should usually be divided.

Summary / v / experience / n, implementation / v execution / v, survey / v study / v,

One / m slow / a 2 / m watch / v 3 / m through / v

But words such as "means of production / n", "national economy / n", and "relations of production / n" are used as a word

The code is no longer divided.

*⑾ idiom or idiom is a partiated unit and marked with character of words i or l.

With / i, thriving / i, as well known / i, thus / l, the beginning of the New Year / l

⑿ idioms or idioms with more than four words are generally not divided and marked as l or i.

The first thing to get the month / i, a year's plan lies in spring / i,

No matter 3721 / i, everyone adds firewood fire high / i, iron rooster a hair without pulling / l,

Sell dog meat under the label of a sheep head /i.

An idiom separated by punctuation marks in the middle is divided first, and then enclosed in square brackets and marked as I.（

Hang sheep head, sell dog meat ——> [hang sheep head / i, / w sell dog meat / i] i

Further rod, ——> [further rod / i, / w further / i] i

No sky, no ground ——> [no day / i, / w no ground / i] i

⒀ expresses an abbreviation of a complete concept or set as a tangent unit and is labeled j.

Meros / j, ADC / j, UNESCO / j, sideline fisheries / j, Central / Western / j

* In the case of separate ton numbers, split up:

De / j, / w Italy / j, / w Day / j, Port / j, / w Australia / j, / w station / j,

Hong Kong / j, / w Australia / j compatriots / n,

Lin / j, / w shepherd / j, / w associate / j, / w fishery / j, etc. / u sideline / n

If the last abbreviation can be synthesized into a word with the latter word (morpheme), it will not be divided separately.

Agriculture / j, / w forest / j, / w pastoral / j, / w associate / j, / w fishery / n

When the abbreviation of national name and place name is juxtaposed together, even if there is no ton number in the middle, it should be separated.

China / j beauty / j cross / v country / n company / n

[Beijing / j Jin / j Tang / j area / n] / ns

China / j / j joint / vn Bulletin / n

Hong Kong / j Australia / j Taiwan / j compatriots / n

A special form of an abbreviation expressed in brackets

Building (structure) building ——> building (structure) building / j

Martial Arts Museum (school) ——> Martial Arts Museum (school) / j

Country (border) border ——> country (border) border / j

Hall (Bureau) Director ——> Hall (Bureau) Director / j

Processing of the *⒁ morpheme and non-morpheme characters

The morpheme and non-morpheme words are generally not used as partitioning units, except in the following special cases.

① Certain dual-syllabic clutch words are used separately, one of which is a morpheme that can be labeled as a morpheme.

Out / v over / u two / m days / q difference / Ng, reason / v / u one / m times / q hair / Ng,

Wash / v / u a / m comfortable / z / u bath / Vg

A ② single-word noun or noun morpheme followed by a simple orientation word should usually be synthesized into a place word or time word, but in order to coincide with the "word segmentation specification" and for the convenience of machine translation in China, the following processing methods are adopted here:

a.The combination of "single-word noun + single-word orientation word" is divided into two units.

Rice / n before / f, tree / n on / f, bag / n inside / f, bed / n under / f

1. The structure of "single word noun morpheme word + single word orientation word" is a place word or time word.

Table / Ng on / f- -> Table on / s, afternoon / Ng post / f- -> afternoon / t,

Body / Ng top / f- -> body / s, chest / Ng front / f- -> chest / s

1. "Province, city, county, township, village, ministry, bureau, department, regiment, battalion, company, company, hospital, department, class" and other nouns "inside, on" and other azimuth words, still have the meaning of organization, organization, as a segmentation unit, marked as a noun.

Such as: ministry / n, county / n, village / n, department / n, class / n

③ non-morpheme words are labeled x.

"/ w Quail / n" / w / u "/ w Quail / x" / w word / n How to / r read / v?/w

* (15) Comments on the processing of non-Chinese character strings in the text

1. Conventional or common symbols in science and technology maintain their original meaning, according to their original meaning

Determine the corresponding mark.

Arabic numerals: Room 121 / m room / q / n

2000 / t August / t 15 / t

Individual Roman numerals: II / m

IX /m

xv /m

The English letters (or letter combinations) represent the common units of measure:

A stands for "amp", example: then pointer at 1A

Correct segmentation and marked as:

Then / c pointer / n finger / v at / p 1 / m A / q / n

V represents "volt"; W, "watt"; m, m; kg, kg; and so on.

② other English letters (or letter combinations or statements) are marked as nx, such as:

World Cup / n football / n A / nx group / n of / u / two / m / q match / vn

(Here, A acts as a pronoun)

A / nx Company / n, Mr.B / nx / n, Jun X / nx / Ng

(Here A (proprietary) a noun or pronoun)

24 / m K / nx Gold-plated / n

(The K here is actually a measure of pure gold, Chinese with "open", the computer marked it as nx, and people did not proofread out, not wrong, it is best to keep consistent.）

The C / nx is / v speed of light / n

Windows98/nx

Pentium IV /nx

I LOVE THIS GAME/nx

(Although this is an English sentence, it remains as a segmentation unit at this stage)

③ Other Western languages (Greek, Russian, etc.) are treated as in English.

④ Japanese kana processing with English.If the Chinese characters in Japanese texts use the Chinese characters in GB2312, they will be treated with the same language as Chinese;

If using JIS code, the same as English.

**3. Specification for combining segmentation and annotation**

The morphemes in Chinese are the basic unit of word formation.There are three main ways that morphemes form words: overlap, addition, and compound.The segmentation and annotation of these cases are specified as follows.

**3.1 Overlap:**

In Chinese, words are constructed in overlapping ways, including AA, AAB, A ABB, AABB, AB in A, A, A not AB, ABAB (A and B represent one Chinese character respectively). If this form is used as an entry in a grammatical information dictionary, its nature of words is determined.The following discussion is mainly about the absence in the dictionary:

⑴ "AA" overlapping form

The ① single-word verb overlap formula AA acts as a partitioning unit and is annotated as verb sex v.

Such as: walk around / v, listen to / v

② single-word adjective overlapping AA, some are words, some are not words.If the word does not follow "", as a partitioning unit, usually the adverb d.

Good / d dry / v bar / y, long / d no / d talk / v

If "land" is added later, the original provisions will not change, such as:

Gently / d / v / v / d gently / d / u / v down / v

Long / d ground / u no / d talk / v

However, if only the "of" or "ground", "AA" or "AA ground" is one

Cut units, marked as state word z.

Sweet / z dim / n, cut / v / u pointed / z,

Round ground / z sit / v one / m circle / q

③ single-word noun overlap AA, is a partitioned unit, and marked as noun sex n.

Everyone / n, every house / n

④ single-word quantifier overlap form AA, for a partitioning unit, and labeled with quantifier q.

Zhang Zhang / q, each / q

⑤ adverb overlap AA, a partiated unit and annotated as adverb sex d.

Often / d, just / d

⑵ "AAB" overlapping form

The ① VO structural form of the hypersyllabic clutch verb "AAB" overlapping form is a partiated unit and labeled verb sex v.

Take a bath / v, wave / v, and get a haircut / v

The overlapping AA plus "see" of ② monosyllabic verbs combine into a partitioning unit and are annotated as verb sex v

Try / v, look up / v, read up / v

⑶ "ABB" overlapping form

① disyllabic adjectives of overlapping form ABB, as syncopated units and annotated as state word z.

Alone / z, bright / z, alone / z

The "ABB" form of the ② quantitative structure is not divided, and is marked with quantifier m (with the attribute of quantifier).

One / m, waves / m, round / m

⑷ "AABB" overlapping form

The overlapping form of the ① 2-word verb "AABB" is a partitioned unit and is annotated with the verb v.

Draw in with / v, hook up with / v

The ② two-word adjective of the overlapping form "AABB" for a syncopated unit,

Happy / z, comfortable / z

If added "" or "ground", mark as:

Happy / z / u, comfortable / z ground / u

The overlapping form of the ③ two-word noun "AABB" is a partitioned unit and annotated as noun n.

Mountains and rivers / n, all aspects / n

The overlapping form of A "AABB" is a partiated unit and annotated as numbered word m.

Many more / m, more or less / m

⑤ has two nouns with opposite meanings and overlap the overlapping form "AABB" as a partitioned unit and marked as the state word z.

Large and small / z, high and low / z

⑥ The overlapping form "AABB" only in the adverbial position is marked as the adverb d.

Day and night / d, original / d, true / d

The form of AB "and" A not AB "in ⑸" A

The overlapping form of ① hypersyllabic adjectives, "A in AB", is a syncopated unit and is annotated as the state word z.

Mali sloppy / z, confused / z, flustered / z

② uses the form of positive and negative, generally cut separately.

Believe / v not / d believe / v, easy / a not / d easy / a

However, if the incomplete form of "A not AB" is formed, it will not be divided and marked as word v or z respectively.

Do not believe / v, tolerance is not easy / z, drift is not beautiful / z

⑹ "ABAB" overlapping form

The overlapping form of monosyllabic words, "ABAB", is cut apart, which mainly includes:

The "ABAB" of ① verbs such as: research / v research / v, sign / v sign / v

② adjective "ABAB" such as: happy / a happy / a, comfortable / a comfortable / a

③ count the words "ABAB" such as: many / m many many / m, many / m many / m

④ state word "ABAB" such as: white / z white / z, green / z green / z

⑤ quantifier "ABAB" such as: one / m one / m

The "ABAB" overlapping form of ⑺ bissyllabic words is separated as in other word classes, such as:

Wow, wow, ——> wow / o wow / o

Overlap cases of other forms of ⑻

The "V V, V V, V V" overlapping form is separated as verb phrases.

Talk / v one / m talk / v, think / v / u think / v, read / v / u one / m read / v

**3.2 Additional**

The ⑴ prejoin component + morpheme or word

A portmanteau consisting of "forward component + morpheme or word", is a syncopated unit.This can be subdivided into the following conditions:

① "" + monosyllabic noun or morpheme, forming a noun and marked n; if the morpheme is the specific name of a person, marked nr.

Such as: Ge / n, hua / nr

② "small" or "old" or "big" + monosyllabic surname word, constitute a proper noun, marked with nr.

Such as: Xiao Wang / nr, Lao Zhang / nr, Dayang / nr

③ "old" or "small" + single-word base word (two, three, …, nine), constitute a noun and marked with n.

Such as: second / n, six / n, small third / n

The new portmanteau of ④ ("no", "super", "no", "too",...) may maintain the nature of the original word or change the nature of words.

Such as: non-metallic / n, supersonic / b (sound / n), ultrasonic / n, pollution-free / v (public hazard / n),

Unconditional / d (condition / n), supersaturation / z (saturation / a)

If the anterior component of "non" has more than one word, it is still separated.

Such as: non- / h country / n work / vn personnel / n, non- / h city / r registration / vn vehicle / n

⑵ morpheme or word + posterior component

Any compound word composed of "morpheme or word + back component" is a partitioning unit.The details are as follows:

① #+ "son" (# for any morpheme or word, the same below)

Words are generally nouns, such as: flower / n, painting / n

There are exceptions: one / m heap / q, play / v, bounce / v, roll round / z, good / d, good / z

The ② #+ "' s."

a.The "People" of the noun plural are divided separately and marked with k.in compliance with:

Friends / n guys / k, kids / n / k

b.The combination of "people" in two words or "people" with the previous noun (Kerhua) is meaningless and combined as a partitioning unit and marked n.in compliance with:

People / n, boys / n, men / n, young / n men / n, young / n men / n

③ has a posterior component

a.By the subsequent composition of "home", "member", "sheng", "long (zhang3)", "sex", "machine" and so on, generally nouns.Such as: Artist / n, Clerk / n, Worker / n, Graduate / n, Chief of Staff / n, Revolutionary / n, Disk Machine / n

b.Composition words composed of "head (tou5)" and "son (zi5)", generally nouns, such as:

Head head / n, yard / n

However, there are also special cases, such as: front / f, back head / f

It should be noted that the real "head (tou2)" and "son (zi3)" are not regarded as the back components, try to compare:

Head / n (dui4tou5), header / a (dui4tou2)

Brick / n (zhuan1tou5), bullet / n head / n (tou2)

Table / n (zhuo2zi5), Go / n sub / n (zi3)

c.# + "", generally composed of verbs such as: standardization / v, diversification / v; exceptions: four modernizations / j, idealization / a.

d.# +", precby a shorter word or phrase, forms a partiated unit together with the preceding word, marked as n; by a longer phrase or sentence, separated, marked as k.

Investigator / n, explorer / n, seeker / n, repeat offenders / n

After / p hard / d pursuit / v and / c to obtain / v happiness / a person / k

No / d / gu / v advice / v and / c determined / vd riot / v person / k

The ④ word, with multiple posterior components, is still a syncopate unit.

Physics / n, Physicist / n, linguistics / n, Linguistics / n

⑶ forward component + morpheme or word + posterior component, this form of the commanteau, is also a partitioning unit.

Non-party member / n, anarchist / n, super-large / b

⑷ Note: The monosyllabic distinguishing words are treated differently from the forward components, see 2.2 ⑻.

**3.3, compound words**

The "compound" way can combine two word components into a new word.Word formation components are generally considered as morphemes.Because the composition of compound words and the composition of phrases are the same, including definite, form, description, narration, complement, subject and predicate, union, connection and so on.When the morpheme is a vocabulary element, the boundary between the compound word and the phrase is unclear.Only when at least one of the word components is not words, can we be sure that the structure of the new combination is an unlogged word, otherwise there is a certain elasticity.Formally, a combination of two or three words can be broadly considered as a single word.The name indicator used below is labeled as a noun with n or a name morpheme labeled as Ng."Form", "the meaning of" movement " can be similar.

⑴ word noun

① "name + name" fixed structure, generally for a syncopate unit.

Beef / n, aluminum pot / n, enemy camp / n

② "dynamic + name" fixed structure, generally for a syncopate unit.

Cooking / n, roast / n, legging / n, come letters / n, lovers / n

If the ③ "move + name" is an object structure, it is a phrase, which should be cut and separated.

I / r like / v eat / v BBQ / n./w

I / r come / v roast / v meat / n eat / v./w

However, some tightly combined or using stable reference structures have been logged in in the dictionary, and they are processed into a partitioning unit (clutch word), marked as the verb v, such as: meal / v, bath / v, speech / v.

The fixed structure of ④ "shape + name", if the middle can not be inserted "" or "" after the meaning change, as a divided unit; otherwise, should be divided.

Black tea / n, bitter gourd / n, red flower / n (a medicine)

Small / a bed / n, white / a flower / n, red / a flower / n

⑵ three-word noun

① "dynamic (double tone) + name (single tone)" in the structure, generally a syncopated unit.

Consumable / n, letter / n, relief food / n, control valve / n

② "name (double tone) + name (mono)" structure, usually a partitioned unit, but greater flexibility, if the previous double syllable noun and the following single syllable noun combination after the same meaning, can also be separated

Jean / n, Ammeter / n, Tropical fish / n, Hebei / ns man / n, watch / n factory / n

③ "name (single tone) + name (double tone)" structure, usually a partitioning unit, but greater flexibility, if the previous single syllable noun and the following double syllable noun combination after the same meaning, can also be separated

fingernails / n, horse tail / n, electric / n jug / n

④ "shape (single tone) + name (double tone)" of the fixed structure, processing principle with the two words "shape + name" combination

Little law / n, old girl / n

White / a granulated sugar / n, sweet / a rubber / n, sweet / a snack / n

⑤ "-shaped (double sound) + name (single)" fixed structure, the processing principle is the same as ④

Beautiful Island / n, Poverty / a County / n, Rich / a Village / n

⑶ simple orientation word + name (single tone) fixed structure, for a syncopated unit.The synthisms composed are generally premises words, but in some special cases they may be nouns or time words.

Front yard / s, back house / s, backstreet / s

Left shoulder / n, fork / n, day before / t, day after tomorrow / t

**⒋ annotation specification**

**4.1 Relationship between word annotation and grammar information dictionary**

According to the Modern Chinese Grammar Information Dictionary, for those words without concurrent information, the nature of the words can be determined while dividing them.In this way, the annotation specification focuses on describing the speech character of those multi-class words, namely how a correct speech character marker is selected in a specific context.

⑴ Because the automatic annotation is based on the grammatical information dictionary, most of the annotated information comes from the dictionary, which is relatively reliable, but due to the existence of "choose one more" and "guess" the nature of words, so the correctness of words still needs to be identified.

⑵ due to sufficient information in the context, the text of the word annotation relative to the word classification is easy, but in the grammar system of Peking University should adhere to the versatility of the word class, main prevention tendency is only according to the function in the current sentence to determine the word, such as the subject and object position of the word as nouns, this is not appropriate.

⑶ Due to the space limitation of dictionary, not only has the problem of unregistered words, but also the incomplete problem of logged words.If some nouns can be used as quantifiers ("one / m ship / q water / n" "ship" is a quantifier), the dictionary may only describe it can be a temporary quantifier, but does not clearly stipulate that it is a quantifier category, then should still be determined by the actual function of the text.Another example is "new", where the dictionary is only an adjective, and some people think that "new" in "new classmate" is a distinguishing word, which is also labeled "new / b classmate / n".This will fully explore the nature of speech that each word form may have.As for the new sex of words after whether the dictionary is to consider other factors.

**4.2 Selection of words for common multiclass words**

Due to the characteristics of text data, the machine cannot distinguish between homophones and homophones, which causes the word class ambiguity phenomenon in the process of Chinese word class annotation.Words with this phenomenon can be generally called as multi-class words.Here are some annotation principles of multi-class words.

The ⑴ n-q multi-class case.

Some nouns in Chinese (mainly monosyllabic nouns) can be used as quantifiers. For these words, the character of the words in the sentence is determined according to the context.

① count words + n-q + n, take q.

One / m car / q coal / n, three / m barrel / q water / n

In addition, some nouns in Chinese make temporary quantifiers and can only be followed by "one". In this case, it should also be labeled as the quantifier q.

Do / v / u one / m table / q dish / n, raw / v / u one / m stomach / q gas / n

② "this", "that", "per" etc for indicator pronouns + n-q + n, take q.

This / r bed / q quilt / n, this / r door / q affinity / n

③ Other cases, generally take n.

On / v car / n, enter / v door / n, buy / v car / n, send / v bucket / n to / v site / s

⑵ a-v multiclass case

① If the word has a true object in the sentence, it is marked as v.

He / r with / p she / r no / d red / v over / u face / n,

Prosperity / v market / n, correct / v attitude / n

② If the word is modified by "very", it is labeled a.

This / r flower / n very / d red / a, market / n very / d boom / a

③ If the word modifies a noun, it should generally be marked as a.

Prosperity / a / u scene / n, red / a paint / n, consolidation / a / u defense / n

④ If the word complements a verb, it shall be marked as a.

Put / v understand / a some / m

Up / v red / a / u face / n

⑶ v — n multi-class case

In fact, it refers to the broad and parallel phenomenon.When the word represents an action, it is followed by a true object, and it is v; when it refers to people or things, it is n.

Editor / v Technology / n literature / n

She / r is / v responsibility / n editor / n

To / v lock / v on / v door / n

Forget / v / u buy / v one / m handle / q lock / n

Timely / ad report / v head / n

One / m / q important / a report / n

⑷ p-v multi-class case

These words mainly include "in", "to", "in", "to" to "," with "to", "to", etc. They are mainly based on the following methods:

① considers the grammatical function and distribution of a word. If the word (including "with, over, over") speaks alone or makes predicates alone, it is a verb.

"You / r dad / n in / v not / d at / v?/ w "" In / v./w ”

Beijing / ns to / v over / y, Singapore / ns I / r to / v over / u

Don't / d old / d with / v / u, let's / r ratio / v 1 / m ratio / v

② to the structure of "p-v + other components", if spoken or alone, where p-v is a verb; if not alone or alone adverbial or complement, where p-v is a preposition.Try to compare:

| verb | preposition |
| --- | --- |
| He / r not / d in / v classroom / n | He / r in / p classroom / n self-study / v |
| He / r at / v not / d at / v home / n?—— in / v | We / r walk / v on / p campus / n on / u path / n on / f |
| Train / n has / d to / v / u Beijing / ns | Lao Wang / nr to / p Beijing / ns business trip / v to / v / u |
| To / v no / d to / v station / n?—— to / v for / u | From / p east / f to / p west / f total / d long / a 30 / m m / q |
| Dog / n total / d heel / v / u owner / n | I / r often / d with / p he / r learn / v Japanese / n |
| Sunflower / n to / v Sun / n | Athletes / n positive / d run / v to / p finish / n |

House / n toward / v south / f gate / n / p south / f open / v

The ⑸ p-c multiclass case

The common words are "and", "follow", "with", "and" and " are mainly based on the following principles: the words are prepositions if the anterior and posterior elements cannot interposition or the modification can be added to the words; If the anterior and posterior elements of the words, the words are conjunctions.

I / r with / c he / r both / d is / v college student / n

You / r don't / d follow / p he / r run / v

I / r with / p him / r consult / v questions / n

Note: The following sentences are ambiguous (the information in parentheses is the judgment criteria).

I / r (already / d) and / p he / r met / v / y./w

I / r and / c him / r (already / d) met / v / y./w

Therefore, combining contextual information is required to determine the correct word class marker.

The ⑹ b-d multiclass case

① This word is an adverbial.

We / r will / v common / d progress / v

Auto / d Cancel / v Order / n

② if this word is fixed, or "of" with "of" structure.

Common / b target / n is / v complete / v this / r item / q task / n

This / r / q / toy / n is / v automatic / b / u

The ⑺ c-d multiclass case

This kind of multiple words, mainly "but", "although", "but", "can" and so on.Generally speaking, if the word uses a verb (adjectives, verb) as an adverb.If this word mainly connects the sentence and the clause, indicating the semantic combination relationship between the clauses, such as transition and concession, it is a conjunctive.Try to compare:

① only

I / r received / v / u point / q injury / Ng, / w but / c not / d matter / a

He / r but / d casually / ad talk / v

② only

Rain / n stopped / v / y, / w but / c above ground / s still / d very / d wet / a./w

But / d see / v door / s paste / v / u one / m pair / q couplet / n./w

③ approve

Everybody / r though / c tired / a, / w can / c all / d very / d happy / a./w

She / r stay / v me / r can / d good / a / y.

④ although

Despite / c day / n under / v / u rain / n, / w he / r or / d departure / v / y./w

You / r though / d say / v, / w do not / d be afraid of / v./w

1. **About tagging vn, vd, an, ad**

These 4 markers are subsidiary markers of verb v and adjective A.When a word in a text is labeled vn, vd or an, ad, they are first considered verbs or adjectives, but they have a special grammatical nature, showing special grammatical functions in statements.These markers are sometimes given Chinese names, such as name verbs, just for convenience.

1. There is a partial two-syllabic verb, labeled vn when it has one of the following four syntactic functions in a syntactic structure:

a.Make the object for the special verb "yes".

1. Acting as a quasi-predicate object for formal verbs or other quasi-predicate object verbs.
2. Direct to act as the central language of physical sex phrases.
3. Without the auxiliary word "of", directly act as a modifier of the physical sex phrase.

Leader / n to / p this / r / q thing / n have / v consideration / vn

Conduct / v one / m visit / in / q visit / a / vn

Give / v serious / a treatment / vn

To try to / v to organize / vn

Grammar / n studies / vn very / d important / a

Must / d improve / v training / vn method / n

This / r study / vn thought / n very / d novel / a

Note that the verb serves directly as the subject or the verb is still labeled v, not vn.

Investigation / v is / v necessary / a / u

Our / u purpose / n of / n come / v is / v visit / v visit / v

Need / v inspection / v

Need / v investigation / v actual / a situation / n

Usually decide to label it as v or vn only within the hierarchy of the phrase structure where the verb is located.for instance,

① we / r survey / v purpose / n is / v understand / v actual / a situation / n./w

The ② large-scale / d survey / v language / n of / u actual / a use / vn situation / n is / v one / m term / q important / a

Of the / u base / n work / vn./w

③ via / p survey / v

④ via / p survey / v language / n of / u actual / a usage / vn situation / n

⑤ conducted the / v survey / vn

⑥ performed / v large-scale / b survey / vn

⑦ through / p language / n actual / a use / vn situation / u large scale / b / vn survey / v n

⑧ s / u massive / d survey / v through / p language / n actual / a use / vn situation / n

The notation for the "survey" is correct in all of the above eight examples.

In ①, "we" and "survey" first combine into a host-predicate structure and then modify the "purpose".If you add a "" of "before the" purpose ", the structure is clearer and reads smoother.But in writing, this "kind of" is often saved.If "of" is added between "we" and "survey", "survey" and "purpose" first constitute the definite structure, then "survey" shall be marked as vn.There are disambiguation here.

In ②, "investigation" or the medium structure with "mass", or the object structure with "actual use of language", should be marked as v.

In ③, "survey" itself makes the object of the preposition "through".Within the grammatical system followed by the Code, a preposition can carry a predicate object.The reason why "investigation" is a verb can be found in ④.

In ④, "investigation" first constitutes the predicative object structure with "the actual use of language", and then makes the object of the preposition "through".

In ⑤, "investigation" is a quasi-predicate object of the formal verb "proceeding", of course labeled vn.

In ⑥, the quasi-predicate object "survey" of "progress" can be made attributive, and "large-scale" should be labeled as distinguishing words.

The annotation for "large surveys" is not the same in ⑦ and ⑧.Why are they all right before?First, the ⑦ is right.On the whole, the "large-scale investigation of the actual use of language" is subjective language, and the "large-scale investigation" of the mental language is also labeled as the definite structure of subjective language, which will not cause controversy.In ⑧, "large-scale investigation" is labeled as a predicate trait structure.Is this calculation not wrong?Are there any theoretical difficulties?The previous said "preposition can take predicate object" is an important argument of the "phrase standard" grammatical system, while here that "the central component of verbal phrases can be predicate component" is another more important and theoretical argument that the "phrase standard" grammatical system insists on.To this, Mr.Zhu Dexi has long elaborated.Adhere to this argument, it is more convenient to analyze the following syntactic structure.

Need / v support / v

Need / v support / v have / v creative / n / u exploration / vn

Need / v masses / n / u support / vn

Needs / v crowd / n / u strong / d support / v

"Mass support" is a verbal phrase, in which "support" is marked as vn.The complete understanding of vn should be:

"Support" is first the verb, but acts as a noun in this specific syntactic position."Strong support of the masses" is also a verbal phrase, in which the heart language "strong support" is the predicate, is the form of the structure.In this structural hierarchy "support" is the verb v and "vigorously" is the adverb d.Since "vigorously" has only one adverb sex, so that the analysis will not be difficult.If it is argued that "the central component of the verbal phrase can only be the verbal component", "support" can be marked as vn, but "vigorously" as an adverb can not modify the verbal component.Similarly, there:

Steel / n production / n / u step / d increase / v

Here "step" has only one adverb.

Annotation may be ambiguous when the context information is not sufficient.If the "survey" in the ① above is labeled as v or vn, it can not be miscalculated.In ⑦ and ⑧, "large-scale surveys" also have both acceptable annotation results.

The "field investigation" in the "field investigation is important" is ambiguous.There are two scaling methods.

Site / s visit / v is / v important / a / u

(Go to / v site / s visit / v process / n process / n is / v important / a / u)

Site / s visit / vn is / v important / a / u

(/ v / m / q site / s / vn / v important / a / u)

If more context is missing and only "site inspection is important" is annotated, both labels are considered correct.

Note: The sex of "site" is the place word s, and the place word can be modified as an adverbial verb or an attributive noun.

⑵ is annotated as vd when the verb is directly adverbial.

He / r satire / vd said / v

Director / n stressed / vd notes / v

If the verb is followed by the "ground" adverbial, it is still marked as v.

He / r sarcastic / v ground / u said / v

Director / n stressed / v / u notes / v

1. As some verbs have noun characteristics, when some adjectives have one of the following three grammatical functions in the actual corpus, they are also considered to have noun characteristics, marked as an.
2. Made a "yes" object,
3. Acting as a quasi-predicate object of a quasi-predicate object verb,

c.Direct to act as the central language of physical sex phrases.

He / r has / v many / m distress / an

Here / s has / v mystery / an

Maintain / v environment / n of / u neat / an

Traffic / n safety / an is / u of / v first / m / m to / v attention / v

It should be noted that the object of the adjective that is directly the subject or the predicate verb is still marked a, not an.

Need for / v effort / a

Need / v further / d effort / a

⑷ adjectives when direct adverbial, marked as ad.

Study / ad seriously / v Deng Xiaoping Theory / n

In-depth / ad research / v syntax / n favorable / a in / p / natural / a language / n processing / vn technology / n / u progress / vn

When the adjective is followed by the "ground" adverbial, the adjective is still marked as a.

We / r should / v deep / a ground / u study / v syntax / n

**5. Postscript**

This specification was put forward and presided over by Yu Shiwen.Lu Jianming, Zhu Xuefeng, Duan Huiming, Kang Shiyong, Sun Honglin, Wang Hui, Zhao Qiang, Zhan Weidong and others participated in the formulation of this specification.Yu Shiwen drafted the draft of this specification, and Professor Lu Jianming revised the draft carefully.Then, there was a trial draft.During the formulation and trial process, the manuscript was revised several times and absorbed some opinions from Guo Rui, associate professor of the Chinese Department of Peking University, forming the "March 1999 edition".In addition to the staff of Peking University, the experts and technicians of Fujitsu Company put forward the basic requirements for the processing of proper terms and participated in the formulation of this specification.

In processing in six months before 1998, the peoples daily corpus, fujitsu experts and technical personnel through acceptance of computational linguistics of Peking University provides the consistency of corpus processing puts forward valuable opinions, collaboration unit of Shanghai normal university, Yantai normal college, Nanjing normal university, Beijing language and culture university, Chinese language modernization society and other units of teachers and students also put forward some opinions.Yu Shiwen, Duan Huiming, Zhu Xuefeng and Sun Bin from the Institute of Computational Linguistics of Peking University absorbed these opinions and revised the "March 1999 edition" to form the current "July 2001 edition"

Continuing criticism and correction of this regulation are welcome.After more practice, further modifications and additions are considered.
