# Supplementary material for: Constructing marine expert management knowledge graph based on Trellisnet-CRF
Source: PeerJ Comput Sci. 2022 Sep 5;8:e1083. doi: 10.7717/peerj-cs.1083 (PMC9455288; doi:10.7717/peerj-cs.1083)
Supplement: Supplemental Information 2 [file peerj-cs-08-1083-s002.zip › Peopleí»s Daily Corpus-raw data/corpus_readme_English.doc]

**People's Daily marks the instruction of corpus (PFR)**

This article is the instruction manual of the PFR annotation corpus to help users understand it and use it better.

puretextWord segmentation and sex of speechlanguagebeltrememberThe PFR corpus is made by marking the corpus of the People's Daily in the first half of 1998, and is arranged in strict accordance with the date, edition order and article order of the People's Daily.Every word in the article has a sex of word mark.The current marker set has 26 basic word markers (noun n, time t, place s, f, number m, word q, word b, pronoun r, verb v, adjective a, state, z, adverb d, preposition p, c, word, u, tone y, sigh e, o, idiom i, idiom l, abbreviation j, forward component h, back component k, morpheme g, non-morpheme x, punctuation w), From the perspective of the corpus application, Added proper nouns (person name nr, place name ns, organization name nt, other proper nouns nz); Some markers were also added from a linguistic perspective, In total, 4 markers were used.More than 0

1. Mark instructionsconcise and to the point

| code | name |
| --- | --- |
| Ag | Phenomenon |
| a | adjective |
| ad | Paragon words |
| an | adnoun |
| Bg | Differential morpheme |
| b | Differential words |
| c | conjunction |
| Dg | Parmorphemes |
| d | ad |
| e | interjection |
| f | noun of locality |
| g | morpheme |
| h | Front-connection ingredients |
| i | set phrase |
| j | Jane slightly language |
| k | Rear component |
| l | Xi language |
| Mg | Number of morphemes |
| m | numeral |
| Ng | The name of morpheme |
| n | noun |
| nr | name |
| ns | place name |
| nt | Institutional groups |
| nx | Foreign characters |
| nz | Other special name |
| o | onomatopoetic word |
| p | preposition |
| Qg | Quantitative morpheme |
| q | classifier |
| Rg | The morpheme |
| r | pronoun |
| s | Place words |
| Tg | Time morpheme |
| t | word denoting time |
| Ug | Helpsin |
| u | auxiliary word |
| Vg | Mobile morpheme |
| v | verb |
| vd | A secondary verb |
| vn | Name verb |
| w | punctuation |
| x | Non-morpheme word |
| Yg | The tone of morpheme |
| y | statement label designator |
| z | State word |

1. format description
   1. The corpus is a plain text file, and each line in the file represents a paragraph or a title, and an article has several paragraphs, so in the corpora, an article is composed of multiple lines.
   2. Each line begins with a numbering.For example, "19980101-01-001-001" means that this paragraph is the 001 paragraph of article 001 of article 01 on January 1,1998. The four parts separated by short horizontal lines are "year-month-edition number-chapter number-paragraph number" in order.The mark is also marked as a word, and the nature of the words is fixed as "m (number of words)".
   3. The paragraphs in an article are not empty, between the two articles, there will be a empty line, indicating the dividing line, at the same time, the "chapter number-paragraph number" of the next article will change.
   4. After the marking, are 2 single-byte spaces and start the body.
   5. The text part has been cut into words according to the specification, and added annotation, the format of "words / word", that is, words followed by a monocline, followed by the nature of the word mark.Two single-byte spaces separated between words.The last word in each paragraph, which also has 2 single-byte spaces after marking, keeps the consistent format.need
   6. In addition to the word marks, there are also "phrase markers", which generally appears in the organization group names, idioms and so on.For example, in "through / p [central / n people / s / n radio / vn radio / n] nt, / w", the part combined with "[]" is a whole organization group name, with square brackets followed by nt, and two single byte blank spaces to maintain the same format.intactfit
2. example

19980101-01-001-001 / m Towards / v Full / v Hope / n / u New / a Century / n —— / w 1998 / t New Year / t Speech / n (/ w attached / v pictures / n 1 / m / q) / w

……

19980101-01-001-006 / m On / p 1998 / t coming / v / f, / w I / r ten / m happy / a / u via / p [Central / n People / n Radio / vn Radio / n] nt, / w [China / ns International / n Radio / vn Radio / n] nt and / c [Central / n TV / n] nt, / w To / p nationwide / n / ethnic / r people / n, / w to / p [Hong Kong / ns Special / a Administrative Region / n] ns compatriots / n, / w Macau / ns and / c Taiwan / ns compatriots / n, / w Overseas / s Overseas Chinese / n, / w To / p world / n countries / r of / u friends / n people / k, / w / v / sincere / a / u greetings / vn and / c good / a / u wishes / vn!/w
