# Supplementary material for: Constructing marine expert management knowledge graph based on Trellisnet-CRF
Source: PeerJ Comput Sci. 2022 Sep 5;8:e1083. doi: 10.7717/peerj-cs.1083 (PMC9455288; doi:10.7717/peerj-cs.1083)
Supplement: Supplemental Information 3 [file peerj-cs-08-1083-s003.zip › kgocean/static/assets/chart-master/docs/index.html]

Chart.js Documentation


# Chart.js Documentation

## Everything you need to know to create great looking charts using Chart.js

# Getting started

## Include Chart.js

First we need to include the Chart.js library on the page. The library occupies a global variable of `Chart`.

```
<script src="Chart.js"></script>
```

## Creating a chart

To create a chart, we need to instantiate the Chart class. To do this, we need to pass in the 2d context of where we want to draw the chart. Here's an example.

```
<canvas id="myChart" width="400" height="400"></canvas>
```

```
//Get the context of the canvas element we want to select
var ctx = document.getElementById("myChart").getContext("2d");
var myNewChart = new Chart(ctx).PolarArea(data);
```

We can also get the context of our canvas with jQuery. To do this, we need to get the DOM node out of the jQuery collection, and call the `getContext("2d")` method on that.

```
//Get context with jQuery - using jQuery's .get() method.
var ctx = $("#myChart").get(0).getContext("2d");
//This will get the first returned node in the jQuery collection.
var myNewChart = new Chart(ctx);
```

After we've instantiated the Chart class on the canvas we want to draw on, Chart.js will handle the scaling for retina displays.

With the Chart class set up, we can go on to create one of the charts Chart.js has available. In the example below, we would be drawing a Polar area chart.

```
new Chart(ctx).PolarArea(data,options);
```

We call a method of the name of the chart we want to create. We pass in the data for that chart type, and the options for that chart as parameters. Chart.js will merge the options you pass in with the default options for that chart type.

# Line chart

## Introduction

A line chart is a way of plotting data points on a line.

Often, it is used to show trend data, and the comparison of two data sets.

## Example usage

```
new Chart(ctx).Line(data,options);
```

## Data structure

```
var data = {
	labels : ["January","February","March","April","May","June","July"],
	datasets : [
		{
			fillColor : "rgba(220,220,220,0.5)",
			strokeColor : "rgba(220,220,220,1)",
			pointColor : "rgba(220,220,220,1)",
			pointStrokeColor : "#fff",
			data : [65,59,90,81,56,55,40]
		},
		{
			fillColor : "rgba(151,187,205,0.5)",
			strokeColor : "rgba(151,187,205,1)",
			pointColor : "rgba(151,187,205,1)",
			pointStrokeColor : "#fff",
			data : [28,48,40,19,96,27,100]
		}
	]
}
```

The line chart requires an array of labels for each of the data points. This is show on the X axis.

The data for line charts is broken up into an array of datasets. Each dataset has a colour for the fill, a colour for the line and colours for the points and strokes of the points. These colours are strings just like CSS. You can use RGBA, RGB, HEX or HSL notation.

## Chart options

```
Line.defaults = {
				
	//Boolean - If we show the scale above the chart data			
	scaleOverlay : false,
	
	//Boolean - If we want to override with a hard coded scale
	scaleOverride : false,
	
	//** Required if scaleOverride is true **
	//Number - The number of steps in a hard coded scale
	scaleSteps : null,
	//Number - The value jump in the hard coded scale
	scaleStepWidth : null,
	//Number - The scale starting value
	scaleStartValue : null,

	//String - Colour of the scale line	
	scaleLineColor : "rgba(0,0,0,.1)",
	
	//Number - Pixel width of the scale line	
	scaleLineWidth : 1,

	//Boolean - Whether to show labels on the scale	
	scaleShowLabels : true,
	
	//Interpolated JS string - can access value
	scaleLabel : "<%=value%>",
	
	//String - Scale label font declaration for the scale label
	scaleFontFamily : "'Arial'",
	
	//Number - Scale label font size in pixels	
	scaleFontSize : 12,
	
	//String - Scale label font weight style	
	scaleFontStyle : "normal",
	
	//String - Scale label font colour	
	scaleFontColor : "#666",	
	
	///Boolean - Whether grid lines are shown across the chart
	scaleShowGridLines : true,
	
	//String - Colour of the grid lines
	scaleGridLineColor : "rgba(0,0,0,.05)",
	
	//Number - Width of the grid lines
	scaleGridLineWidth : 1,	
	
	//Boolean - Whether the line is curved between points
	bezierCurve : true,
	
	//Boolean - Whether to show a dot for each point
	pointDot : true,
	
	//Number - Radius of each point dot in pixels
	pointDotRadius : 3,
	
	//Number - Pixel width of point dot stroke
	pointDotStrokeWidth : 1,
	
	//Boolean - Whether to show a stroke for datasets
	datasetStroke : true,
	
	//Number - Pixel width of dataset stroke
	datasetStrokeWidth : 2,
	
	//Boolean - Whether to fill the dataset with a colour
	datasetFill : true,
	
	//Boolean - Whether to animate the chart
	animation : true,

	//Number - Number of animation steps
	animationSteps : 60,
	
	//String - Animation easing effect
	animationEasing : "easeOutQuart",

	//Function - Fires when the animation is complete
	onAnimationComplete : null
	
}
```

# Bar chart

## Introduction

A bar chart is a way of showing data as bars.

It is sometimes used to show trend data, and the comparison of multiple data sets side by side.

## Example usage

```
new Chart(ctx).Bar(data,options);
```

## Data structure

```
var data = {
	labels : ["January","February","March","April","May","June","July"],
	datasets : [
		{
			fillColor : "rgba(220,220,220,0.5)",
			strokeColor : "rgba(220,220,220,1)",
			data : [65,59,90,81,56,55,40]
		},
		{
			fillColor : "rgba(151,187,205,0.5)",
			strokeColor : "rgba(151,187,205,1)",
			data : [28,48,40,19,96,27,100]
		}
	]
}
```

The bar chart has the a very similar data structure to the line chart, and has an array of datasets, each with colours and an array of data. Again, colours are in CSS format.

We have an array of labels too for display. In the example, we are showing the same data as the previous line chart example.

## Chart options

```
Bar.defaults = {
				
	//Boolean - If we show the scale above the chart data			
	scaleOverlay : false,
	
	//Boolean - If we want to override with a hard coded scale
	scaleOverride : false,
	
	//** Required if scaleOverride is true **
	//Number - The number of steps in a hard coded scale
	scaleSteps : null,
	//Number - The value jump in the hard coded scale
	scaleStepWidth : null,
	//Number - The scale starting value
	scaleStartValue : null,

	//String - Colour of the scale line	
	scaleLineColor : "rgba(0,0,0,.1)",
	
	//Number - Pixel width of the scale line	
	scaleLineWidth : 1,

	//Boolean - Whether to show labels on the scale	
	scaleShowLabels : true,
	
	//Interpolated JS string - can access value
	scaleLabel : "<%=value%>",
	
	//String - Scale label font declaration for the scale label
	scaleFontFamily : "'Arial'",
	
	//Number - Scale label font size in pixels	
	scaleFontSize : 12,
	
	//String - Scale label font weight style	
	scaleFontStyle : "normal",
	
	//String - Scale label font colour	
	scaleFontColor : "#666",	
	
	///Boolean - Whether grid lines are shown across the chart
	scaleShowGridLines : true,
	
	//String - Colour of the grid lines
	scaleGridLineColor : "rgba(0,0,0,.05)",
	
	//Number - Width of the grid lines
	scaleGridLineWidth : 1,	

	//Boolean - If there is a stroke on each bar	
	barShowStroke : true,
	
	//Number - Pixel width of the bar stroke	
	barStrokeWidth : 2,
	
	//Number - Spacing between each of the X value sets
	barValueSpacing : 5,
	
	//Number - Spacing between data sets within X values
	barDatasetSpacing : 1,
	
	//Boolean - Whether to animate the chart
	animation : true,

	//Number - Number of animation steps
	animationSteps : 60,
	
	//String - Animation easing effect
	animationEasing : "easeOutQuart",

	//Function - Fires when the animation is complete
	onAnimationComplete : null
	
}
```

# Radar chart

## Introduction

A radar chart is a way of showing multiple data points and the variation between them.

They are often useful for comparing the points of two or more different data sets

## Example usage

```
new Chart(ctx).Radar(data,options);
```

## Data structure

```
var data = {
	labels : ["Eating","Drinking","Sleeping","Designing","Coding","Partying","Running"],
	datasets : [
		{
			fillColor : "rgba(220,220,220,0.5)",
			strokeColor : "rgba(220,220,220,1)",
			pointColor : "rgba(220,220,220,1)",
			pointStrokeColor : "#fff",
			data : [65,59,90,81,56,55,40]
		},
		{
			fillColor : "rgba(151,187,205,0.5)",
			strokeColor : "rgba(151,187,205,1)",
			pointColor : "rgba(151,187,205,1)",
			pointStrokeColor : "#fff",
			data : [28,48,40,19,96,27,100]
		}
	]
}
```

For a radar chart, usually you will want to show a label on each point of the chart, so we include an array of strings that we show around each point in the chart. If you do not want this, you can either not include the array of labels, or choose to hide them in the chart options.

For the radar chart data, we have an array of datasets. Each of these is an object, with a fill colour, a stroke colour, a colour for the fill of each point, and a colour for the stroke of each point. We also have an array of data values.

## Chart options

```
Radar.defaults = {
				
	//Boolean - If we show the scale above the chart data			
	scaleOverlay : false,
	
	//Boolean - If we want to override with a hard coded scale
	scaleOverride : false,
	
	//** Required if scaleOverride is true **
	//Number - The number of steps in a hard coded scale
	scaleSteps : null,
	//Number - The value jump in the hard coded scale
	scaleStepWidth : null,
	//Number - The centre starting value
	scaleStartValue : null,
	
	//Boolean - Whether to show lines for each scale point
	scaleShowLine : true,

	//String - Colour of the scale line	
	scaleLineColor : "rgba(0,0,0,.1)",
	
	//Number - Pixel width of the scale line	
	scaleLineWidth : 1,

	//Boolean - Whether to show labels on the scale	
	scaleShowLabels : false,
	
	//Interpolated JS string - can access value
	scaleLabel : "<%=value%>",
	
	//String - Scale label font declaration for the scale label
	scaleFontFamily : "'Arial'",
	
	//Number - Scale label font size in pixels	
	scaleFontSize : 12,
	
	//String - Scale label font weight style	
	scaleFontStyle : "normal",
	
	//String - Scale label font colour	
	scaleFontColor : "#666",
	
	//Boolean - Show a backdrop to the scale label
	scaleShowLabelBackdrop : true,
	
	//String - The colour of the label backdrop	
	scaleBackdropColor : "rgba(255,255,255,0.75)",
	
	//Number - The backdrop padding above & below the label in pixels
	scaleBackdropPaddingY : 2,
	
	//Number - The backdrop padding to the side of the label in pixels	
	scaleBackdropPaddingX : 2,
	
	//Boolean - Whether we show the angle lines out of the radar
	angleShowLineOut : true,
	
	//String - Colour of the angle line
	angleLineColor : "rgba(0,0,0,.1)",
	
	//Number - Pixel width of the angle line
	angleLineWidth : 1,			
	
	//String - Point label font declaration
	pointLabelFontFamily : "'Arial'",
	
	//String - Point label font weight
	pointLabelFontStyle : "normal",
	
	//Number - Point label font size in pixels	
	pointLabelFontSize : 12,
	
	//String - Point label font colour	
	pointLabelFontColor : "#666",
	
	//Boolean - Whether to show a dot for each point
	pointDot : true,
	
	//Number - Radius of each point dot in pixels
	pointDotRadius : 3,
	
	//Number - Pixel width of point dot stroke
	pointDotStrokeWidth : 1,
	
	//Boolean - Whether to show a stroke for datasets
	datasetStroke : true,
	
	//Number - Pixel width of dataset stroke
	datasetStrokeWidth : 2,
	
	//Boolean - Whether to fill the dataset with a colour
	datasetFill : true,
	
	//Boolean - Whether to animate the chart
	animation : true,

	//Number - Number of animation steps
	animationSteps : 60,
	
	//String - Animation easing effect
	animationEasing : "easeOutQuart",

	//Function - Fires when the animation is complete
	onAnimationComplete : null
	
}
```

# Polar area chart

## Introduction

Polar area charts are similar to pie charts, but each segment has the same angle - the radius of the segment differs depending on the value.

This type of chart is often useful when we want to show a comparison data similar to a pie chart, but also show a scale of values for context.

## Example usage

```
new Chart(ctx).PolarArea(data,options);
```

## Data structure

```
var data = [
	{
		value : 30,
		color: "#D97041"
	},
	{
		value : 90,
		color: "#C7604C"
	},
	{
		value : 24,
		color: "#21323D"
	},
	{
		value : 58,
		color: "#9D9B7F"
	},
	{
		value : 82,
		color: "#7D4F6D"
	},
	{
		value : 8,
		color: "#584A5E"
	}
]
```

As you can see, for the chart data you pass in an array of objects, with a value and a colour. The `value` attribute should be a number, while the `color` attribute should be a string. Similar to CSS, for this string you can use HEX notation, RGB, RGBA or HSL.

## Chart options

These are the default chart options. By passing in an object with any of these attributes, Chart.js will merge these objects and the graph accordingly. Explanations of each option are commented in the code below.

```
PolarArea.defaults = {
				
	//Boolean - Whether we show the scale above or below the chart segments
	scaleOverlay : true,
	
	//Boolean - If we want to override with a hard coded scale
	scaleOverride : false,
	
	//** Required if scaleOverride is true **
	//Number - The number of steps in a hard coded scale
	scaleSteps : null,
	//Number - The value jump in the hard coded scale
	scaleStepWidth : null,
	//Number - The centre starting value
	scaleStartValue : null,
	
	//Boolean - Show line for each value in the scale
	scaleShowLine : true,
	
	//String - The colour of the scale line
	scaleLineColor : "rgba(0,0,0,.1)",
	
	//Number - The width of the line - in pixels
	scaleLineWidth : 1,
	
	//Boolean - whether we should show text labels
	scaleShowLabels : true,
	
	//Interpolated JS string - can access value
	scaleLabel : "<%=value%>",
	
	//String - Scale label font declaration for the scale label
	scaleFontFamily : "'Arial'",
	
	//Number - Scale label font size in pixels	
	scaleFontSize : 12,
	
	//String - Scale label font weight style	
	scaleFontStyle : "normal",
	
	//String - Scale label font colour	
	scaleFontColor : "#666",
	
	//Boolean - Show a backdrop to the scale label
	scaleShowLabelBackdrop : true,
	
	//String - The colour of the label backdrop	
	scaleBackdropColor : "rgba(255,255,255,0.75)",
	
	//Number - The backdrop padding above & below the label in pixels
	scaleBackdropPaddingY : 2,
	
	//Number - The backdrop padding to the side of the label in pixels	
	scaleBackdropPaddingX : 2,

	//Boolean - Stroke a line around each segment in the chart
	segmentShowStroke : true,
	
	//String - The colour of the stroke on each segement.
	segmentStrokeColor : "#fff",
	
	//Number - The width of the stroke value in pixels	
	segmentStrokeWidth : 2,
	
	//Boolean - Whether to animate the chart or not
	animation : true,
	
	//Number - Amount of animation steps
	animationSteps : 100,
	
	//String - Animation easing effect.
	animationEasing : "easeOutBounce",

	//Boolean - Whether to animate the rotation of the chart
	animateRotate : true,
	
	//Boolean - Whether to animate scaling the chart from the centre
	animateScale : false,

	//Function - This will fire when the animation of the chart is complete.
	onAnimationComplete : null
}
```

# Pie chart

## Introduction

Pie charts are probably the most commonly used chart there are. They are divided into segments, the arc of each segment shows a the proportional value of each piece of data.

They are excellent at showing the relational proportions between data.

## Example usage

```
new Chart(ctx).Pie(data,options);
```

## Data structure

```
var data = [
	{
		value: 30,
		color:"#F38630"
	},
	{
		value : 50,
		color : "#E0E4CC"
	},
	{
		value : 100,
		color : "#69D2E7"
	}			
]
```

For a pie chart, you must pass in an array of objects with a `value` and a `color` property. The `value` attribute should be a number, Chart.js will total all of the numbers and calculate the relative proportion of each. The `color` attribute should be a string. Similar to CSS, for this string you can use HEX notation, RGB, RGBA or HSL.

## Chart options

These are the default options for the Pie chart. Pass in an object with any of these attributes to override them.

```
Pie.defaults = {
	//Boolean - Whether we should show a stroke on each segment
	segmentShowStroke : true,
	
	//String - The colour of each segment stroke
	segmentStrokeColor : "#fff",
	
	//Number - The width of each segment stroke
	segmentStrokeWidth : 2,
	
	//Boolean - Whether we should animate the chart	
	animation : true,
	
	//Number - Amount of animation steps
	animationSteps : 100,
	
	//String - Animation easing effect
	animationEasing : "easeOutBounce",
	
	//Boolean - Whether we animate the rotation of the Pie
	animateRotate : true,

	//Boolean - Whether we animate scaling the Pie from the centre
	animateScale : false,
	
	//Function - Will fire on animation completion.
	onAnimationComplete : null
}
```

# Doughnut chart

## Introduction

Doughnut charts are similar to pie charts, however they have the centre cut out, and are therefore shaped more like a doughnut than a pie!

They are aso excellent at showing the relational proportions between data.

## Example usage

```
new Chart(ctx).Doughnut(data,options);
```

## Data structure

```
var data = [
	{
		value: 30,
		color:"#F7464A"
	},
	{
		value : 50,
		color : "#E2EAE9"
	},
	{
		value : 100,
		color : "#D4CCC5"
	},
	{
		value : 40,
		color : "#949FB1"
	},
	{
		value : 120,
		color : "#4D5360"
	}

]
```

For a doughnut chart, you must pass in an array of objects with a `value` and a `color` property. The `value` attribute should be a number, Chart.js will total all of the numbers and calculate the relative proportion of each. The `color` attribute should be a string. Similar to CSS, for this string you can use HEX notation, RGB, RGBA or HSL.

## Chart options

These are the default options for the doughnut chart. Pass in an object with any of these attributes to override them.

```
Doughnut.defaults = {
	//Boolean - Whether we should show a stroke on each segment
	segmentShowStroke : true,
	
	//String - The colour of each segment stroke
	segmentStrokeColor : "#fff",
	
	//Number - The width of each segment stroke
	segmentStrokeWidth : 2,
	
	//The percentage of the chart that we cut out of the middle.
	percentageInnerCutout : 50,
	
	//Boolean - Whether we should animate the chart	
	animation : true,
	
	//Number - Amount of animation steps
	animationSteps : 100,
	
	//String - Animation easing effect
	animationEasing : "easeOutBounce",
	
	//Boolean - Whether we animate the rotation of the Doughnut
	animateRotate : true,

	//Boolean - Whether we animate scaling the Doughnut from the centre
	animateScale : false,
	
	//Function - Will fire on animation completion.
	onAnimationComplete : null
}
```

# General issues

## Chart interactivity

If you are looking to add interaction as a layer to charts, Chart.js is **not the library for you**. A better option would be using SVG, as this will let you attach event listeners to any of the elements in the chart, as these are all DOM nodes.

Chart.js uses the canvas element, which is a single DOM node, similar in characteristics to a static image. This does mean that it has a wider scope for compatibility, and less memory implications than SVG based charting solutions. The canvas element also allows for saving the contents as a base 64 string, allowing saving the chart as an image.

In SVG, all of the lines, data points and everything you see is a DOM node. As a result of this, complex charts with a lot of intricacies, or many charts on the page will often see dips in performance when scrolling or generating the chart, especially when there are multiple on the page. SVG also has relatively poor mobile support, with Android not supporting SVG at all before version 3.0, and iOS before 5.0. (caniuse.com/svg-html5).

## Browser support

Browser support for the canvas element is available in all modern & major mobile browsers (caniuse.com/canvas).

For IE8 & below, I would recommend using the polyfill ExplorerCanvas - available at https://code.google.com/p/explorercanvas/. It falls back to Internet explorer's format VML when canvas support is not available. Example use:

```
<head>
	<!--[if lte IE 8]>
		<script src="excanvas.js"></script>
	<![endif]-->
</head>
```

Usually I would recommend feature detection to choose whether or not to load a polyfill, rather than IE conditional comments, however in this case, VML is a Microsoft proprietary format, so it will only work in IE.

Some important points to note in my experience using ExplorerCanvas as a fallback.

- Initialise charts on load rather than DOMContentReady when using the library, as sometimes a race condition will occur, and it will result in an error when trying to get the 2d context of a canvas.
- New VML DOM elements are being created for each animation frame and there is no hardware acceleration. As a result animation is usually slow and jerky, with flashing text. It is a good idea to dynamically turn off animation based on canvas support. I recommend using the excellent Modernizr to do this.
- When declaring fonts, the library explorercanvas requires the font name to be in single quotes inside the string. For example, instead of your scaleFontFamily property being simply "Arial", explorercanvas support, use "'Arial'" instead. Chart.js does this for default values.

## Bugs & issues

Please report these on the Github page - at github.com/nnnick/Chart.js.

New contributions to the library are welcome.

## License

Chart.js is open source and available under the MIT license.
