# Supplementary material for: Constructing marine expert management knowledge graph based on Trellisnet-CRF
Source: PeerJ Comput Sci. 2022 Sep 5;8:e1083. doi: 10.7717/peerj-cs.1083 (PMC9455288; doi:10.7717/peerj-cs.1083)
Supplement: Supplemental Information 3 [file peerj-cs-08-1083-s003.zip › kgocean/static/assets/chart-master/site/index.html]

Chart.js | HTML5 Charts for your website.


# Chart.js

## Easy, object oriented client side graphs for designers and developers


DocumentationDownload

### 6 Chart types

Visualise your data in different ways. Each of them animated, fully customisable and look great, even on retina displays.

### HTML5 Based

Chart.js uses the HTML5 canvas element. It supports all modern browsers, and polyfills provide support for IE7/8.

### Simple and flexible

Chart.js is dependency free, lightweight (4.5k when minified and gzipped) and offers loads of customisation options.

## Line charts

Line graphs are probably the most widely used graph for showing trends.

Chart.js has a ton of customisation features for line graphs, along with support for multiple datasets to be plotted on one chart.

## Bar charts

Bar graphs are also great at showing trend data.

Chart.js supports bar charts with a load of custom styles and the ability to show multiple bars for each x value.

## Radar charts

Radar charts are good for comparing a selection of different pieces of data.

Chart.js supports multiple data sets plotted on the same radar chart. It also supports all of the customisation and animation options you'd expect.

## Pie charts

Pie charts are great at comparing proportions within a single data set.

Chart.js shows animated pie charts with customisable colours, strokes, animation easing and effects.

## Polar area charts

Polar area charts are similar to pie charts, but the variable isn't the circumference of the segment, but the radius of it.

Chart.js delivers animated polar area charts with custom coloured segments, along with customisable scales and animation.

## Doughnut charts

Similar to pie charts, doughnut charts are great for showing proportional data.

Chart.js offers the same customisation options as for pie charts, but with a custom sized inner cutout to turn your pies into doughnuts.

### Like what you see? Download Chart.js on Github or read detailed documentation

A project by Nick Downie
