# Supplementary material for: Constructing marine expert management knowledge graph based on Trellisnet-CRF
Source: PeerJ Comput Sci. 2022 Sep 5;8:e1083. doi: 10.7717/peerj-cs.1083 (PMC9455288; doi:10.7717/peerj-cs.1083)
Supplement: Supplemental Information 3 [file peerj-cs-08-1083-s003.zip › kgocean/static/assets/fullcalendar/demos/external-dragging.html]

#### Draggable Events

My Event 1

My Event 2

My Event 3

My Event 4

My Event 5

remove after drop
