# Supplementary material for: Constructing marine expert management knowledge graph based on Trellisnet-CRF
Source: PeerJ Comput Sci. 2022 Sep 5;8:e1083. doi: 10.7717/peerj-cs.1083 (PMC9455288; doi:10.7717/peerj-cs.1083)
Supplement: Supplemental Information 3 [file peerj-cs-08-1083-s003.zip › kgocean/static/assets/jquery-easy-pie-chart/examples/index.html]

Easy Pie Chart


# EASY PIE CHART

55%

New visits

46%

Bounce rate

92%

Server load

752MB

Used RAM

55%

New visits

46%

Bounce rate

92%

Server load

752MB

Used RAM

Update pie charts

Inspired by: Simple Pie Charts II by Rafal Bromirski on dribble
