# Supplementary material for: Constructing marine expert management knowledge graph based on Trellisnet-CRF
Source: PeerJ Comput Sci. 2022 Sep 5;8:e1083. doi: 10.7717/peerj-cs.1083 (PMC9455288; doi:10.7717/peerj-cs.1083)
Supplement: Supplemental Information 3 [file peerj-cs-08-1083-s003.zip › kgocean/static/assets/jquery-knob/index.html]

jQuery Knob demo


# jQuery Knob

Nice, downward compatible, touchable, jQuery dial.

\* implemented interactions : mouse click and wheel mouse, keyboard (on focus) and fingers (touch events)

× Disable display input

```
data-width="100"
data-displayInput=false
```

× 'cursor' mode

```
data-width="150"
data-cursor=true
data-thickness=.3
data-fgColor="#222222"
```

× Display previous value

```
data-displayPrevious=true
data-min="-100"
```

× Angle offset

```
data-angleOffset=90
```

× Angle offset and arc

```
data-fgColor="#66CC66"
data-angleOffset=-125
data-angleArc=250
```

× 5-digit values

```
data-min="-15000"
data-max="15000"
```

× Overloaded 'draw' method

```
    data-width="75"
    data-fgColor="#ffec03"
    data-skin="tron"
    data-thickness=".2"
    data-displayPrevious=true
```

```
    data-width="150"
    data-fgColor="#ffec03"
    data-skin="tron"
    data-thickness=".2"
    data-displayPrevious=true
```

```
    data-width="150"
    data-fgColor="#C0ffff"
    data-skin="tron"
    data-thickness=".1"
    data-angleOffset="180"
```

× Readonly

```
data-thickness=".4"
data-fgColor="chartreuse"
data-readOnly=true
```

× Dynamic

```
data-width="200"
```


```
data-width="50"
data-cursor=true
```

× Infinite || iPod click wheel

```
data-width="150"
data-cursor=true
data-thickness=".5"
data-fgColor="#AAAAAA"
data-bgColor="#FFFFFF"
data-displayInput="false"
+ some code
```

0

× Big !

```
data-width="700"
```

jQuery Knob is © 2012 Anthony Terrien and dual licensed under the MIT or GPL licenses.
