# Supplementary material for: Constructing marine expert management knowledge graph based on Trellisnet-CRF
Source: PeerJ Comput Sci. 2022 Sep 5;8:e1083. doi: 10.7717/peerj-cs.1083 (PMC9455288; doi:10.7717/peerj-cs.1083)
Supplement: Supplemental Information 3 [file peerj-cs-08-1083-s003.zip › kgocean/templates/ent.html]

{% extends "navigate.html" %} {% block mainbody %}

#### 实体识别

1. 主页
2. 实体识别

输入文本（300字以内） :

{% csrf\_token %}

确认

实体识别结果>>

{% autoescape off %}

##### {{ rlt }}

{% endautoescape %}

中文分词结果>>

{% autoescape off %}

##### {{ seg\_word }}

{% endautoescape %}

{% endblock %}
