# Supplementary material for: Constructing marine expert management knowledge graph based on Trellisnet-CRF
Source: PeerJ Comput Sci. 2022 Sep 5;8:e1083. doi: 10.7717/peerj-cs.1083 (PMC9455288; doi:10.7717/peerj-cs.1083)
Supplement: Supplemental Information 3 [file peerj-cs-08-1083-s003.zip › kgocean/templates/entity.html]

{% extends "navigate.html" %} {% block mainbody %}


专家查询

### 专家查询

1. 主页
2. {{ user\_text }}图谱

{% if ctx %}

## 数据库中暂未添加该实体

{% endif %}

{% if entityRelation %}

关系图 :

{% endif %}
{% if cooRelation %}

相关人员关系图 :

{% endif %}
{% if paperRelation %}

论文关系图 :

{% endif %}
{% if entityRelation %}

关系列表 :

{% endif %}

{% if entityRelation %}
{% endif %}
{% endblock %}
