# Supplementary material for: Constructing marine expert management knowledge graph based on Trellisnet-CRF
Source: PeerJ Comput Sci. 2022 Sep 5;8:e1083. doi: 10.7717/peerj-cs.1083 (PMC9455288; doi:10.7717/peerj-cs.1083)
Supplement: Supplemental Information 3 [file peerj-cs-08-1083-s003.zip › kgocean/templates/index.html]

{% extends "navigate.html" %} {% block mainbody %}

查询

{% endblock %}
