# Supplementary material for: Constructing marine expert management knowledge graph based on Trellisnet-CRF
Source: PeerJ Comput Sci. 2022 Sep 5;8:e1083. doi: 10.7717/peerj-cs.1083 (PMC9455288; doi:10.7717/peerj-cs.1083)
Supplement: Supplemental Information 3 [file peerj-cs-08-1083-s003.zip › kgocean/templates/relation.html]

{% extends "navigate.html" %} {% block mainbody %}


关系

### 关系查询

1. 主页
2. 关系查询

查询条件：

关系类型

- 地域
- 研究领域
- 机构
- 合作作者
- 出版文献
- 其他

Search

{% if ctx %}

查询结果：

## 暂未找到相应的匹配

{% endif %}
{% if searchResult %}

关系图 :

{# #}
{#

#}
{#

#}
{# #}
{# 关系列表 :#}
{# #}
{#

#}
{#


#}
{#

#}
{#

#}
{#

#}
{% endif %}

{% if searchResult %}
{% endif %}
{% endblock %}
