# Supplementary figures and images for: Constructing marine expert management knowledge graph based on Trellisnet-CRF
Source: PeerJ Comput Sci. 2022 Sep 5;8:e1083. doi: 10.7717/peerj-cs.1083 (PMC9455288; doi:10.7717/peerj-cs.1083)

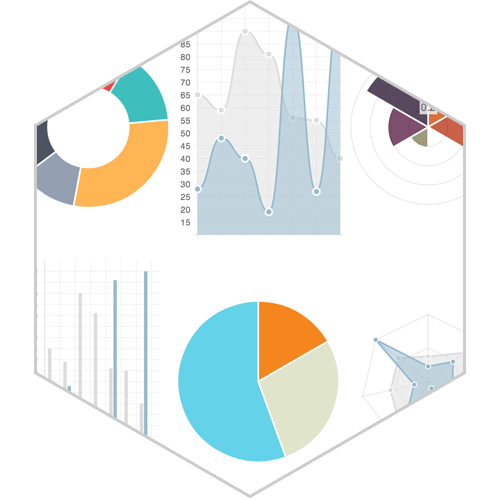

Supplement: Supplemental Information 3 [file peerj-cs-08-1083-s003.zip › kgocean/static/assets/chart-master/site/assets/6charts.png]

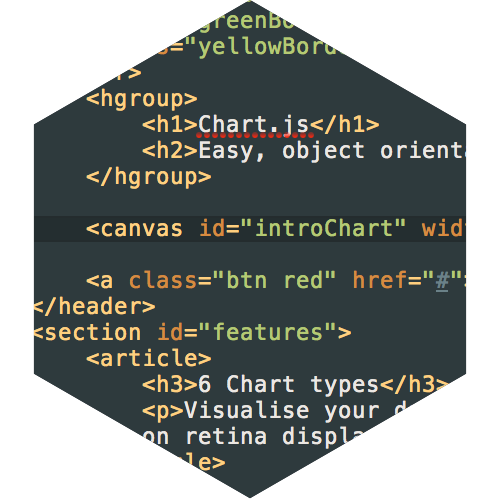

Supplement: Supplemental Information 3 [file peerj-cs-08-1083-s003.zip › kgocean/static/assets/chart-master/site/assets/html.png]

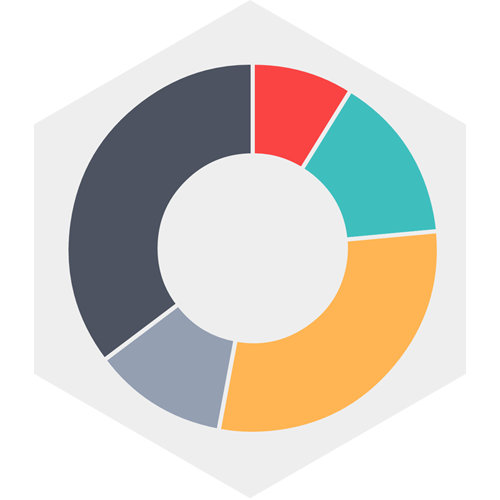

Supplement: Supplemental Information 3 [file peerj-cs-08-1083-s003.zip › kgocean/static/assets/chart-master/site/assets/simple.png]

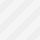

Supplement: Supplemental Information 3 [file peerj-cs-08-1083-s003.zip › kgocean/static/assets/fullcalendar/demos/cupertino/images/ui-bg_diagonals-thick_90_eeeeee_40x40.png]

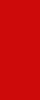

Supplement: Supplemental Information 3 [file peerj-cs-08-1083-s003.zip › kgocean/static/assets/fullcalendar/demos/cupertino/images/ui-bg_flat_15_cd0a0a_40x100.png]

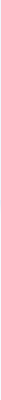

Supplement: Supplemental Information 3 [file peerj-cs-08-1083-s003.zip › kgocean/static/assets/fullcalendar/demos/cupertino/images/ui-bg_glass_100_e4f1fb_1x400.png]

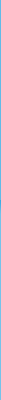

Supplement: Supplemental Information 3 [file peerj-cs-08-1083-s003.zip › kgocean/static/assets/fullcalendar/demos/cupertino/images/ui-bg_glass_50_3baae3_1x400.png]

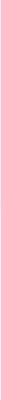

Supplement: Supplemental Information 3 [file peerj-cs-08-1083-s003.zip › kgocean/static/assets/fullcalendar/demos/cupertino/images/ui-bg_glass_80_d7ebf9_1x400.png]

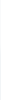

Supplement: Supplemental Information 3 [file peerj-cs-08-1083-s003.zip › kgocean/static/assets/fullcalendar/demos/cupertino/images/ui-bg_highlight-hard_100_f2f5f7_1x100.png]

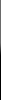

Supplement: Supplemental Information 3 [file peerj-cs-08-1083-s003.zip › kgocean/static/assets/fullcalendar/demos/cupertino/images/ui-bg_highlight-hard_70_000000_1x100.png]

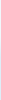

Supplement: Supplemental Information 3 [file peerj-cs-08-1083-s003.zip › kgocean/static/assets/fullcalendar/demos/cupertino/images/ui-bg_highlight-soft_100_deedf7_1x100.png]

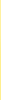

Supplement: Supplemental Information 3 [file peerj-cs-08-1083-s003.zip › kgocean/static/assets/fullcalendar/demos/cupertino/images/ui-bg_highlight-soft_25_ffef8f_1x100.png]

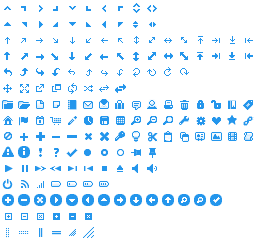

Supplement: Supplemental Information 3 [file peerj-cs-08-1083-s003.zip › kgocean/static/assets/fullcalendar/demos/cupertino/images/ui-icons_2694e8_256x240.png]

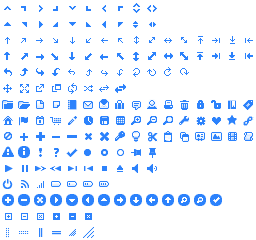

Supplement: Supplemental Information 3 [file peerj-cs-08-1083-s003.zip › kgocean/static/assets/fullcalendar/demos/cupertino/images/ui-icons_2e83ff_256x240.png]

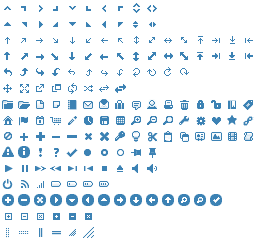

Supplement: Supplemental Information 3 [file peerj-cs-08-1083-s003.zip › kgocean/static/assets/fullcalendar/demos/cupertino/images/ui-icons_3d80b3_256x240.png]

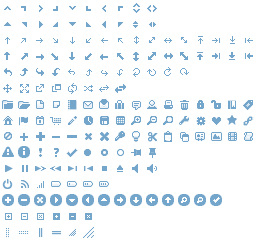

Supplement: Supplemental Information 3 [file peerj-cs-08-1083-s003.zip › kgocean/static/assets/fullcalendar/demos/cupertino/images/ui-icons_72a7cf_256x240.png]

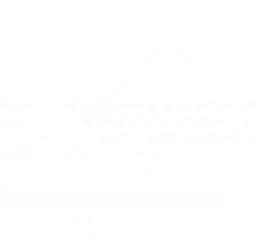

Supplement: Supplemental Information 3 [file peerj-cs-08-1083-s003.zip › kgocean/static/assets/fullcalendar/demos/cupertino/images/ui-icons_ffffff_256x240.png]

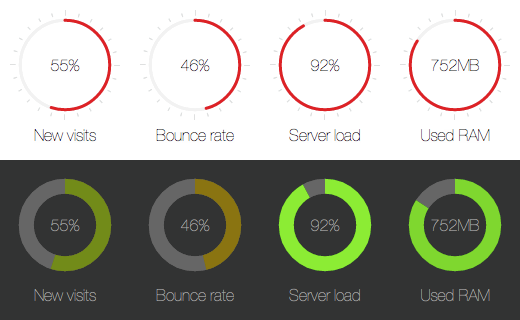

Supplement: Supplemental Information 3 [file peerj-cs-08-1083-s003.zip › kgocean/static/assets/jquery-easy-pie-chart/img/easy-pie-chart.png]

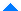

Supplement: Supplemental Information 3 [file peerj-cs-08-1083-s003.zip › kgocean/static/img/arrow-up.png]

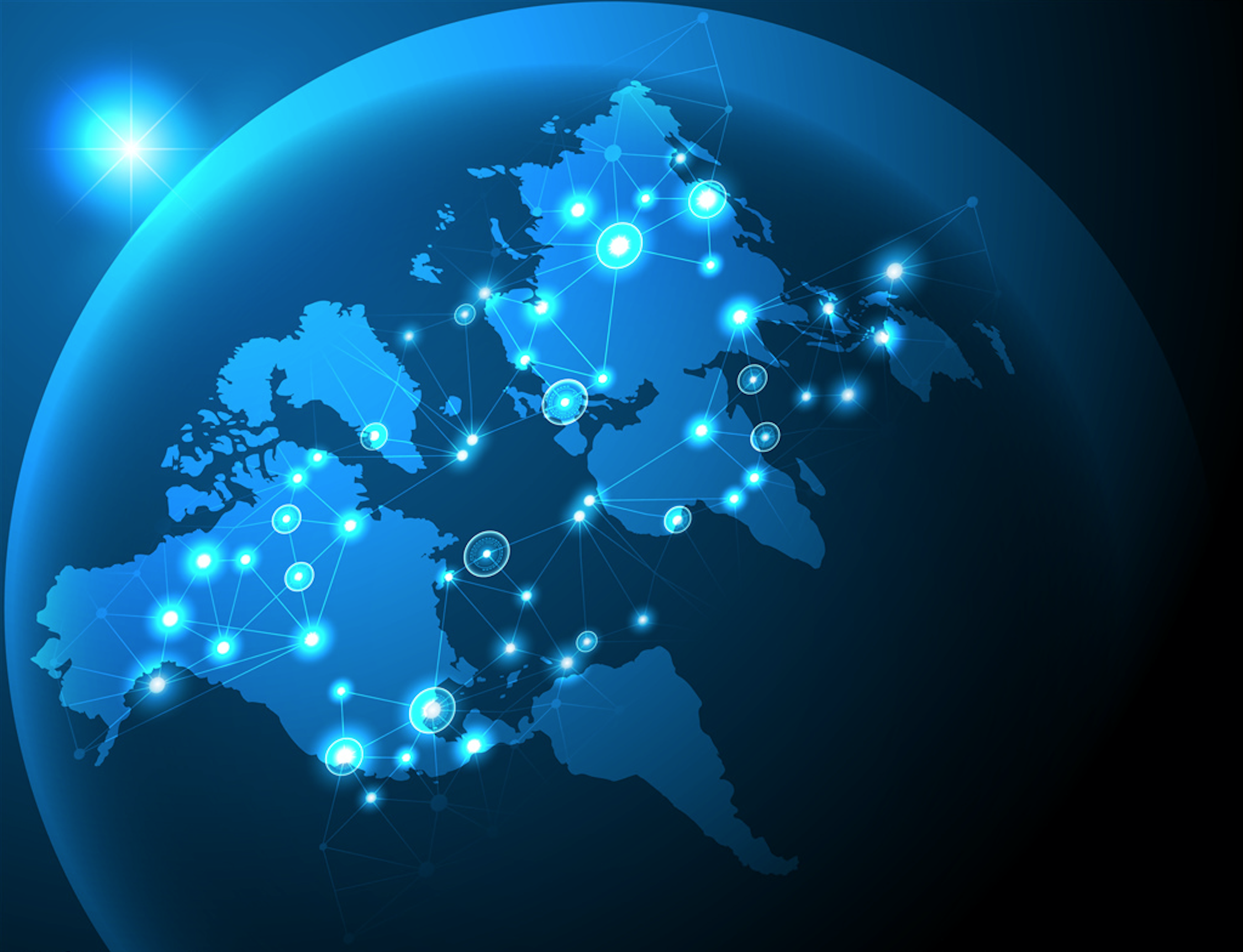

Supplement: Supplemental Information 3 [file peerj-cs-08-1083-s003.zip › kgocean/static/img/bg.png]

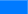

Supplement: Supplemental Information 3 [file peerj-cs-08-1083-s003.zip › kgocean/static/img/chart-texture.jpg]

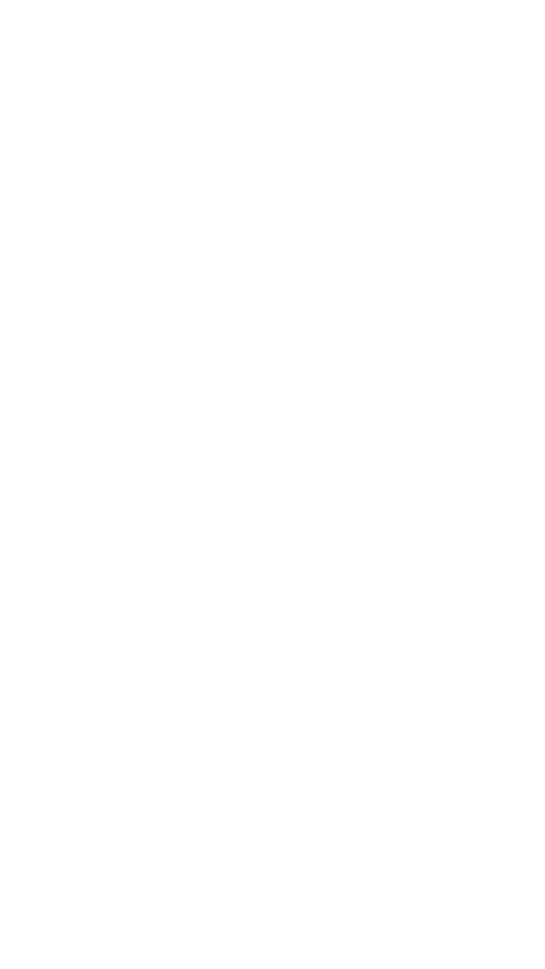

Supplement: Supplemental Information 3 [file peerj-cs-08-1083-s003.zip › kgocean/static/img/icons/line-icon-c.png]

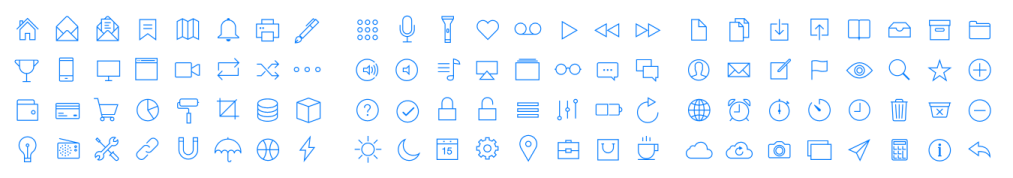

Supplement: Supplemental Information 3 [file peerj-cs-08-1083-s003.zip › kgocean/static/img/icons/line-icon-hover.png]

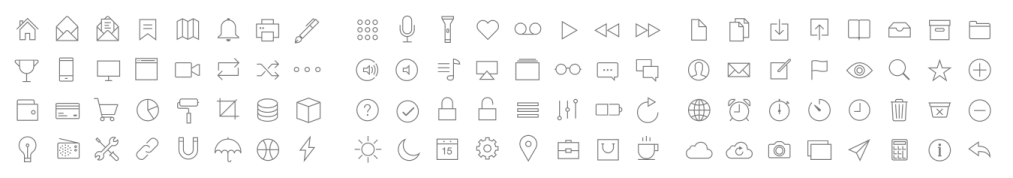

Supplement: Supplemental Information 3 [file peerj-cs-08-1083-s003.zip › kgocean/static/img/icons/line-icon.png]

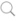

Supplement: Supplemental Information 3 [file peerj-cs-08-1083-s003.zip › kgocean/static/img/icons/search-line-icon.png]

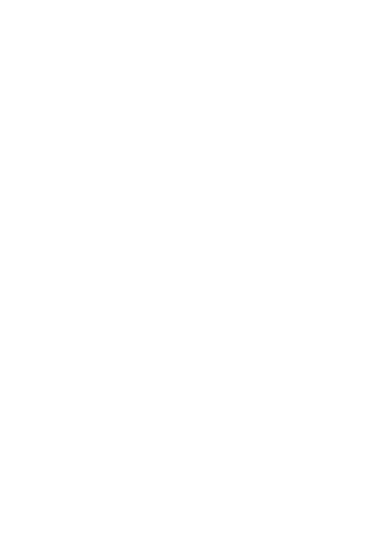

Supplement: Supplemental Information 3 [file peerj-cs-08-1083-s003.zip › kgocean/static/img/icons/social.png]

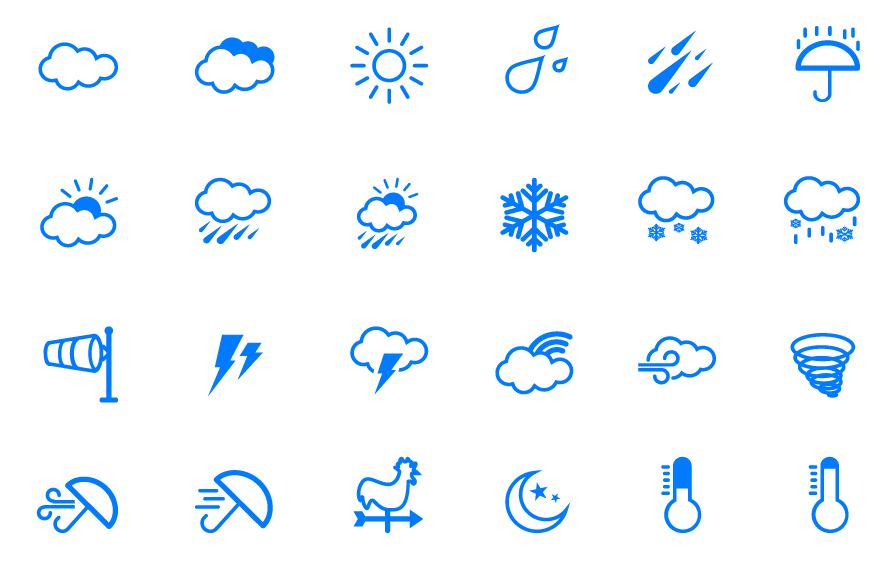

Supplement: Supplemental Information 3 [file peerj-cs-08-1083-s003.zip › kgocean/static/img/icons/weather-hover.png]

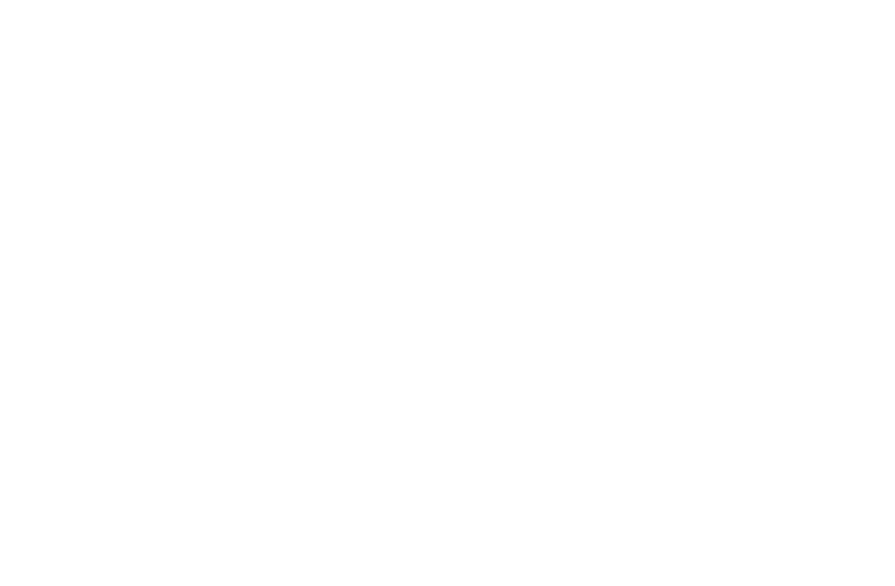

Supplement: Supplemental Information 3 [file peerj-cs-08-1083-s003.zip › kgocean/static/img/icons/weather.png]

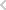

Supplement: Supplemental Information 3 [file peerj-cs-08-1083-s003.zip › kgocean/static/img/left-arrow.png]

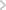

Supplement: Supplemental Information 3 [file peerj-cs-08-1083-s003.zip › kgocean/static/img/right-arrow.png]
