# Supplementary material for: Constructing marine expert management knowledge graph based on Trellisnet-CRF
Source: PeerJ Comput Sci. 2022 Sep 5;8:e1083. doi: 10.7717/peerj-cs.1083 (PMC9455288; doi:10.7717/peerj-cs.1083)
Supplement: Supplemental Information 2 [file peerj-cs-08-1083-s002.zip › Peopleí»s Daily Corpus-raw data/corpus_Guideline/Guideline_appendix_Chinese.doc]

**附录1 按代码的字母顺序排列的标记集**

代码	名称	帮助记忆的诠释

Ag	形语素	形容词性语素。形容词代码为a，语素代码ｇ前面置以A。

a	形容词	取英语形容词adjective的第1个字母。

ad	副形词	直接作状语的形容词。形容词代码a和副词代码d并在一起。

an	名形词	具有名词功能的形容词。形容词代码a和名词代码n并在一起。

Bg

b	区别词	取汉字“别”的声母。

c	连词	取英语连词conjunction的第1个字母。

Dg	副语素	副词性语素。副词代码为d，语素代码ｇ前面置以D。

d	副词	取adverb的第2个字母，因其第1个字母已用于形容词。

e	叹词	取英语叹词exclamation的第1个字母。

f	方位词	取汉字“方” 的声母。

g	语素	绝大多数语素都能作为合成词的“词根”，取汉字“根”的声母。由于实际标注时，一定标注其子类，所以从来没有用到过g。

h	前接成分	取英语head的第1个字母。

i	成语	取英语成语idiom的第1个字母。

j	简称略语	取汉字“简”的声母。

k	后接成分

l	习用语	习用语尚未成为成语，有点“临时性”，取“临”的声母。

Mg

m	数词	取英语numeral的第3个字母，n，u已有他用。

Ng	名语素	名词性语素。名词代码为n，语素代码ｇ前面置以N。

n	名词	取英语名词noun的第1个字母。

nr	人名	名词代码n和“人(ren)”的声母并在一起。

ns	地名	名词代码n和处所词代码s并在一起。

nt	机构团体	“团”的声母为t，名词代码n和t并在一起。

nx

nz	其他专名	“专”的声母的第1个字母为z，名词代码n和z并在一起。

o	拟声词	取英语拟声词onomatopoeia的第1个字母。

p	介词	取英语介词prepositional的第1个字母。

Qg

q	量词	取英语quantity的第1个字母。

Rg

r	代词	取英语代词pronoun的第2个字母,因p已用于介词。

s	处所词	取英语space的第1个字母。

Tg	时语素	时间词性语素。时间词代码为t,在语素的代码g前面置以T。

t	时间词	取英语time的第1个字母。

Ug

u	助词	取英语助词auxiliary 的第2个字母,因a已用于形容词。

Vg	动语素	动词性语素。动词代码为v。在语素的代码g前面置以V。

22

v	动词	取英语动词verb的第一个字母。

vd	副动词	直接作状语的动词。动词和副词的代码并在一起。

vn	名动词	指具有名词功能的动词。动词和名词的代码并在一起。

w	标点符号

x	非语素字	非语素字只是一个符号，字母x通常用于代表未知数、符号。

Yg

y	语气词	取汉字“语”的声母。

z	状态词	取汉字“状”的声母的前一个字母。

说明：在《人民日报》前6个月的标注语料中，还出现了如下标记：Bg（区别语素）, Mg（数语素）, Qg（量语素）, Rg（代语素）, Ug（助语素）, Yg（语气语素）。这些语素的出现是否合理，尚需斟酌。由于它们出现的次数极少，不会影响语料库的使用。北大计算语言学研究所将尽快研究，给出一个合理的结论。在附录2中暂不列这些标记。

23

**附录2 按名称的汉语拼音顺序排列的标记集**

名称	代码	拼音

标点符号 w	biao1dian3fu2hao4

成语	i	cheng2yu3

处所词	s	chu4suo3ci2

代词	r	dai4ci2

地名	ns	di4ming2

动词	v	dong4ci2

动语素	Vg	dong4yu3su4

方位词	f	fang1wei4ci2

非语素字 x	fei1yu3su4zi4

副词	d	fu4ci2

副动词	vd	fu4dong4ci2

副形词	ad	fu4xing2ci2

副语素	Dg	fu4yu3su4

后接成分 k	hou4jie1cheng2fen4

机构团体 nt	ji1gou4tuan2ti3

简称略语 j	jian3cheng1lve4yu3

介词	p	jie4ci2

连词	c	lian2ci2

量词	q	liang4ci2

名词	n	ming2ci2

名动词	vn	ming2dong4ci2

名形词	an	ming2xing2ci2

名语素	Ng	ming2yu3su4

拟声词	o	ni2sheng1ci2

其他专名 nz	qi2ta1zhuan1ming2

前接成分 h	qian2jie1cheng2fen4

区别词	b	qu1bie2ci2

人名	nr	ren2ming2

时间词	t	shi2jian1ci2

时语素	Tg	shi2yu3su4

数词	m	shu4ci2

叹词	e	tan4ci2

习用语	l	xi1yong4yu3

形容词	a	xing2rong2ci2

形语素	Ag	xing2yu3su4

语气词	y	yu3qi4ci2

语素	g	yu3su4

助词	u	zhu4ci2

状态词	z	zhuang4tai4ci2

24

**附录3 参考文献**

1 中国国家标准GB13715《信息处理用现代汉语分词规范》，见刘源等著《信息处理用现代

汉语分词规范及自动分词方法》，北京：清华大学出版社，1994年第1版。

2 陆志韦等，《汉语的构词法》，科学出版社，1964年。
3 俞士汶、朱学锋、王惠，《现代汉语语法信息词典》的新进展，《中文信息学报》, 2001，

15卷1期, 59-65（封三）

4 段慧明、松井久仁於、徐国伟、胡国昕、俞士汶，大规模汉语标注语料库的制作与使用，

《语言文字应用》，2000年2期，72-77

5 俞士汶、朱学锋、段慧明，大规模现代汉语标注语料库的加工规范，《中文信息学报》，

2000年第6期，58-64

6 俞士汶，朱学锋，王惠，张芸芸，《现代汉语语法信息词典详解》，北京：清华大学出版社

1998年第1版。

7	朱学锋，俞士汶，王惠，现代汉语5万词语归类的实践，《语言文字应用》，1997年第4期，

88—94

8	周强，俞士汶，一种切词和词性标注相融合的汉语语料库多级加工方法，

见：陈力为主编《计算机研究与运用》，北京语言学院出版社，126--131。1993

9	周强，段慧明，现代汉语语料库加工中的切词与词性标注处理，《中国计算机报》，

1994年5月31日，第85版。

10 周强，张伟，俞士汶，树库的构建，《中文信息学报》，1997年第4期，42—51

11 朱德熙，《现代汉语语法研究》，北京：商务印书馆，1980

25
