# Supplementary material for: Constructing marine expert management knowledge graph based on Trellisnet-CRF
Source: PeerJ Comput Sci. 2022 Sep 5;8:e1083. doi: 10.7717/peerj-cs.1083 (PMC9455288; doi:10.7717/peerj-cs.1083)
Supplement: Supplemental Information 2 [file peerj-cs-08-1083-s002.zip › Peopleí»s Daily Corpus-raw data/corpus_Guideline/Guideline_Chinese.doc]

现代汉语语料库加工规范

——词语切分与词性标注

**⒈ 前言**

北大计算语言学研究所从1992年开始进行汉语语料库的多级加工研究。第一步是对原始语料进行切分和词性标注。1994年制订了《现代汉语文本切分与词性标注规范Ｖ1.0》。几年来已完成了约60万字语料的切分与标注，并在短语自动识别、树库构建等方向上进行了探索。在积累了长期的实践经验之后，最近又进行了《人民日报》语料加工的实验。为了保证大规模语料加工这一项重要的语言工程的顺利进行，北大计算语言学研究所于1998年10月制订了《现代汉语文本切分与词性标注规范Ｖ2.0》（征求意见稿）。因这次加工的任务超出词语切分与词性标注的范围，但重心不变，为了便于今后的发展（制订系列规范），故将新版的规范改名为《现代汉语语料库加工规范——词语切分与词性标注（1999年3月版）》。现在，北大计算语言学研究所已完成1998年《人民日报》前8个月的语料的加工任务，并交付Fujitsu 验收和使用。通过两年多的实践，对《规范》有了一些新的认识。也增加了一些标记。现在将《规范》中的疏漏加以订正和补充，形成2001年7月版。2001年8月公开前6个月的加工好了的语料库（以下简称“标注语料库”）时，《现代汉语语料库加工规范——词语切分与词性标注（2001年7月版）》是资料的重要组成部分。

制订《现代汉语语料库加工规范》的基本思路如下：

1 词语的切分规范尽可能同中国国家标准GB13715“信息处理用现代汉语分词规范” （以下简称为“分词规范”）保持一致。由于现在词语切分与词性标注是结合起来进行的，而且又有了一部《现代汉语语法信息词典》（以下有时简称“语法信息词典”或“语法词典”）可作为词语切分与词性标注的基本参照，这就有必要对“分词规范”作必要的调整和补充。
2 小标记集。词性标注除了使用《现代汉语语法信息词典》中的26个词类标记（名词n、时间词t、处所词s、方位词f、数词m、量词q、区别词b、代词r、动词v、形容词a、状态词z、副词d、介词p、连词c、助词u、语气词y、叹词e、拟声词o、成语i、习用语l、简称j、前接成分h、后接成分k、语素g、非语素字x、标点符号w）外，增加了以下3类标记：①专有名词的分类标记，即人名nr，地名ns，团体机关单位名称nt，其他专有名词nz，英语等其他外语的字母或字符串nx。②语素的子类标记，即名语素Ng，动语素Vg，形容语素Ag，时语素Tg，副语素Dg等；由于标注时实际使用各子类标记，故语素标记g 并不在标注语料库中出现。在前6个月的标注语料库中还出现了极少的其他子类的语素，其命名方式都是一样的，如量语素就是将量词代码q换成大写Q，后面再加个g，即Qg。③动词和形容词的子类标记，即名动词vn（具有名词特性的动词），名形词an（具有名词特性的形容词），副动词vd（具有副词特性的动词），副形词ad（具有副词特性的形容词）。合计40多个左右。与《现代汉语语法信息词典》结合，这个标记集是容易扩充的。
3 规范的多元性。既要适应语言信息处理与语料库语言学研究的需要，又要能为传统的语言研究提供充足的素材；既要适合计算机自动处理，又要便于人工校对。
4 词组（短语）本位汉语语法体系的指导作用。汉语的词类与句法成分之间不存在简单的一一对应关系。同一个句法成分可以由不同词性的词来充任；而具有确定词性的同一个词又可以充当不同的句法成分，形式上可以没有任何不同的标记。《现代汉语语法信息词典》是在词组本位语法体系的指导下研制的，其最基本的成果是对数以万计的词语根据其在实际语料中的语法功能分布，特别是优势功能分布决定了它们的词性（即它们所属的词类）。在进行词性标注时，应当充分利用《现代汉语语法信息词典》，承接这个基本成果，避免仅根据词在所在的句子中充当的句子成分来决定其词性。同时考虑到语言学界对汉语词类的划分存在不同意见，在标记集中增加了名动词vn，名形词an，副动词vd，副形词ad。当然，增加这些标记并非只是缓冲不同意见，主要是为了给词的兼类研究提供计量根据，也为汉语词的概率语法属性描述作准备。

⑸	为了对新闻语料中大量存在的专有名词（地名、团体机构名称等）进行研究（从命名规律到自动识别），在切分与标注的基础上对由若干个词组合而成的短语型专有名词加上方括号和类型标记（主要是nt，nz，还有少量的ns）。

⑹	不是孤立使用本规范。在使用本规范对语料库进行加工时，将与《现代汉语语法信息词典》等项研究成果密切配合。当现在的研究和将来的应用（中文信息检索、中文信息提取、汉外机器翻译等）相衔接时，就可以方便地利用词典中丰富的词语语法属性信息。而标注语料库又可以验证和丰富词典中词语的属性，进而建立词的概率语法属性描述。标注语料库同《现代汉语语法信息词典》结合，形成一个立体的语言知识库。语料库的进一步深加工（树库，注音，义项标注等）也有了可靠的基础。

⑺	人机互助的工作方式。以本规范为基准，继续扩充自动加工软件的功能并提高其精度。人工校对是必要的。需要重视校对人员之间的切磋，要将对学术问题的不同见解统一到规范的制订与执行上。开发机器辅助编辑和语料库管理软件，保证语料库加工的一致性。

本规范分为三个部分：

① 切分规范

切分规范主要规定现代汉语的切词原则，即什么样的汉字组合可以为一个切分单位。第2章中详细介绍对“分词规范”的补充与调整。

② 切分和标注相结合的规范

在汉语中，像“双音节动词＋单音节名词”通常构成新的名词，对于这个新的名词，即使在词典中没有登录，也应该把它们处理为一个切分单位。因此，在本规范中，给出了一些基于词性描述的构词规律，规定了什么样的组合可以处理为一个切分单位，并给出了新组合的词的词性标记。这部分内容主要集中在第3章中。

1. 标注规范

③-1 一般词性标注

标注规范用以确定切分单位的标记。以1999年7月份语法词典小组提供的7万词表为基本参照，其含义包括:

a. 标记集以26个词类标记为基准，名动词、副动词、名形词、副形词和专有名词的标记是在动词代码v、形容词代码a、名词代码n后增加一个小写字母，语素标记是在语素代码g前面增加一个大写字母。
b. 一个词若在语法词典中已属于某一个或若干个词类，标注时不轻易增加词性。如“训练”、“强调”在语法词典中只属于动词，标注时切勿仅根据其在当前句子的功能就将它们改为名词或副词，可以标注为名动词vn或副动词vd。
c. 当语法词典给某个词确定的词性不对或不完备时，当然也要订正或补充。这时应通知词典小组。
d. 即使语法词典中的简称实际上指的是团体、机构、组织名称或地名，标注时仍标以j，而不要改为nt或ns。
e. “唐朝”、“宋代”等历史朝代名称虽然也是专名，因语法词典已作为时间词收入，标注时仍标以t，不改为nz。

第4章集中论述标注规范。

③-2 专有名词标注

这里“专有名词”的含义有了拓展。短语型的地名、团体机构名称及其他专有名称在词的切分基础上用ASCII码的方括号括起来，并在右方括号之后标以相应的ns、nt、nz，方括号不嵌套。第2章与第3章都涉及这部分内容。

**⒉ 切分规范**

**2.1 基本概念**

⑴ 切分单位

“分词单位”是中国国家标准“分词规范”中的一个基本概念。它是指信息处理中使用的、具有确定的语义和语法功能的基本单位。为了同“分词规范”衔接，这里仍沿用“分词单位”这个概念，不过术语改用“切分单位”，因为“分词”这个术语已在英语语法中长期使用，大家已经熟悉，而用同一个术语表达同一或邻近学科的多个概念容易引起混淆。

按照“分词规范”对“切分单位”的定义和解释，本切分规范中的“切分单位”主要是词，也包括了一部分结合紧密、使用稳定的词组。在某些特殊情况下孤立的语素或非语素字也可能出现在切分序列中，如在动词的离合形式

出/v 了/u 一/m 次/q 差/Ng 。/w

中，“差/Ng”是名语素；又如在

鹧鸪/n 的/u 鹧/x 有/v 什么/r 意思/n 吗/y ？/w

中，“鹧/x”是非语素字。

**从字数考虑，对两个字的组合较宽地看作是一个切分单位，三个字的较严，四个字以上的若不是成语、习惯用语一般不看作是一个切分单位。**

⑵ 词典词条

“词典词条”（或“词条”）指《现代汉语语法信息词典》中收录的那些词语。1999年7月提供给本项工程使用的词条的总数约7.3万，这些词语都已归了类，即带有词性标记。

⑶ 切分单位和词条的关系

汉语中，词和词组、成词语素和不成词语素的边界是模糊的。本规范规定，凡收入语法信息词典的词条（包括：词、词组、成语、习用语、简称乃至标点符号等）一般都是切分单位。由于语法词典中的词条多达7.3万，对真实文本的覆盖率很高，因此可以保证绝大多数切分单位和语法词典的词条是一致的，但两者之间还是有差异的。例如5个字以上的成语、习用语是切分单位，但未被收入现在的语法词典。像“一百二十八”、“五分之三”、“百分之九”、“1998年”、“10月30日”这样的数词和时间词实际上是无限多的，语法词典不可能全收，只可能收少量的构成成分。反过来，像“分之”、“百分之”作为助数词收入了语法词典，但它们并不是切分单位。语法词典中包含的前接成分、后接成分、语素、非语素字不是切分单位，尽管当它们不能与前后成分组合时也会孤立地出现在切分序列中。

当处理大规模真实文本时，不可避免地会碰到未登录词。第3章给出了一些合成词的构造规则。根据这些规则自动生成的或经校对者确认的切分单位，如果结合稳定，使用频度较高，以后有可能补充到语法词典中。

**2.2 对分词规范的补充和调整**

为醒目起见，以下用符号“*”标识那些补充的规定，用“Δ”标识那些调整的规定。

⑴ 人名: nr

① 汉族人及与汉族起名方式相同的非汉族人的姓和名单独切分，并分别标注为nr。

张/nr 仁伟/nr， 欧阳/nr 修/nr， 阮/nr 志雄/nr， 朴/nr 贞爱/nr

* 汉族人除有单姓和复姓外，还有双姓，即有的女子出嫁后，在原来的姓上加上丈

夫的姓。如：陈方安生。这种情况切分、标注为：陈/nr 方/nr 安生/nr；

唐姜氏，切分、标注为：唐/nr 姜氏/nr。

② 姓名后的职务、职称或称呼要分开。

江/nr 主席/n，	小平/nr 同志/n，	江/nr 总书记/n，

张/nr 教授/n，	王/nr 部长/n，	陈/nr 老总/n，

李/nr 大娘/n，	刘/nr 阿姨/n，	龙/nr 姑姑/n

③ 对人的简称、尊称等若为两个字，则合为一个切分单位，并标以nr。

老张/nr， 大李/nr， 小郝/nr, 郭老/nr， 陈总/nr

④ 明显带排行的亲属称谓要切分开，分不清楚的则不切开。

三/m 哥/n，	大婶/n，	大/a 女儿/n， 大哥/n, 小弟/n, 老爸/n

* ⑤ 一些著名作者的或不易区分姓和名的笔名通常作为一个切分单位。

鲁迅/nr， 茅盾/nr， 巴金/nr， 三毛/nr， 琼瑶/nr， 白桦/nr

⑥ 外国人或少数民族的译名（包括日本人的姓名）不予切分，标注为nr。

克林顿/nr， 叶利钦/nr， 才旦卓玛/nr， 小林多喜二/nr， 北研二/nr,

华盛顿/nr， 爱因斯坦/nr

Δ 有些西方人的姓名中有小圆点，也不分开。

卡尔·马克思/nr

⑵ 地名: ns

安徽/ns，	深圳/ns， 杭州/ns， 拉萨/ns，	哈尔滨/ns， 呼和浩特/ns，

乌鲁木齐/ns， 长江/ns， 黄海/ns， 太平洋/ns， 泰山/ns， 华山/ns，

亚洲/ns, 海南岛/ns， 太湖/ns， 白洋淀/ns, 俄罗斯/ns，哈萨克斯坦/ns，

彼得堡/ns，	伏尔加格勒/ns

① 国名不论长短，作为一个切分单位。

中国/ns， 中华人民共和国/ns， 日本国/ns， 美利坚合众国/ns， 美国/ns

△ ② 地名后有“省”、“市”、“县”、“区”、“乡”、“镇”、“村”、“旗”、“州”、“都”、“府”、“道”等单字的行政区划名称时，不切分开，作为一个切分单位。

四川省/ns， 天津市/ns，景德镇市/ns， 沙市市/ns， 牡丹江市/ns，正定县/ns，海淀区/ns, 通州区/ns，东升乡/ns， 双桥镇/ns 南化村/ns，华盛顿州/ns，俄亥俄州/ns，东京都/ns， 大阪府/ns，北海道/ns， 长野县/ns，开封府/ns，宣城县/ns

△ ③ 地名后的行政区划有两个以上的汉字，则将地名同行政区划名称切开，不过要将地名同行政区划名称用方括号括起来，并标以ns。

[芜湖/ns 专区/n]ns，[宣城/ns 地区/n]ns，[内蒙古/ns 自治区/n]ns，

[宁夏/ns 回族/nz自治区/n]ns， [深圳/ns 特区/n]ns，

[厦门/ns 经济/n 特区/n]ns，	[香港/ns 特别/a 行政区/n]ns，

[香港/ns 特区/n]ns，	[华盛顿/ns 特区/n]ns,

[广西/ns 环江/ns 毛南族/nz 自治县/n]ns，

[青海/ns 果洛/ns 藏族/nz 自治州/n]ns

④ 地名后有表示地形地貌的一个字的普通名词，如“江、河、山、洋、海、岛、峰、湖”等，不予切分。

鸭绿江/ns， 亚马逊河/ns, 喜马拉雅山/ns, 珠穆朗玛峰/ns，地中海/ns， 大西洋/ns，	洞庭湖/ns，	塞普路斯岛/ns

△⑤ 地名后接的表示地形地貌的普通名词若有两个以上汉字，则应切开。也要将地名同该普通名词用方括号括起来，并标以ns。

[台湾/ns 海峡/n]ns， [华北/ns 平原/n]ns，	[帕米尔/ns 高原/n]ns，	[南沙/ns 群岛/n]ns，	[京东/ns 大/a 峡谷/n]ns [横断/b 山脉/n]ns

⑥ 地名后有表示自然区划的一个字的普通名词，如“ 街，路，道，巷，里，町，庄，村，弄，堡”等，不予切分。

中关村/ns， 长安街/ns， 学院路/ns， 景德镇/ns, 吴家堡/ns,

庞各庄/ns， 三元里/ns， 彼得堡/ns, 北菜市巷/ns,

△⑦ 地名后接的表示自然区划的普通名词若有两个以上汉字，则应切开。也要将地名同自然区划名词用方括号括起来，并标以ns。

[米市/ns 大街/n]ns, [蒋家/nz 胡同/n]ns, [陶然亭/ns 公园/n]ns

⑧ 大小地名相连时的标注方式为：

北京市/ns 海淀区/ns 海淀镇/ns [南/f 大街/n]ns [蒋家/nz 胡同/n]ns 24/m 号/q

△（3） 团体、机构、组织的专有名称: nt

① 团体、机构、组织的专有名称若作为名词登录在语法词典中，则直接标注为nt。

联合国/nt，	中共中央/nt，	国务院/nt， 北京大学/nt

② 大多数团体、机构、组织的专有名称一般是短语型的，较长，且含有地名或人名等专名，不会登录在语法词典中，本规范规定先切分，再组合，加方括号标注为nt。

[中国/ns 计算机/n 学会/n]nt，	[香港/ns 钟表业/n 总会/n]nt,

[烟台/ns 大学/n]nt, [合肥/ns 师范/n 学院/n]nt，

[北京/ns 图书馆/n]nt, [富士通/nz 株式会社/n]nt,

[香山/ns 植物园/n]nt, [安娜/nz 美容院/n]nt，

[上海/ns 手表/n 厂/n]nt，	[永和/nz 烧饼铺/n]nt，

[北京/ns 国安/nz 队/n]nt，	北京队/nt，	雷锋班/nt

**注**：“北京队”、“雷锋班”作为“切分单位”，“厂”同“手表”切开，而“铺”同“烧饼”又合在一起。这些处理，皆符合双音节名词同后面的单音节名词或语素结合的一般规则。详见第4章。

③ 团体、机构、组织名称的专指性是必要的，孤立的“大学、学院、图书馆、植物园”等只标为n，不标为nt。在一篇文章的开头，团体、机构、组织名称的专指性是明确的，后文往往使用简称。当省略了专名，只剩下普通名词时，就不再标nt。如采访浙江省委书记的报道，记者开始一定会写明“浙江省委”，这时加工成：

[浙江/ns 省委/n]nt

后文引用省委书记的话时，尽管“省委”指的就是“浙江省委”，但只标注为：

省委/n

也就是说，本次加工只考虑局部的上下文，而不作远程相关的语义分析。同样，“北京大学校长办公室”应加工为：

[北京大学/nt 校长/n 办公室/n]nt

若句子中只有“校长办公室”，前面没有“北京大学”，则只加工成：

校长/n 办公室/n

尽管在给定的更大的上下文环境中，该“校长办公室”是专指的。

④ 尽管有③的规定，对于在国际或中国范围内的知名的唯一的团体、机构、组织的名称即使前面没有专名，也标为nt。

联合国/nt， [世界/n 贸易/n 组织/n]nt，

国务院/nt， 外交部/nt， 财政部/nt， 教育部/nt, 国防部/nt，

[国家/n 教育/vn 委员会/n]nt, [信息/n 产业/n 部/n]nt,

[全国/n 信息/n 技术/n 标准化/vn 委员会/n]nt,

[全国/n 总/b 工会/n]nt, [全国/n 人民/n 代表/n 大会/n]nt

美国的“国务院”，其他国家的“外交部、财政部、教育部”，必须在其所属国的国名之后出现时，才联合标注为nt。

[美国/ns 国务院/n]nt，	[法国/ns 外交部/n]nt，	[美/j 国会/n]nt

日本有些政府机构名称很特别，无论是否出现在“日本”国名之后都标为nt。

[日本/ns 外务省/nt]nt， [日/j 通产省/nt]nt， [日本国/ns 法务省/nt]nt，

通产省/nt

⑤ 前后相连有上下位关系的团体机构组织名称的处理方式如下:

[联合国/nt 教科文/j 组织/n]nt

[中国/ns 银行/n 北京/ns 分行/n]nt

[河北省/ns 正定县/ns 西平乐乡/ns 南化村/ns 党支部/n]nt

[北京大学/nt 昌平/ns 分校/n]nt

[安徽/ns 人大/j 常委会/j 办公室/n]nt

[北京大学/nt 计算/vn 语言学/n 研究所/n]nt

当下位名称含有专名（如“北京/ns 分行/n”、“南化村/ns 党支部/n”、“昌平/ns 分校/n”）时，也可脱离前面的上位名称单独标注为nt。

[中国/ns 银行/n]nt	[北京/ns 分行/n]nt

河北省/ns 正定县/ns 西平乐乡/ns [南化村/ns 党支部/n]nt

北京大学/nt [昌平/ns 分校/n]nt

如果下位名称不含有专名，则必须同上位名称捆绑在一起标注。

⑥ 团体、机构、组织名称中用圆括号加注简称时的处理方法示例。

[宝山/ns 钢铁/n （/w 宝钢/j ）/w 总/b 公司/n]nt

[宝山/ns 钢铁/n 总/b 公司/n]nt （/w 宝钢/j ）/w

△（4） 除人名、国名、地名、团体、机构、组织以外的其他专有名词都标以nz，具体规定如下。

① 专有名称后接单音节的语素，如表示民族的“族”、表示语言的“语”，表示文字的“文”，则不切分，标注为nz。

满族/nz， 俄罗斯族/nz， 哈萨克族/nz， 塞尔维亚族/nz， 高山族/nz，

维吾尔语/nz， 蒙古语/nz， 汉语/nz， 罗马利亚语/nz， 捷克语/nz

中文/nz, 英文/nz， 西班牙文/nz，蒙文/nz，俄文/nz

② 专有名称后接单音节的名词，如表示人种的“人”、表示奖项的“奖”，通常不切分，标以nz；也允许切分，分别标注。

满人/nz， 哈萨克人/nz， 诺贝尔奖/nz， 茅盾奖/nz,

哈萨克/nz 人/n, 高山族/nz人/n, 安徽/ns 人/n

③ 包含专有名称（或简称）的交通线，标以nz；短语型的，使用方括号。

津浦路/nz，	石太线/nz，	[京/j 九/j 铁路/n]nz,

[京/j 津/j 高速/b 公路/n]nz，

[北京/ns -/w 西雅图/ns 航线/n]nz

④ 历史上重要事件、运动等专有名称一般是短语型的，按短语型专有名称处理，标以nz。

[卢沟桥/ns 事件/n]nz， [西安/ns 事变/n]nz， [五四/t 运动/n]nz

[明治/nz 维新/n]nz，	[甲午/t 战争/n]/nz

⑤ 专有名称后接多音节的名词，如“语言”、“文学”、“文化”、“方式”、“精神”等，失去专指性，则应切分。

欧洲/ns 语言/n， 法国/ns 文学/n， 西方/ns 文化/n，

贝多芬/nr 交响乐/n, 雷锋/nr 精神/n,

美国/ns 方式/n， 日本/ns 料理/n， 宋朝/t 古董/n

也有人认为“主义”是后接成分,且其后常接另一个后接成分“者”,因此将“主义”同其前面的专有名称合在一起作为一个切分单位（参见：3.2(2)③之d）。

马克思主义/n, 马克思列宁主义/n, 杜鲁门主义/n,

马克思主义者/n, 列宁主义者/n, 社会主义者/n

⑥ 商标（包括专名及后接的“牌”、“型”等）是专指的，标以nz，但其后所接的商品仍标以普通名词n。

康师傅/nr 方便面/n, 中华牌/nz 香烟/n, 牡丹III型/nz 电视机/n

联想/nz 电脑/n, 鳄鱼/nz 衬衣/n， 耐克/nz 鞋/n

⑦ 以序号命名的名称一般不认为是专有名称。

2/m 号/q 国道/n ，	十一/m 届/q 三中全会/j

如果前面有专名，合起来作为短语型专名也是可以的。

[中国/ns 101/m 国道/n]nz, [中共/j 十一/m 届/q 三中全会/j]nz

⑧ 书、报、杂志、文档、报告、协议、合同等的名称通常有书名号加以标识，不作为专有名词。由于这些名字往往较长，名字本身按常规处理。

《/w 宁波/ns 日报/n 》/w ，《/w 鲁迅/nr 全集/n 》/w,

中华/nz 读书/vn 报/n, 杜甫/nr 诗选/n，

《/w 大众/n 医学/n 》/w， 邓/nr 小平/nr 文选/n

少数收入词典的书名、报刊名等专有名称，则不切分。

红楼梦/nz，	人民日报/nz，	儒林外史/nz

⑨ 当有些专名无法分辨它们是人名还是地名或机构名时，暂标以nz。

[巴黎/ns 贝尔希/nz	体育馆/n]nt,

其中“贝尔希”只好暂标为nz。

⑩ 一般的命名活动常用引号表示，也不看作专有名称。

迎/v 香港/ns 回归/v 京九/j 植绿护绿/l 活动/vn

第三/m 次/q 横田/ns 基地/n 噪音/n 诉讼/vn

食谱上的菜名等通常也是短语型的，若拆开了，意思差别甚远，则不切分，否则切分。即使不切分，也不看作是专有名词。

宫保肉丁/n， 木樨肉/n，	松鼠鳜鱼/n, 红烧肉/n，

鸡蛋/n 汤/n， 芝麻/n 饼/n， 鸡丝/n 面/n

△（5） 数词与数量词组

① 基数、序数、小数、分数、百分数一律不予切分，为一个切分单位，标注为 m 。

一百二十三/m，	120万/m，	123.54/m, 一个/m,

第一/m，	第三十五/m，	20%/m， 三分之二/m，	千分之三十/m

“几”和“零”属于基本的系数词（或位数词），因此包含“几”和“零”的基数、序数、小数、分数、百分数也不切分。

几十/m 人/n，	十几万/m 元/q，	第一百零一/m 个/q

② 约数，前加副词、形容词或后加“来、多、左右”等助数词的应予切分。

约/d 一百/m 多/m 万/m，	仅/d 一百/m 个/q，	四十/m 来/m 个/q，

二十/m 余/m 只/q，	十几/m 个/q，	三十/m 左右/m，

几十/m 人/n, 几十万/m 元/q，	近/a 20/m 年/q 来/f

两个数词相连的及“成百”、“上千”等则不予切分。

五六/m 年/q，	七八/m 天/q，	十七八/m 岁/q， 成百/m 学生/n，

上千/m 人/n，	成千上万/i 的/u 　群众/n

相连的两个数字之间若插了顿号等标点符号，还是要切分。如：

五、六年——>五/m 、/w 六/m 年/q，

九、十点钟——>九/m 、/w 十点钟/t

③ 数量词组应切分为数词和量词。

三/m 个/q, 10/m 公斤/q, 一/m 盒/q 点心/n

*但少数数量词已是词典的登录单位，则不再切分。

一个/m, 一些/m（“分词规范”中也将“一些”作为一个切分单位）

④ 表序关系的“数＋名”结构，应予切分。

如∶二/m 连/n ，　三/m 部/n

△（6） 时间词

① 年月日时分秒，按年、月、日、时、分、秒切分，标注为t 。

1997年/t 3月/t 19日/t，	98年/t 10月/t 8日/t，

3月/t 10日/t 下午/t 2时/t 18分/t

这里应注意时间词与数量词的区分，例如：“78年”指“1978年”时应标注为“78年/t”，当指数量“七十八年”时应切分标注为“78/m 年/q”。再如 两/m 个/q 月/n，三/m 天/q 时间/n 。同样，当“8日”指一个月当中的第八天时为时间词，不予切分，标注为“8日/t”；若表示8天时，则要分开，标注为“8/m 日/q”。

若数字后无表示时间的“年、月、日、时、分、秒”等的标为数词m。

中文/n 电脑/n 国际/n 会议/n ’/w 96/m

1998/m 中文/n 信息/n 处理/vn 国际/n 会议/n

*② 历史朝代的名称虽然有专有名词的性质，仍标注为t。

西周/t， 秦朝/t， 东汉/t， 南北朝/t， 清代/t

“牛年、虎年”等一律不予切分，标注为：

牛年/t、	虎年/t

“甲午年、庚子、戊戌”等也不予切分，标注为：

甲午年/t， 甲午/t 战争/n， 庚子/t 赔款/n, 戊戌/t 变法/n

△（7） 单音节代词“本”、“每”、“各”、“诸”后接单音节名词时，和后接的单音节名词合为代词；当后接双音节名词时，应予切分。

本报/r， 每人/r， 本社/r，	本/r 地区/n，	各/r 部门/n

△（8） 区别词

① 一般为切分单位，并标以词性b。

女/b 司机/n， 金/b 手镯/n， 慢性/b 胃炎/n， 古/b 钱币/n

副/b 主任/n， 总/b 公司/n

② 单音节区别词和单音节名词或名语素组合，作为一个切分单位，并标以名词词性n。

雄鸡/n， 雌象/n， 女魔/n， 古币/n

*③ 少数“单音节区别词+双音节词”的结构作为一个词收入了词典，则不再切分。

总书记/n

△⑼ 动词加动词或动词加形容词构成的述补结构

未收入词典的双音节述补结构，若拆开各是一个词，通常作为两个切分单位。

走/v 到/v，	撞/v 上/v ， 调/v 好/a， 坐/v 稳/a

若拆开了，其中至少有一个是语素，通常就不切分，作为一个切分单位。

形成/v, 鼓动/v， 说明/v， 震动/v

双音节的述补结构中间插入“得”或“不”一般应予切分，

走/v 得/u 到/v，	走/v 不/d 到/v， 安/v 得/u 上/v， 安/v 不/d 上/v

但是如果去掉“得”或“不”后，前后两个字不构成一个词的，则作为一个分词单位。

来得及/v，	来不及/v，	对得起/v， 对不起/v， 说得过去/l， 说不过去/l

有的去掉“得”或“不”后虽然是一个合成词，但其中至少有一个是语素，拆开了却是难以理解的，仍作为一个切分单位。

形得成/v，	形不成/v

*⑽ 四个字以上的短语，通常应切分。

总结/v 经验/n，	贯彻/v 执行/v，	调查/v 研究/v，

一/m 慢/a 二/m 看/v 三/m 通过/v

但像“生产资料/n ”、 “国民经济/n”、 “生产关系/n”等若作为一个词已收入词

典的就不再切分。

*⑾ 四个字的成语或习惯用语为一个切分单位，并标以词性i或l。

胸有成竹/i， 欣欣向荣/i, 众所周知/i, 由此可见/l， 新春伊始/l

⑿ 超过四个字的习惯用语或成语，一般不予切分，标注为 l或i 。

近水楼台先得月/i, 一年之计在于春/i，

不管三七二十一/i，	众人拾柴火焰高/i，	铁公鸡一毛不拔/l，

挂羊头卖狗肉/ i。

中间用标点符号分开的成语，则先切分，再用方括号括起来，标注为I。（

挂羊头，卖狗肉——> [挂羊头/i ，/w 卖狗肉/i]i

百尺竿头，更进一步——>[百尺竿头/i ，/w 更进一步/i]i

上不着天，下不着地——>[上不着天/i ，/w 下不着地/i]i

⒀ 表达一个完整概念或集合的缩略语为一个切分单位，并标以j。

三好/j，	爱委会/j， 教科文/j，	农工牧副渔业/j， 中西方/j

*在有顿号分开的情况下，则切分:

德/j 、/w 意/j 、/w 日/j, 港/j 、/w 澳/j 、/w 台/j,

港/j 、/w 澳/j 同胞/n，

林/j 、/w 牧/j 、/w 副/j 、/w 渔/j 等/u 副业/n

最后一个简称如与后面一个字（语素）可合成一个词的，则不单独切分出来。

农/j 、/w 林/j 、/w 牧/j 、/w 副/j 、/w 渔业/n

国名、地名的简称并列在一起时，即使中间没有顿号也应切分分开。

中/j 美/j 跨/v 国/n 公司/n

[京/j 津/j 唐/j 地区/n]/ns

中/j 日/j 联合/vn 公报/n

港/j 澳/j 台/j 同胞/n

用括号表示的一种特殊形式的缩略语

建（构）筑物——>建（构）筑物/j

武术馆（校）——>武术馆（校）/j

国（边）境——>国（边）境/j

厅（局）长——>厅（局）长/j

*⒁ 语素和非语素字的处理

除下列特殊情况外，语素和非语素字一般不作为切分单位。

① 某些双音节离合词分开使用，其中一个是语素，可将它标注为语素。

出/v 过/u 两/m 天/q 差/Ng, 理/v 了/u 一/m 次/q 发/Ng,

洗/v 了/u 一个/m 舒舒服服/z 的/u 澡/Vg

② 单字名词或名词性语素后接单纯方位词，通常应合成为一个处所词或时间词，但为了同“分词规范”保持一致，也为了汉外机器翻译处理的方便，这里采用以下的处理方法：

a. “单字名词 + 单字方位词”的组合，切分为两个单位。

饭/n 前/f，	树/n 上/f, 包/n 里/f，	床/n 下/f

b. “单字名词性语素字+单字方位词”的结构，合为一个处所词或时	间词。

桌/Ng 上/f --> 桌上/s，	午/Ng 后/f --> 午后/t，

身/Ng 上/f -->身上/s, 胸/Ng 前/f -->胸前/s

c. “省、市、县、乡、村、部、局、处、团、营、连、院、系、班”等名词后“里、上”等方位词，仍有组织、机构的意义，作为一个切分单位，标为名词。

如： 部里/n，	县里/n，	村里/n，	系里/n，	班上/n

③ 非语素字单独在文本中时，标注为x。

“/w 鹌鹑/n ”/w 的/u “/w 鹌/x ”/w 字/n 怎么/r 读/v ？/w

*(15) 文本中非汉字的字符串的处理意见

1. 已经约定俗成的或科学技术中已通用的符号保持原有的意义，根据其原有的意义决

定相应的标记。

阿拉伯数字：121/m 号/q 房间/n

2000年/t 8月/t 15日/t

单独的罗马数字：II/m

IX/m

xv/m

英文字母（或字母组合）代表常用的度量单位：

A代表“安培”，例句：然后指针回指在1A处

正确的切分、标注为：

然后/c 指针/n 回指/v 在/p 1/m A/q 处/n

又如V代表“伏特”；W，“瓦特”；m，米；kg，千克； 等等。

② 其他英文字母（或字母组合或语句）一律标注为nx，如：

世界杯/n 足球赛/n A/nx 组/n 的/u 两/m 场/q 比赛/vn

（这里的A起代词作用）

A/nx 公司/n ，B/nx 先生/n ，X/nx 君/Ng

（这里的A起(专有)名词或代词作用）

24/m K/nx 镀金/n

（这里的K实际上是含纯金量的度量单位，中文用“开”，计算机将它标注为nx，人又未校对出来，不算错，最好能保持一致。）

C/nx 是/v 光速/n

Windows98/nx

PentiumIV/nx

I LOVE THIS GAME/nx

(尽管这是一个英语句子，但在此阶段仍作为一个切分单位)

③ 其他西文（希腊文、俄文等）的处理同英文。

④ 日文假名处理同英文。日文中的汉字若用GB2312中的汉字，其处理同中文；

若用JIS码，则同英文。

**3. 切分和标注相结合的规范**

汉语中的语素是构词的基本单位。语素构成合成词的方式主要有三种：重叠、附加和复合。对这些情况的切分标注作如下规定。

**3.1	重叠：**

汉语以重叠变化方式构词的情况，主要有AA，AAB，ABB，AABB，A里AB，A不AB，ABAB等形式（其中A，B分别代表一个汉字），若这种词形作为词条收入了语法信息词典，其词性是确定的。下面的讨论主要是针对词典中没有该词形的情况：

⑴ “AA”重叠形

① 单字动词重叠式AA作为一个切分单位，并标注为动词词性v。

如：走走/v，听听/v

② 单字形容词重叠式AA，有的成词，有的不成词。如后面不紧跟“的”就成词，作为一个切分单位，通常为副词d。

好好/d 干/v 吧/y，	久久/d 没/d 说话/v

若后面再加“地”，不改变原有的规定，如：

轻轻/d 吊/v 起/v 又/d 轻轻/d 地/u 放/v 下/v

久久/d 地/u 没/d 说话/v

但是，如果只有紧跟着“的”或“地”才成词，则“AA的”或“AA地”合为一个

切分单位，标注为状态词z。

甜甜的/z 点心/n, 削/v 得/u 尖尖的/z，

圆圆地/z 坐/v 一/m 圈/q

③ 单字名词重叠式AA，为一个切分单位，并标注为名词词性n。

　　 　　　 人人/n，	家家/n

　 ④ 单字量词重叠形式AA，为一个切分单位，并标上量词词性q。

　　 　　　 张张/q，	个个/q

⑤ 单字副词重叠式AA，为一个切分单位，并标注为副词词性d。

　　　 　　 常常/d, 仅仅/d

⑵ “AAB”重叠形

① VO结构形式的双音节离合动词的“AAB”重叠形式为一个切分单位,并标为动词词性v。

洗洗澡/v，	挥挥手/v，	理理发/v

② 单音节动词的重叠式AA加“看”合为一个切分单位，并标注为动词词性v

试试看/v，	查查看/v，	念念看/v

⑶ “ABB”重叠形

① 双音节形容词的重叠形式ABB, 为切分单位，并标注为状态词z。

孤单单/z，	亮堂堂/z，	孤零零/z

　 ② 数量结构的“ABB”形式，不予切分，并标上数词词性m（具有数量词的属性)。

一个个/m, 一阵阵/m, 一团团/m

⑷ “AABB”重叠形

① 二字动词的重叠形式“AABB”为一个切分单位，并标注动词v。

比比划划/v，	勾勾搭搭/v

② 二字形容词的重叠形式“AABB”为一个切分单位，

高高兴兴/z，	舒舒服服/z

若后加“的”或“地”，则标注为：

高高兴兴/z 的/u，	舒舒服服/z 地/u

③ 二字名词的重叠形式“AABB”为一个切分单位，并标注为名词n。

山山水水/n，	方方面面/n

④ 二字数词的重叠形式“AABB”为一个切分单位，并标注为数词m。

许许多多/m, 多多少少/m

⑤ 有两个意义相反的单字形容词并列而成的名词再重叠所得到的重叠形式“AABB”为一个切分单位，并标注为状态词ｚ。

大大小小/z，	高高低低/z

⑥ 凡只能处于状语位置上的重叠形式“AABB” 标注为副词d。

　　　　　 日日夜夜/d，	原原本本/d，	确确实实/d

⑸ “A里AB”和“A不AB”的词形

① 双音节形容词的重叠形式“A里AB”，为一个切分单位，并标注为状态词z。

马里马虎/z，	糊里糊涂/z，	慌里慌张/z

② 用肯定加否定的形式表示疑问的动词或形容词的词组，一般切分开。

相信/v 不/d 相信/v，	容易/a 不/d 容易/a

但是如形成“A不AB”的不完整形式，则不予切分，并分别标以词性v或z。

相不相信/v, 容不容易/z，	漂不漂亮/z

⑹ “ABAB”重叠形

双音节词的重叠形式“ABAB”，都切分开，这主要包括：

① 动词的“ABAB”	如：研究/v 研究/v，	比划/v 比划/v

② 形容词的“ABAB”	如：高兴/a 高兴/a，	舒服/a 舒服/a

③ 数词的“ABAB”	如：很多/m 很多/m，	许多/m 许多/m

④ 状态词的“ABAB”	如：雪白/z 雪白/z，	碧绿/z 碧绿/z

⑤ 数量词的“ABAB”	如：一个/m 一个/m

⑺ 双音节拟声词的 “ABAB”重叠形式同其他词类一样，切分开，如：

哗啦哗啦——>哗啦/o 哗啦/o

⑻ 其他形式的重叠情况

由动词形成的“V一V，V了V，V了一V”重叠形式, 作为动词词组都切分开。

谈/v 一/m 谈/v, 想/v 了/u 想/v, 读/v 了/u 一/m	读/v

**3.2 附加**

⑴ 前接成分＋语素或词

由“前接成分＋语素或词”构成的合成词，为一个切分单位。这又可细分为以下情况：

① “阿”＋单音节名词或名语素，组成名词，并标以n；若该名语素是指人的专名，则标为nr。

如：阿哥/n，	阿华/nr

② “小”或“老”或“大”＋单音节姓氏字，组成指人专有名词，标以nr。

如：小王/nr， 老张/nr， 大杨/nr

③ “老”或“小”＋单字基数词（二，三，……，九），组成名词并标以n。

如：老二/n，老六/n，小三/n

④ 其它前接成分（“非”，“超”，“无”，“过”，……）与词构成的新的合成词，可能保持原词的词性，也可能改变词性。

如：非金属/n， 超音速/b（音速/n)，	超声波/n， 无公害/v（公害/n），

无条件/d（条件/n），	过饱和/z （饱和/a）

若“非”等前接成分所管辖的范围超过一个词，则仍然切分开。

如：非/h 国家/n 工作/vn 人员/n，	非/h 本市/r 注册/vn 车辆/n

⑵ 语素或词＋后接成分

由“语素或词＋后接成分”组成的合成词，一律为一个切分单位。 详述如下：

① #＋“儿”（#表示任意语素或词，下同）

儿化词一般为名词，如：花儿/n，画儿/n

也有例外： 一/m 堆儿/q，玩儿/v，颠儿/v，滚圆儿/z，好好儿/d， 好好儿的/z

②	#＋“们”

a. 表示名词复数的“们”单独切分，并标以k。如：

朋友/n 们/k ，孩子/n 们/k

b. 二字词中的“们”或口语中的 “们”同前面的名词的组合（可儿化）拆开了无意义，就合起来作为一个切分单位，并标以n。如：

人们/n ，哥儿们/n ，爷儿们/n， 老少/n 爷儿们/n， 老少/n 爷们儿/n

③	有类化作用的后接成分

a. 由后接成分“家”，“员”，“生”，“长(zhang3)”，“性”，“机”等组成的合成词，一般为名词。 如：艺术家/n ，办事员/n ，劳动者/n ，毕业生/n ，参谋长/n ，革命性/n ，磁盘机/n

b. 由后接成分“头(tou5)”，“子(zi5)”等组成的合成词，一般为名词，如：

对头/n ，码子/n

但也有特殊情况，如： 前头/f ，后头/f

应该注意的是，具有实在意义的“头（tou2）”、“子（zi3）”不看作后接成分，试比较：

对头/n(dui4tou5)，	对头/a(dui4tou2)

砖头/n(zhuan1tou5), 子弹/n 头/n(tou2)

桌子/n(zhuo2zi5), 围棋/n 子/n(zi3)

c. # ＋“化”， 一般组成动词，如：标准化/v， 多元化/v；也有例外：四化/j，理想化/a。

d. # ＋“者”，“者”前面为较短的词或短语时，它和前面的词一起合成一个切分单位，标注为 n；“者”前面为较长的短语或句子时，分开来，标注为 k 。

研究者/n，	探索者/n，	求知者/n，	屡教不改者/n

经过/p 苦苦/d 追求/v 而/c 获得/v 幸福/a 者/k

不/d 顾/v 劝告/v 而/c 执意/vd 闹事/v 者/k

④ 词加多个后接成分，仍为一个切分单位。

　　　　　 物理学/n, 物理学家/n，	语言学/n, 语言学界/n

⑶ 前接成分＋语素或词＋后接成分，此种形式组成的合成词，也为一个切分单位。

非党员/n，	无政府主义者/n，	超大型/b

⑷ 注意：单音节区别词与前接成分的处理方式不同，见2.2⑻。

**3.3 复合词**

“复合”方式可将两个构词成分结合成一个新词。构词成分通常认为是语素。由于复合词的构成方式和短语的构成方式是一样的，包括定中、状中、述宾、述补、主谓、联合、连动等。当语素是成词语素时，复合词与短语的界限是不清晰的。只有当构词成分中至少有一个是不成词语素时，才有把握判断新组合的结构是一个未登录词，否则存在一定的弹性。形式上，两个字的或三个字的组合可以较宽地认为是一个词。以下使用的“名”指标注为n的名词或标注为Ng的名语素。“形”,“动”的含义与可以类推。

⑴ 二字名词

① “名＋名”的定中结构， 一般为一个切分单位。

牛肉/n，	铝锅/n，	敌营/n

② “动＋名”的定中结构，一般为一个切分单位。

炒菜/n，	烤肉/n，	绑腿/n，	来函/n，	恋人/n

③ “动＋名”如为述宾结构，则是短语，应切分开。

我/r 喜欢/v 吃/v 烤肉/n 。/w

我/r 来/v 烤/v 肉/n 吃/v 。/w

但有些结合紧密或使用稳定的述宾结构已在词典中登录，则处理成一个切分单位（离合词），标注为动词v，如：吃饭/v， 洗澡/v， 讲话/v。

④ “形＋名”的定中结构，若中间不能插“的”或插“的”后意义改变，则作为一个切分单位；否则，应予切分。

红茶/n ，	苦瓜/n ，	红花/n（一种药材）

小/a 床/n , 白/a 花/n ，	红/a 花/n

⑵ 三字名词

① “动（双音）＋名（单音）”的定中结构，一般为一个切分单位。

消耗品/n, 证明信/n，	救济粮/n，	控制阀/n

② “名（双音）＋名（单音）”结构，通常为一个切分单位，但弹性较大，若前面的双音节名词与后面的单音节名词组合后意义不变，也可以分开

牛仔服/n，	电流表/n，	热带鱼/n，	河北/ns 人/n, 手表/n 厂/n

③ “名（单音）＋名（双音）”结构，通常为一个切分单位，但弹性较大，若前面的单音节名词与后面的双音节名词组合后意义不变，也可以分开

手指甲/n，	马尾巴/n, 电/n 暖壶/n

④ “形（单音）＋名（双音）”的定中结构，处理原则同二字的“形＋名”组合

小媳妇/n，	老姑娘/n

白/a 砂糖/n，	香/a 橡皮/n，	甜/a 点心/n

⑤ “形（双音）+名（单）”的定中结构，处理原则同④

美丽岛/n， 贫困/a 县/n，	富裕/a 村/n

⑶ 单纯方位词＋名（单音）的定中结构，为一个切分单位。所组成的合成词一般是处所词，但在某些特殊情况下可能是名词或时间词。

前院/s，	里屋/s，	后街/s

左肩/n，	旁杈/n，	前天/t，	后天/t

**⒋ 标注规范**

**4.1 词性标注与语法信息词典的关系**

根据《现代汉语语法信息词典》，对于那些没有兼类信息的词，在切分的同时就可以确定其词性。这样，标注规范重点描述那些多类词的词性，即在特定的上下文环境下如何选择一个正确的词性标记。

⑴ 由于自动标注的依据是语法信息词典，标注的信息绝大多数来自词典，是比较可靠的，但由于存在“多选一”和“猜测”未登录词词性这两种情况，因此词性的正确性还是需要鉴别的。

⑵ 由于上下文中信息充分，文本中的词性标注相对于词的归类要容易，但在北大的语法体系内应坚持词类的多功能性,主要防止的倾向是仅仅根据一个词在当前句子中所实现的功能来确定其词性，如将主宾语位置上的词一律定为名词，这是不恰当的。

⑶ 由于词典的空间限制，不仅存在未登录词问题，已登录的词也存在兼类不完备的问题。如有些名词可兼量词（“一/m 船/q 水/n”的“船”就是量词），词典中可能只描述它可以临时作量词，而未明确规定它兼属量词类，这时仍应以文本中的实际功能决定其词性。又如“新”，词典中只确定它是形容词，也有人认为“新同学”中的“新”是区别词，标成“新/b 同学/n”也是可以的。这样将充分发掘每个词形可能兼有的词性。至于新兼的词性以后是否收入词典则还要考虑其他因素。

**4.2 常见多类词的词性选择**

由于文本数据的特点，机器无法区分同形异音词与同形同音异类词，这就造成了汉语词类标注过程中词类歧义现象较多。可以笼统地把具有这种现象的词称为多类词。下面说明多类词的一些标注原则。

⑴ n-q多类情况。

汉语中的一些名词（主要是单音节名词）可以兼作量词，对于这些词，依据上下文来确定句子中的词的词性。

① 数词＋ n-q ＋ n，取q。

一/m 车/q 煤/n，	三/m 桶/q 水/n

另外，汉语中有一部分名词临时作量词且只能前接数词“一”，对于这种情况，也是应该把它标为量词q。

做/v 了/u 一/m 桌子/q 菜/n, 生/v 了/u 一/m 肚子/q 气/n

② “这”,“那”,“每”等指示代词＋ n-q＋n, 取q。

这/r 床/q 被子/n, 这/r 门/q 亲事/n

③ 其它情况，一般取n。

上/v 车/n， 进/v 门/n， 买/v 车/n， 送/v 桶/n 去/v 工地/s

⑵ a-v多类情况

① 若该词在句子中带了真宾语，则标为v。

他/r 跟/p 她/r 没/d 红/v 过/u 脸/n,

繁荣/v 市场/n, 端正/v 态度/n

② 若该词受“很”一类程度副词修饰，则标为a。

这/r 花/n 很/d 红/a, 市场/n 很/d 繁荣/a

③ 若该词修饰名词作定语，则一般应标为a。

繁荣/a 的/u 景象/n, 红/a 颜料/n, 巩固/a 的/u 国防/n

④ 若该词作动词的补语，则应标为a。

放/v 明白/a 一些/m

涨/v 红/a 了/u 脸/n

⑶ v-n多类情况

实际上指的是广义兼类现象。当该词表示一种动作时，后面带真宾语,则是v；当它指称人或物时，则是n。

编辑/v 科技/n 文献/n

她/r 是/v 责任/n 编辑/n

要/v 锁/v 上/v 门/n

忘/v 了/u 买/v 一/m 把/q 锁/n

及时/ad 报告/v 首长/n

一/m 份/q 重要/a 报告/n

⑷ p-v多类情况

这类词主要有“在”，“到”，“比”，“朝”，“跟”，“给”等，它们的区分主要依据以下方法：

① 从词的语法功能与分布考虑，若该词（包括带“着、了、过”的情况）单说或单独做谓语，则为动词。

“你/r 爸爸/n 在/v 不/d 在/v ？/w ” “在/v 。/w”

北京/ns 到/v 了/y ，	新加坡/ns 我/r 到/v 过/u

别/d 老/d	跟/v 着/u，	咱们/r 比/v 一/m 比/v

② 对“p-v＋其他成分” 的结构，若单说或单独作谓语，则其中的p-v为动词；若不是单说也不是单独作谓语，而是作状语或补语，则其中的p-v为介词。试比较：

| 动 词 | 介 词 |
| --- | --- |
| 他/r 不/d 在/v 教室/n | 他/r 在/p 教室/n 自习/v |
| 他/r 在/v 不/d 在/v 家/n ？——在/v | 我们/r 走/v 在/p校园/n 的/u 小路/n 上/f |
| 列车/n 已/d 到/v 了/u 北京/ns | 老王/nr 到/p 北京/ns 出差/v 去/v 了/u |
| 到/v 没/d 到/v 站/n？——到/v 了/u | 从/p 东/f 到/p 西/f 共/d 长/a 30/m 米/q |
| 狗/n 总/d 跟/v 着/u 主人/n | 我/r 常/d 跟/p 他/r 学/v 日语/n |
| 葵花/n 向/v 太阳/n | 运动员/n 正/d 跑/v 向/p 终点/n |

房子/n 朝/v 南/f	门/n 朝/p 南/f 开/v

⑸ p-c多类情况

常见的词有“和”，“跟”，“同”，“与”，这些词的词类排歧主要依据下列原则：在句子中，如果这些词的前后成分不能互换位置或者在这些词的前面可以加修饰成分，则这些词为介词；如果这些词的前后成分可以互换位置即互换位置后句子的意思基本不变并且在这些词的前面不能有修饰成分，则这些词为连词。

我/r 跟/c 他/r 都/d 是/v 大学生/n

你/r 别/d 跟/p 他/r 跑/v

我/r 跟/p 他/r 请教/v 问题/n

注意：下面的句子是有歧义的（括号内信息为判定标准）。

我/r （已经/d） 和/p 他/r 见面/v 了/y 。/w

我/r 和/c 他/r （已经/d） 见面/v 了/y 。/w

因此，需结合前后上下文信息，才能确定正确的词类标记。

⑹ b-d多类情况

① 若此词作状语, 则为副词。

我们/r 会/v 共同/d	进步/v

自动/d 取消/v 订单/n

② 若此词作定语, 或与“的”组成“的”字结构，则为区别词。

共同/b 目标/n 是/v 完成/v 这/r 项/q 任务/n

这/r 个/q 玩具/n 是/v 自动/b 的/u

⑺ c-d多类情况

这类多类词，主要有“不过”、“尽管”、“但”、“可”等。一般来说，若该词在句子中修饰谓语（形容词、动词）则为副词。若此词主要连接句子和子句，表示子句之间转折、让步等语义组合关系，则为连词。试比较：

① 不过

我/r 受/v 了/u 点/q 伤/Ng ，/w 不过/c 不/d 要紧/a

他/r 不过/d 随便/ad 谈谈/v

② 但

雨/n 停/v 了/y ，/w 但/c 地上/s 还/d 很/d 湿/a 。/w

但/d 见/v 门上/s 贴/v 着/u 一/m 副/q 对联/n 。/w

③ 可

大家/r 虽然/c 累/a ，/w 可/c 都/d 很/d 愉快/a 。/w

她/r 待/v 我/r 可/d 好/a 了/y 。

④ 尽管

尽管/c 天/n 下/v 着/u 雨/n ，/w 他/r 还是/d 出发/v 了/y 。/w

你/r 尽管/d 说/v ，/w 别/d 怕/v 。/w

4.3 **关于标记vn, vd, an, ad**

这4个标记分别是动词v和形容词a的附类标记。当将文本中的一个词标为vn、vd或 an、 ad时，首先认为它们是动词或形容词，只不过它们另有特殊的语法性质，在语句中表现特殊的语法功能。有时也赋予这些标记以中文名称，如名动词等，只是为了方便。

1 有一部分双音节动词，当它在句法结构中具有以下4种语法功能之一时，标为vn：

a. 作特殊动词“有”的宾语。

b. 充当了形式动词或其他准谓宾动词的准谓词性宾语。
c. 直接充当体词性短语的中心语。
d. 不加助词“的”，直接充当体词性短语的修饰语。

领导/n 对/p 这/r 件/q 事/n 有/v 考虑/vn

进行/v 一/m 次/q 深入/a 的/u 考察/vn

予以/v 严肃/a 处理/vn

加以/v 整理/vn

语法/n 研究/vn 很/d 重要/a

必须/d 改进/v 训练/vn 方法/n

这个/r 研究/vn 思路/n 很/d 新颖/a

需要注意，动词直接作主语或谓宾动词的宾语，仍标为v，不标作vn。

考察/v 是/v 必要/a 的/u

我们/n 来/v 的/u 目的/n 就是/v 考察/v 考察/v

需要/v 考察/v

需要/v 考察/v 实际/a 情况/n

通常只在该动词所在的短语结构的层次内决定将它标注为v还是vn。例如，

① 我们/r调查/v目的/n 是/v 了解/v 实际/a 情况/n 。/w

② 大规模/d 调查/v 语言/n 的/u 实际/a使用/vn 情况/n 是/v 一/m 项/q 重要/a

的/u 基础/n 工作/vn 。/w

③ 通过/p 调查/v

④ 通过/p 调查/v 语言/n 的/u 实际/a使用/vn 情况/n

⑤ 进行/v 调查/vn

⑥ 进行/v 大规模/b 调查/vn

⑦ 通过/p 语言/n 实际/a 使用/vn 情况/n 的/u 大规模/b 调查/vn

⑧ 通过/p 语言/n 实际/a 使用/vn 情况/n 的/u 大规模/d 调查/v

以上8个例子中对“调查”的标注都是正确的。

在①中，“我们”和“调查”首先结合成主谓结构，然后再修饰“目的”。如果在“目的”之前加一个“的”，结构更清晰，读起来更流畅。不过在书面语中，这个“的”常被省掉。如果认为“的”加在“我们”和“调查”之间，“调查”和“目的”先构成定中结构，则“调查”应标注为vn。这里有歧解。

在②中，“调查”或者先同“大规模”构成状中结构，或者先同“语言的实际使用情况”构成述宾结构，都要标成v。

在③中，“调查”本身作介词“通过”的宾语。在《规范》遵循的语法体系内，介词可以带谓词性宾语。“调查”是动词的理由可在④中找到。

在④中，“调查”先同“语言的实际使用情况”构成谓词性的述宾结构，再作介词“通过”的宾语。

在⑤中，“调查”作形式动词“进行”的准谓词性宾语，当然标成vn。

在⑥中，“进行”的准谓词性宾语“调查”可以带定语，“大规模”应该标成区别词。

在⑦和⑧中，对“大规模调查”的标注是不一样的。为什么前面说它们都对呢？首先，⑦是对的。因为从整体上看，“语言实际使用情况的大规模调查”是体词性的，将其中心语“大规模调查”也标成体词性的定中结构，不会引起争议。而在⑧中，“大规模调查”却被标成了谓词性的状中结构。这样标算不算错？理论上有没有困难？前面所说的“介词可以带谓词性宾语”是“词组本位”语法体系的一个重要论点，而这里认为“体词性短语的中心成分可以是谓词性成分”则是“词组本位”语法体系坚持的另一个更重要的、更显示其理论特色的论点。对此，朱德熙先生早有阐述。坚持这个论点，可以比较方便地分析下面的句法结构。

需要/v 支持/v

需要/v 支持/v 有/v 创造性/n 的/u 探索/vn

需要/v 群众/n 的/u 支持/vn

需要/v 群众/n 的/u 大力/d 支持/v

“群众的支持”是体词性短语，其中心语“支持”标成了vn。对vn 的完整理解应当是：

“支持”首先是动词，但在这个具体的句法位置上起名词的作用。“群众的大力支持”也是体词性短语，其中心语“大力支持”是谓词性的，是状中结构。在这个结构层次中“支持”是动词v，“大力”是副词d。由于“大力”只有一个副词词性，这样分析就不会有困难。如果主张“体词性短语的中心成分只能是体词性成分”，“支持”固然可标注为vn，但“大力”作为副词是不能修饰体词性成分的。类似的，还有：

钢/n 产量/n 的/u 逐步/d 增加/v

这里的“逐步”也只有一个副词词性。

当上下文信息不充分时，标注可能出现歧解。如上面①中的“调查”标为v或vn都不能算错。在⑦和⑧中，“大规模调查”也有两种都可以接受的标注结果。

在“现场考察是重要的”中的“现场考察”是有歧义的。有两种标法。

现场/s 考察/v 是/v 重要/a 的/u

（去/v 现场/s 考察/v 工艺/n 流程/n 是/v 重要/a 的/u）

现场/s 考察/vn 是/v 重要/a 的/u

（进行/v 一/m 次/q 现场/s 考察/vn 是/v 重要/a 的/u）

如果缺少更多的上下文，只对“现场考察是重要的”进行标注，则认为这两种标注都是正确的。

**注**：“现场”的词性是处所词s，处所词可以作状语修饰动词，也可以作定语修饰名词。

⑵ 当动词直接作状语时，标注为 vd 。

他/r 讽刺/vd 说/v

主任/n 强调/vd 指出/v

若动词后加“地”作状语，仍标为v。

他/r 讽刺/v 地/u 说/v

主任/n 强调/v 地/u 指出/v

3 同一部分动词具有名词特点一样，当一部分形容词在实际语料中具有以下3种语法功能之一时，也认为它具有名词特点，标注为an。
a. 作了“有”的宾语，
b. 充当了准谓宾动词的准谓词性宾语，

c. 直接充当体词性短语的中心语。

他/r 有/v 很多/m 苦恼/an

这里/s 有/v 奥妙/an

维护/v 环境/n 的/u 整洁/an

交通/n 安全/an 是/v 第一/m 要/v 注意/v 的/u

需要注意，形容词直接作主语或谓宾动词的宾语，仍标为a，不标作an 。

需要/v 努力/a

需要/v 进一步/d 努力/a

⑷ 形容词直接作状语时，标注为 ad 。

认真/ad 学习/v 邓小平理论/n

深入/ad 研究/v 语法/n 有利/a 于/p 自然/a 语言/n 处理/vn 技术/n 的/u 进步/vn

形容词后接“地”作状语时，那形容词仍标注为 a 。

我们/r 应当/v 深入/a 地/u 研究/v 语法/n

**5. 后记**

本规范由俞士汶提出并主持制订。陆俭明、朱学锋、段慧明、亢世勇、孙宏林、王惠、赵强、詹卫东等人参加了本规范的制订。俞士汶起草了本规范的征求意见稿，陆俭明教授对征求意见稿进行了字斟句酌的修改。随后，有了试用稿。在长达6个多月的制订、试用过程中，稿子几经修订，还吸收了北大中文系郭锐副教授的一些意见，形成了“1999年3月版”。除北大人员外，富士通公司的专家和技术人员提出了有关专有名词加工的基本要求并参与了本规范的制订。

在加工1998年《人民日报》前6个月语料的过程中，富士通公司的专家和技术人员通过验收北大计算语言学研究所提供的成果对语料加工的一致性问题提出了宝贵的意见，协作单位上海师范大学、烟台师范学院、南京师范大学、北京语言文化大学、中国语文现代化学会等单位的老师和同学也提出了一些意见。北大计算语言学研究所俞士汶、段慧明、朱学锋、孙斌等吸收这些意见，对“1999年3月版”进行了修订，形成现在的“2001年7月版”

欢迎对本规范继续提出批评和指正。经过更多的实践后，再考虑进一步修改和补充。
