# Supplementary material for: Constructing marine expert management knowledge graph based on Trellisnet-CRF
Source: PeerJ Comput Sci. 2022 Sep 5;8:e1083. doi: 10.7717/peerj-cs.1083 (PMC9455288; doi:10.7717/peerj-cs.1083)
Supplement: Supplemental Information 2 [file peerj-cs-08-1083-s002.zip › Peopleí»s Daily Corpus-raw data/corpus_Guideline/Guideline_contents_Chinese.doc]

目	录

现代汉语语料库加工规范——词语切分与词性标注

⒈ 前言………………………………………………………… 1

⒉ 切分规范…………………………………………………… 3

2.1 基本概念………………………………………………… 3

2.2 对分词规范的补充和调整………………………………… 4

⒊ 切分和标注相结合的规范…………………………………12

3.1 重叠………………………………………………………12

3.2 附加………………………………………………………14

3.3 复合词……………………………………………………15

⒋ 标注规范……………………………………………………16

4.1 词性标注与语法信息词典的关系……………………………16

4.2 常见多类词的词形选择……………………………………16

4.3 关于标记vn, vd, an, ad …………………………………18

⒌ 后记…………………………………………………………21

附录：

⒈ 按代码的字母顺序排列的标记集…………………………22

⒉ 按名称的汉语拼音顺序排列的标记集……………………24

⒊ 参考文献……………………………………………………25
