# Supplementary material for: Constructing marine expert management knowledge graph based on Trellisnet-CRF
Source: PeerJ Comput Sci. 2022 Sep 5;8:e1083. doi: 10.7717/peerj-cs.1083 (PMC9455288; doi:10.7717/peerj-cs.1083)
Supplement: Supplemental Information 2 [file peerj-cs-08-1083-s002.zip › Peopleí»s Daily Corpus-raw data/corpus_readme_Chinese.doc]

**人民日报标注语料库（PFR）使用说明书**

本文是PFR标注语料库的使用说明书，帮助用户了解它，更好地使用它。

PFR语料库是对人民日报1998年上半年的纯文本语料进行了词语切分和词性标注制作而成的，严格按照人民日报的日期、版序、文章顺序编排的。文章中的每个词语都带有词性标记。目前的标记集里有26个基本词类标记（名词n、时间词t、处所词s、方位词f、数词m、量词q、区别词b、代词r、动词v、形容词a、状态词z、副词d、介词p、连词c、助词u、语气词y、叹词e、拟声词o、成语i、习惯用语l、简称j、前接成分h、后接成分k、语素g、非语素字x、标点符号w）外，从语料库应用的角度，增加了专有名词（人名nr、地名ns、机构名称nt、其他专有名词nz）；从语言学角度也增加了一些标记，总共使用了40多个个标记。

1. 标记简要说明

| 代码 | 名称 |
| --- | --- |
| Ag | 形语素 |
| a | 形容词 |
| ad | 副形词 |
| an | 名形词 |
| Bg | 区别语素 |
| b | 区别词 |
| c | 连词 |
| Dg | 副语素 |
| d | 副词 |
| e | 叹词 |
| f | 方位词 |
| g | 语素 |
| h | 前接成分 |
| i | 成语 |
| j | 简略语 |
| k | 后接成分 |
| l | 习用语 |
| Mg | 数语素 |
| m | 数词 |
| Ng | 名语素 |
| n | 名词 |
| nr | 人名 |
| ns | 地名 |
| nt | 机构团体 |
| nx | 外文字符 |
| nz | 其它专名 |
| o | 拟声词 |
| p | 介词 |
| Qg | 量语素 |
| q | 量词 |
| Rg | 代语素 |
| r | 代词 |
| s | 处所词 |
| Tg | 时间语素 |
| t | 时间词 |
| Ug | 助语素 |
| u | 助词 |
| Vg | 动语素 |
| v | 动词 |
| vd | 副动词 |
| vn | 名动词 |
| w | 标点符号 |
| x | 非语素字 |
| Yg | 语气语素 |
| y | 语气词 |
| z | 状态词 |

1. 格式说明
   1． 语料是纯文本文件，文件中每一行代表一自然段或者一个标题，一篇文章有若干个自然段，因此在语料中一篇文章是由多行组成的。
   2． 每一行的开头是编号。比如“19980101-01-001-001”表示这一自然段是1998年1月1日的第01版的第001篇文章的第001自然段，用短横线隔开的4部分按照顺序是“年月日-版号-篇章号-段号”。标号也作为一个词进行标注，词性固定为“m（数词）”。
   3． 一篇文章里面的段落之间是不空行的，在两篇文章之间，会有一个空行，表示文章的分界线，同时，下一篇文章的“篇章号-段号”都会有所改变。
   4． 标号之后，是2个单字节空格，然后开始正文。
   5． 正文部分按照规范已经切分成词，并且加上标注，标注的格式为“词语/词性”，即词语后面加单斜线，再紧跟词性标记。词与词之间用2个单字节空格隔开。每段最后的词，在标记之后也有2个单字节空格，保持格式一致。
   6． 语料中除了词性标记以外，还有“短语标记”，这种情况一般出现在机构团体名称、成语等情况中。如“通过/p [中央/n 人民/n 广播/vn 电台/n]nt 、/w”中，用“[ ]”合起来的部分是一个完整的机构团体名称，方括号后面紧跟标注nt，nt之后空两个单字节空格，保持了格式的一致。
2. 例子

19980101-01-001-001/m 迈向/v 充满/v 希望/n 的/u 新/a 世纪/n ——/w 一九九八年/t 新年/t 讲话/n （/w 附/v 图片/n １/m 张/q ）/w

……

19980101-01-001-006/m 在/p １９９８年/t 来临/v 之际/f ，/w 我/r 十分/m 高兴/a 地/u 通过/p [中央/n 人民/n 广播/vn 电台/n]nt 、/w [中国/ns 国际/n 广播/vn 电台/n]nt 和/c [中央/n 电视台/n]nt ，/w 向/p 全国/n 各族/r 人民/n ，/w 向/p [香港/ns 特别/a 行政区/n]ns 同胞/n 、/w 澳门/ns 和/c 台湾/ns 同胞/n 、/w 海外/s 侨胞/n ，/w 向/p 世界/n 各国/r 的/u 朋友/n 们/k ，/w 致以/v 诚挚/a 的/u 问候/vn 和/c 良好/a 的/u 祝愿/vn ！/w
